# Supplementary material for: Major dietary patterns of community dwelling adults and their associations with impaired blood glucose and central obesity in Eastern Ethiopia: Diet-disease epidemiological study
Source: PLoS One. 2023 Apr 20;18(4):e0283075. doi: 10.1371/journal.pone.0283075 (PMC10118135; doi:10.1371/journal.pone.0283075)
Supplement: S1 File — (DOCX) [file pone.0283075.s001.docx]

**Supplementary File 1. Semi-quantitative food frequency questionnaire**

**Instruction** –Dear respondent please take few moments to memorize the food and drinks you ate within the last month. These relate to your daily use of food items and also food consumed out of home, e.g., in a restaurant, bar, at work etc. I will say the food items if you consumed the food type you will tell me how often and how much you ate over the last month. When I ask you about your usual portion size you will tell me the average quantity or portion of the food item on the day of consumption, expressed as common household measures such as a ladle, small cup or spoon. We will use pictures to improve your response on the food items consumed.

**If you eat for instance** ‘nech teff injera’ during four days per week (Monday, Tuesday, Wednesday, and Thursday) you will choose the option that says 4-6 times per week. If you usually consume two (2) full medium sized (eight kurtih) nech teff injera on these days your answer will be 2 full medium sized enjera or eight kurtih ( if you use kurtis for serving). You will tell me the total injeras you consumed be it alone or in combination with others. (Examples, bado injera + injera be wot + injera be avocado)

**Remember**: these two full injeras are the totals of all meals (breakfast, lunch, snacks, and dinner)

1. Never or < 1 x per month
2. 1 x per month
3. 2 - 3 x per month
4. 1 x per week
5. 2 - 3 x per week
6. 4 - 6 x per week
7. Every day

**Cereal’s products, bread and potatoes**

| **Cereals**  Food item | Average consumption over the last month | | | | | | | | |
| --- | --- | --- | --- | --- | --- | --- | --- | --- | --- |
|  | 1 | 2 | 3 | 4 | 5 | 6 | 7 | Remark |  |
| **Injera**  1. Neche teff injera  2. key teff injera  3. teff injera mixed with rice  4. tef injera mixed with maize  5. **teff injera mixed with sorghum** |  |  |  |  |  |  |  |  |  |
| 6. Injera fir fir |  |  |  |  |  |  |  |  |  |
| 7. Pasta |  |  |  |  |  |  |  |  |  |

| Food item |  |  |  |  |  |  |  |  |
| --- | --- | --- | --- | --- | --- | --- | --- | --- |
| 8. Macaroni |  |  |  |  |  |  |  |  |
| 9. Rice |  |  |  |  |  |  |  |  |
| 10. kinche (aja, barley or wheat) |  |  |  |  |  |  |  |  |
| 11. Nifro (ye sinde) |  |  |  |  |  |  |  |  |
| 12. ye bula genfo |  |  |  |  |  |  |  |  |
| 13. Genfo (barley, wheat porridge) |  |  |  |  |  |  |  |  |
| **kolo**  14. ye gebsih kolo  15. ye gebsih ena ye shinbira |  |  |  |  |  |  |  |  |
| 16. Gruel (atmit) |  |  |  |  |  |  |  |  |

| **Bread** Food item | 1 | 2 | 3 | 4 | 5 | 6 | 7 | Remark |
| --- | --- | --- | --- | --- | --- | --- | --- | --- |
| 17. Bread: wheat or maize |  |  |  |  |  |  |  |  |
| 18. Bread white (nech ye sukih/ye furno duket dabo) |  |  |  |  |  |  |  |  |
| 19. Sanbusa |  |  |  |  |  |  |  |  |
|  |  |  |  |  |  |  |  |  |
| **Potatoes**  Food item |  |  |  |  |  |  |  |  |
| 20. Potato boiled (with nifro or selata) |  |  |  |  |  |  |  |  |
| 21. Potato fried |  |  |  |  |  |  |  |  |
| 22. Potato stew |  |  |  |  |  |  |  |  |

**Legumes and pulses**

| Food item | 1 | 2 | 3 | 4 | 5 | 6 | 7 | Remark |
| --- | --- | --- | --- | --- | --- | --- | --- | --- |
| **Pea stew/Shiro wet**  23. Mitin shiro  **24. ye Bakila, shimbra or ater shiro**  **25. Ye abesh wot** |  |  |  |  |  |  |  |  |
| 26. Misirkik/aterkik wet/ split lentil stew |  |  |  |  |  |  |  |  |
| 27. Aterkik wet |  |  |  |  |  |  |  |  |
| 28. Ful (ye bakela) |  |  |  |  |  |  |  |  |

**Roots and tubers**

| Food item | 1 | 2 | 3 | 4 | 5 | 6 | 7 | Remark |
| --- | --- | --- | --- | --- | --- | --- | --- | --- |
| 29. Kocho |  |  |  |  |  |  |  |  |
| 30. Beet root stew (keysir wot) |  |  |  |  |  |  |  |  |
| 31. Sweet potato |  |  |  |  |  |  |  |  |
| 32. Carrot with potato stew/Carrot be Dinich wot |  |  |  |  |  |  |  |  |

**Vegetables**

| Food item |  |  |  |  |  |  |  |  |
| --- | --- | --- | --- | --- | --- | --- | --- | --- |
| 33. Cabbage (tikilgomen) |  |  |  |  |  |  |  |  |
| 34. Kale (tikurgomen) |  |  |  |  |  |  |  |  |
| 35. Kosta |  |  |  |  |  |  |  |  |
| 36. Tomato sauce/Timatimsilsih |  |  |  |  |  |  |  |  |
| 37. Tomato chopped/Timatim kurtih |  |  |  |  |  |  |  |  |

| 38. Pumpkin stew/duba wot |  |  |  |  |  |  |  |  |
| --- | --- | --- | --- | --- | --- | --- | --- | --- |
| 39. Fosoliya |  |  |  |  |  |  |  |  |
| 40. Vegetable soup / ye atikiltihshorba |  |  |  |  |  |  |  |  |
| Fruits |  |  |  |  |  |  |  |  |
| 41. Banana |  |  |  |  |  |  |  |  |
| 42. Orange |  |  |  |  |  |  |  |  |
| 43. Mango |  |  |  |  |  |  |  |  |
| 44. Avocado |  |  |  |  |  |  |  |  |
| 45. Papaya |  |  |  |  |  |  |  |  |
| Egg |  |  |  |  |  |  |  |  |
| 46. Chicken eggs (boiled or fried) |  |  |  |  |  |  |  |  |
| **Milk and dairy** |  |  |  |  |  |  |  |  |
| 47. Milk: cow’s, camel) |  |  |  |  |  |  |  |  |
| 48. Cheese |  |  |  |  |  |  |  |  |
| 49. Yoghurt |  |  |  |  |  |  |  |  |

**Fish and products based on fish**

| Food item |  |  |  |  |  |  |  |  |
| --- | --- | --- | --- | --- | --- | --- | --- | --- |
| **Fish**  50. Asa gulash, stew, tibs, lebleb, kotelet  51. Tuna |  |  |  |  |  |  |  |  |

**Meat and poultry**

| Food item |  |  |  |  |  |  |  |  |
| --- | --- | --- | --- | --- | --- | --- | --- | --- |
| **Meat**  52. Siga wote, minchet, kikil |  |  |  |  |  |  |  |  |
| 53. Kitfo |  |  |  |  |  |  |  |  |
| 54. Gored gored/Tire sega |  |  |  |  |  |  |  |  |
| 55. Ye berehtibsih |  |  |  |  |  |  |  |  |
| **56.** Ye begih/feyel tibs |  |  |  |  |  |  |  |  |
| **Poultry (ye dorosiga)**  57. Chicken stew/doro wet  58. Doro alicha  59. Doro tibs/alecha |  |  |  |  |  |  |  |  |
| **Fat and oils** |  |  |  |  |  |  |  |  |
| **60. Butter, shenolega** |  |  |  |  |  |  |  |  |
| 61. Oil (type of oil)  Plant/palm/ saturated/ yerega |  |  |  |  |  |  |  |  |
| **Sweets** |  |  |  |  |  |  |  |  |

|  | (tire siga) |  |  |  |  |  |  |  |  |
| --- | --- | --- | --- | --- | --- | --- | --- | --- | --- |
| 44 | Sheep meat  roasted (yebeg tibs |  |  |  |  |  |  |  |  |
| 45 | Goat tibis |  |  |  |  |  |  |  |  |
| 46 | Tuna |  |  |  |  |  |  |  |  |
| 47 | Roasted fish |  |  |  |  |  |  |  |  |
|  | **Dairy fats**  **and egg** |  |  |  |  |  |  |  |  |
| 48 | Raw cow/camel Milk |  |  |  |  |  |  |  |  |
| 49 | yoghurt |  |  |  |  |  |  |  |  |
| 50 | Cottage  cheese (Ayib) |  |  |  |  |  |  |  |  |
| 51 | Egg whole  Boiled/fried |  |  |  |  |  |  |  |  |
| 52 | Egg fried |  |  |  |  |  |  |  |  |
|  | Egg with potato (selata) |  |  |  |  |  |  |  |  |
|  | **Sweets** |  |  |  |  |  |  |  |  |
| 53 | Honey |  |  |  |  |  |  |  |  |
| 54 | Sugar |  |  |  |  |  |  |  |  |
| 55 | Jams,marmara |  |  |  |  |  |  |  |  |
|  | Khalawa, Baklaba and other sweety foods |  |  |  |  |  |  |  |  |
|  | **Beverage and**  **Fast foods** |  |  |  |  |  |  |  |  |
| 56 | Mirinda/Fanta |  |  |  |  |  |  |  |  |
| 57 | Coca cola |  |  |  |  |  |  |  |  |
| 58 | Sprite |  |  |  |  |  |  |  |  |
|  | Other soft drinks; moringa, |  |  |  |  |  |  |  |  |
| 59 | Tea |  |  |  |  |  |  |  |  |
| 60 | Coffee |  |  |  |  |  |  |  |  |
| 61 | Macchiato |  |  |  |  |  |  |  |  |
| 62 | Beer |  |  |  |  |  |  |  |  |
| 63 | Tella |  |  |  |  |  |  |  |  |
|  | Other alcoholic drinks; vodka, woyne, *areki,* |  |  |  |  |  |  |  |  |

| Sweets: Food item |  |  |  |  |  |  |  |  |  |
| --- | --- | --- | --- | --- | --- | --- | --- | --- | --- |
| 72. Honey  73. Jam |  |  |  |  |  |  |  |  |  |
| 74. Sugar |  |  |  |  |  |  |  |  |  |
| Drinks |  |  |  |  |  |  |  |  |  |
| **Non-Alcoholic drinks**  Food item |  |  |  |  |  |  |  |  |  |
| 75. Coffee |  |  |  |  |  |  |  |  |  |
| 76. Tea |  |  |  |  |  |  |  |  |  |
| 77. Mekiyato/ buna be wetet |  |  |  |  |  |  |  |  |  |
| **Soft drinks**  78. Mirinda/ Fanta  79.  80. Pepsi/ Coca-cola  81. |  |  |  |  |  |  |  |  |  |
| **Alcoholic drinks**  82. Beer |  |  |  |  |  |  |  |  |  |
| 83. Tella  84. Tej  85. Arake  86. borde |  |  |  |  |  |  |  |  |  |
| **Food items: Fast foods and pastry** |  |  |  |  |  |  |  |  |  |
| 87. Burger |  |  |  |  |  |  |  |  |  |
| 88. Pizza |  |  |  |  |  |  |  |  |  |
| 89. Cake (types of cake) |  |  |  |  |  |  |  |  |  |

| 64 | Tej |  |  |  |  |  |  |  |  |  |  |  |
| --- | --- | --- | --- | --- | --- | --- | --- | --- | --- | --- | --- | --- |
| 65 | Wine |  |  |  |  |  |  |  |  |  |  |  |
| 66 | Pizza |  | In number |  |  |  |  |  |  |  |  |  |
| 67 | Burger |  | In number |  |  |  |  |  |  |  |  |  |
