# Supplementary material for: Major dietary patterns of community dwelling adults and their associations with impaired blood glucose and central obesity in Eastern Ethiopia: Diet-disease epidemiological study
Source: PLoS One. 2023 Apr 20;18(4):e0283075. doi: 10.1371/journal.pone.0283075 (PMC10118135; doi:10.1371/journal.pone.0283075)
Supplement: S2 File — (DOCX) [file pone.0283075.s002.docx]

| **S.no** | **Frequency of consumption** | **Daily frequency of consumption [times/day]** |
| --- | --- | --- |
| 1 | Never or < 1 x per month | 0.025 |
| 2 | 1 x per month | 0.033 |
| 3 | 2 - 3 x per month | 0.083 |
| 4 | 1 x per week | 0.143 |
| 5 | 2 - 3 x per week | 0.357 |
| 6 | 4 - 6 x per week | 0.71 |
| 7 | Every day | 1 |

**Supplementary File 2.** Conversion factors to a daily frequency of food consumption for adults
