# Supplementary material for: Major dietary patterns of community dwelling adults and their associations with impaired blood glucose and central obesity in Eastern Ethiopia: Diet-disease epidemiological study
Source: PLoS One. 2023 Apr 20;18(4):e0283075. doi: 10.1371/journal.pone.0283075 (PMC10118135; doi:10.1371/journal.pone.0283075)
Supplement: S3 File — (DOCX) [file pone.0283075.s003.docx]

**Supplementary File 3.** Identified major dietary patterns (components) and their factor loading for individual food item among residing in Dire Dawa and Harar, Eastern Ethiopia

| DP_1 (animal source foods, fruits, and vegetables)-Nutrient dense foods | | DP_2 (high ft and protein foods) | | DP_3 (processed foods) | | DP_4 (Traditional alcoholic drinks) | | DP_5 (cereal foods) | |
| --- | --- | --- | --- | --- | --- | --- | --- | --- | --- |
| Raw meat | 0.925 | Fish | 0.894 | White bread | 0.693 | Araki | 0.936 | Porridge from wheat | 0.764 |
| Meat roasted | 0.922 | Cheese | 0.822 | Potato products | 0.679 | Tela | 0.934 | Gruel | 0.756 |
| Poultry stewed | 0.917 | Butter | 0.746 | Rice | 0.638 | Teji | 0.924 | Brown bread | 0.691 |
| Kitfo | 0.917 | Beets | 0.675 | Pasta | 0.624 | Boredi | 0.666 | Barley roasted | 0.441 |
| Meat stew | 0.917 | Kocho | 0.659 | Macaroni | 0.592 |  |  |  |  |
| Papaya | 0.907 | Burgers | 0.630 | Samosa | 0.549 |  |  |  |  |
| Orange | 0.905 | Sweet potato | 0.615 | Pepsi/coca drinks | 0.522 |  |  |  |  |
| Mango | 0.895 | Carrot stewed | 0.545 |  |  |  |  |  |  |
| Milk | 0.892 | Beans | 0.446 |  |  |  |  |  |  |
| Avocado | 0.877 |  |  |  |  |  |  |  |  |
| Egg fried | 0.845 |  |  |  |  |  |  |  |  |
| Fanta and Mirinda drinks | 0.837 |  |  |  |  |  |  |  |  |
| Banana | 0.824 |  |  |  |  |  |  |  |  |
| Pizza | 0.737 |  |  |  |  |  |  |  |  |
| Cabbage | 0.577 |  |  |  |  |  |  |  |  |
| Vegetable soup | 0.558 |  |  |  |  |  |  |  |  |
