# Supplementary material for: Major dietary patterns of community dwelling adults and their associations with impaired blood glucose and central obesity in Eastern Ethiopia: Diet-disease epidemiological study
Source: PLoS One. 2023 Apr 20;18(4):e0283075. doi: 10.1371/journal.pone.0283075 (PMC10118135; doi:10.1371/journal.pone.0283075)
Supplement: Attachment — Submitted filename: PONE-D-22-28499_review er Commented.docx [file pone.0283075.s004.docx]

**PLOS ONE**

**Major dietary patterns of community dwelling adults and their associations with impaired blood glucose and central obesity in Eastern Ethiopia: diet-disease exploration study**

--Manuscript Draft--

**Manuscript Number:**	PONE-D-22-28499

**Article Type:**	Research Article

**Full Title:**	Major dietary patterns of community dwelling adults and their associations with impaired blood glucose and central obesity in Eastern Ethiopia: diet-disease exploration study

**Short Title:**	Major dietary patterns and their associations with impaired blood glucose and central obesity

**Corresponding Author:**	Abdu Oumer

Dire Dawa University Dire Dawa, ETHIOPIA

**Keywords:**	Central obesity; dietary intake; dietary pattern; impaired blood glucose; prediabetes

**Abstract:**	Backgrounds: Unhealthy dietary intake is an important preventable risk factor for obesity and impaired blood glucose (IBG), ultimately increasing the risk of non- communicable diseases. When compared to individual food intakes, dietary patterns are a stronger predictor of health outcomes and should be systematically evaluated where such evidence is lacking. This study evaluated dietary patterns and their association with the risk of central obesity and IBG among adults.

Methods: A community-based survey was conducted among 501 randomly-selected adults from Eastern Ethiopia. Data was collected using a semi-structured questionnaire during a face-to-face interview that included sociodemographic and lifestyle factors, as well as a validated 89-item food frequency questionnaire (collected over one month). Principal component analysis was used to derive the dietary pattern. While central obesity was assessed using waist and/or hip circumference measurements, fasting blood sugar was used for IBG. A multivariable logistic regression model was fitted with an odds ratio, 95% confidence intervals, and p-values reported.

Results: A total of 501 adults (95.3%) were interviewed, with a mean age of 41 years (±12). Five major dietary patterns explaining 71% of the total variance were identified: “nutrient-dense foods”, “high fat and protein”, “processed foods”, “alcohol drinks”, and “cereal diets”. While 20.4% (17.0—24.2%) had IBG, 14.6% (11.8—17.9) were centrally obese, and 94.6 (92.3—96.3) had an increased risk for cardiovascular complications. Central obesity is associated with upper wealth status (AOR = 6.92; 2.91—16.5), physical inactivity (AOR = 21.1; 2.77—161.4), a diet high in nutrient-dense foods

(AOR

= 1.75; 0.75—4.06), processed foods (AOR = 1.41; 0.57—3.48), and cereal diets (AOR

= 4.06; 1.87—8.82). The risk of IBG was associated with upper wealth status (AOR = 2.36; 1.36—4.10), physical inactivity (AOR = 2.17; 0.91—5.18), upper tercile of nutrient-dense foods (AOR = 1.35; 0.62—2.93), fat and protein diet (AOR = 1.31; 0.66—2.62), and cereal diet consumption (AOR = 3.87; 1.66—9.02).

Conclusion: IBG and central obesity were prevalent and predicted by upper tercile consumption of nutrient-dense foods, high fat and protein diets, processed foods, and cereal diets, which could guide dietary interventions.

**Order of Authors:**	Berhanu Abebaw Abdu Oumer Ahmed Ale Aragaw Hamza

**Additional Information:**

**Question	Response**

**Financial Disclosure**	This study was funded by Dire Dawa University, under annual competitive research

grant. The funders had no role in study design, data collection and analysis, the

*Powered by Editorial Manager® and ProduXion Manager® from Aries Systems Corporation*

| Enter a financial disclosure statement that describes the sources of funding for the work included in this submission. Review the [submission guidelines](http://journals.plos.org/plosone/s/submission-guidelines#loc-financial-disclosure-statement) for detailed requirements. View published research articles from [*PLOS ONE*](http://journals.plos.org/plosone/) for specific examples.  This statement is required for submission and **will appear in the published article** if the submission is accepted. Please make sure it is accurate. | decision to publish, or the preparation of the manuscript. |
| --- | --- |
| **Unfunded studies**  Enter: *The author(s) received no specific funding for this work.*  **Funded studies**  Enter a statement with the following details:   - Initials of the authors who received each award - Grant numbers awarded to each author - The full name of each funder - URL of each funder website - Did the sponsors or funders play any role in the study design, data collection and analysis, decision to publish, or preparation of the manuscript? - **NO** - Include this sentence at the end of your statement: *The funders had no role in*   *study design, data collection and analysis, decision to publish, or preparation of the manuscript.*   - **YES** - Specify the role(s) played. |  |
| * typeset |  |
| **Competing Interests**  Use the instructions below to enter a competing interest statement for this submission. On behalf of all authors, disclose any [competing interests](http://journals.plos.org/plosone/s/competing-interests) that could be perceived to bias this work—acknowledging all financial support and any other relevant financial or non- financial competing interests.  This statement is required for submission and **will appear in the published article** if the submission is accepted. Please make | The authors have declared that no competing interests exist. |

*Powered by Editorial Manager® and ProduXion Manager® from Aries Systems Corporation*

| sure it is accurate and that any funding sources listed in your Funding Information later in the submission form are also declared in your Financial Disclosure statement.  View published research articles from  [*PLOS ONE*](http://journals.plos.org/plosone/) for specific examples. |  |
| --- | --- |
| **NO authors have competing interests**  Enter: *The authors have declared that no competing interests exist.*  **Authors with competing interests**  Enter competing interest details beginning with this statement:  *I have read the journal's policy and the authors of this manuscript have the following competing interests: [insert competing interests here]* |  |
| * typeset |  |
| **Ethics Statement**  Enter an ethics statement for this submission. This statement is required if the study involved:   - Human participants - Human specimens or tissue - Vertebrate animals or cephalopods - Vertebrate embryos or tissues - Field research   Write "N/A" if the submission does not require an ethics statement.  General guidance is provided below. Consult the [submission guidelines](http://journals.plos.org/plosone/s/submission-guidelines#loc-guidelines-for-specific-study-types) for detailed instructions. **Make sure that all information entered here is included in the Methods section of the manuscript.** | Ethical approval was obtained from the Institutional Research Ethical Review Board of Dire Dawa University. Written informed assent was obtained from all adults This research was conducted in accordance with the Helsinki Declaration for protecting human study subjects. |

*Powered by Editorial Manager® and ProduXion Manager® from Aries Systems Corporation*

| **Format for specific study types**  **Human Subject Research (involving human participants and/or tissue)**   - Give the name of the institutional review   board or ethics committee that approved the study   - Include the approval number and/or a statement indicating approval of this research - Indicate the form of consent obtained (written/oral) or the reason that consent was not obtained (e.g. the data were analyzed anonymously)   **Animal Research (involving vertebrate animals, embryos or tissues)**   - Provide the name of the Institutional Animal Care and Use Committee (IACUC) or other relevant ethics board that reviewed the study protocol, and indicate whether they approved this research or granted a formal waiver of ethical approval - Include an approval number if one was obtained - If the study involved *non-human primates*, add *additional details* about animal welfare and steps taken to ameliorate suffering - If anesthesia, euthanasia, or any kind of animal sacrifice is part of the study, include briefly which substances and/or methods were applied   **Field Research**  Include the following details if this study involves the collection of plant, animal, or other materials from a natural setting:   - Field permit number - Name of the institution or relevant body that granted permission |  |
| --- | --- |
|  |  |
| **Data Availability**  Authors are required to make all data underlying the findings described fully available, without restriction, and from the time of publication. PLOS allows rare exceptions to address legal and ethical concerns. See the [PLOS Data Policy](http://journals.plos.org/plosone/s/data-availability) and [FAQ](http://journals.plos.org/plosone/s/data-availability#loc-faqs-for-data-policy) for detailed information. | Yes - all data are fully available without restriction |

*Powered by Editorial Manager® and ProduXion Manager® from Aries Systems Corporation*

| A Data Availability Statement describing where the data can be found is required at submission. Your answers to this question constitute the Data Availability Statement and **will be published in the article**, if accepted. |  |
| --- | --- |
| **Important:** Stating ‘data available on request from the author’ is not sufficient. If your data are only available upon request, select ‘No’ for the first question and explain your exceptional  situation in the text box. |  |
| Do the authors confirm that all data underlying the findings described in their manuscript are fully available without restriction? |  |
| **Describe where the data may be found in full sentences. If you are copying our sample text, replace any instances of XXX with the appropriate details.**   - If the data are **held or will be held in a public repository**, include URLs, accession numbers or DOIs. If this information will only be available after acceptance, indicate this by ticking the box below. For example: *All XXX files*   *are available from the XXX database (accession number(s) XXX, XXX.*).   - If the data are all contained **within the**   **manuscript and/or Supporting Information files**, enter the following: *All relevant data are within the*  *manuscript and its Supporting Information files.*   - If neither of these applies but you are   able to provide **details of access elsewhere**, with or without limitations, please do so. For example:  *Data cannot be shared publicly because of [XXX]. Data are available from the XXX Institutional Data Access / Ethics Committee (contact via XXX) for researchers who meet the criteria for access to confidential data.*  *The data underlying the results presented in the study are available from (include the name of the third party* | All relevant data are included within the manuscript and its supporting Information files. |

*Powered by Editorial Manager® and ProduXion Manager® from Aries Systems Corporation*

| *and contact information or URL).*   - This text is appropriate if the data are owned by a third party and authors do not have permission to share the data.   * typeset |  |
| --- | --- |
| Additional data availability information: |  |

*Powered by Editorial Manager® and ProduXion Manager® from Aries Systems Corporation*

Manuscript	[Click here to access/download;Manuscript;Dietary Pattern_IBG_manuscript.docx](https://www.editorialmanager.com/pone/download.aspx?id=32048940&amp;guid=fbb187b7-b439-4208-85e3-2d70cf43d7e5&amp;scheme=1)

# Major dietary patterns of community dwelling adults and their associations with impaired blood glucose and central obesity in Eastern Ethiopia: diet-disease exploration study

Berhanu Abebaw^1^, Abdu Oumer^2*^, Ahmed Ale^3^, and Aragaw Hamza^4^

1 Department of Nutrition and dietetics, School of Public Health, Bahir Dar University, Ethiopia

^2^Department of Public Health, College of Medicine and Health Sciences, Dire Dawa University, Dire Dawa, Ethiopia

^3^School of Medicine, College of Medicine and Health Sciences, Dire Dawa University, Dire Dawa, Ethiopia

^4^Department of Anesthesia, College of Medicine and Health Sciences, Dire Dawa University, Dire Dawa, Ethiopia

*Corresponding author:* Abdu Oumer; department of Public Health, College of Medicine and Health Sciences, Dire Dawa University, Dire Dawa, Ethiopia; Email: [omab2320@gmail.com](mailto:omab2320@gmail.com)

Abstract

Backgrounds: Unhealthy dietary intake is an important preventable risk factor for obesity and impaired blood glucose (IBG), ultimately increasing the risk of non-communicable diseases. When compared to individual food intakes, dietary patterns are a stronger predictor of health outcomes and should be systematically evaluated where such evidence is lacking. This study evaluated dietary patterns and their association with the risk of central obesity and IBG among adults.

Methods: A community-based survey was conducted among 501 randomly-selected adults from Eastern Ethiopia. Data was collected using a semi-structured questionnaire during a face-to-face interview that included sociodemographic and lifestyle factors, as well as a validated 89-item food frequency questionnaire (collected over one month). Principal component analysis was used to derive the dietary pattern. While central obesity was assessed using waist and/or hip circumference measurements, fasting blood sugar was used for IBG. A multivariable logistic regression model was fitted with an odds ratio, 95% confidence intervals, and p-values reported.

Results: A total of 501 adults (95.3%) were interviewed, with a mean age of 41 years (±12). Five major dietary patterns explaining 71% of the total variance were identified: “nutrient-dense foods”, “high fat and protein”, “processed foods”, “alcohol drinks”, and “cereal diets”. While 20.4% (17.0—24.2%) had IBG, 14.6% (11.8—17.9) were centrally obese, and 94.6 (92.3—96.3) had an increased risk for cardiovascular complications. Central obesity is associated with upper wealth status (AOR = 6.92; 2.91—16.5), physical inactivity (AOR = 21.1; 2.77—161.4), a diet high in nutrient-dense foods (AOR = 1.75; 0.75—4.06), processed foods (AOR = 1.41; 0.57— 3.48), and cereal diets (AOR = 4.06; 1.87—8.82). The risk of IBG was associated with upper wealth status (AOR = 2.36; 1.36—4.10), physical inactivity (AOR = 2.17; 0.91—5.18), upper tercile of nutrient-dense foods (AOR = 1.35; 0.62—2.93), fat and protein diet (AOR = 1.31; 0.66—2.62), and cereal diet consumption (AOR = 3.87; 1.66—9.02).

Conclusion: IBG and central obesity were prevalent and predicted by upper tercile consumption of nutrient-dense foods, high fat and protein diets, processed foods, and cereal diets, which could guide dietary interventions.

Key words: central obesity; dietary intake; dietary pattern; impaired blood glucose; prediabetes

# Major dietary patterns of community dwelling adults and their associations with impaired blood glucose and central obesity in Eastern Ethiopia: diet-disease exploration study

1. Introduction

Now a day the lifestyle characteristics of individuals, including dietary intake, change from time to time dramatically. At the same time, the occurrence of non-communicable diseases (NCDs) associated with impaired blood glucose (IBG) and obesity is rising (1). Hence, these are early indicators of increased risk of NCDs and their complications (2, 3). NCDs are responsible for the deaths of 41 million people (71%) each year, with low- and middle-income countries accounting for more than 85% of all deaths. Obesity and high blood glucose kill about 17.9 and 1.6 million people, respectively (4). The burden of NCDs has significantly increased, mainly attributable to dietary habit change, increasing obesity and poor lifestyles as a result of poor lifestyle practices (5). These are becoming major public health problems, especially in developing countries, accounting for an estimated 80% of all mortality (6).

Prediabetes or IBG, defined as fasting blood glucose (FBS) of 100-126 mg dl^-1^, is a precursor to established diabetes mellitus, which could be aggravated by insulin resistance due to abdominal obesity (7). In particular, central obesity, with a higher waist circumference (WC) and/or waist- to-hip circumference ratio (WHCR), is the sole cause of many NCDs related morbidity and mortality (8). Among the risk factors, dietary consumption is a long time exposure and one of the modifiable and preventable exposures for overt NCDs (3), which needs to be investigated systematically (9). Dietary risk factors were responsible for an estimated 11 million (10–12) deaths and 255 million (234–274) DALYs lost; including high sodium intake, (3 million deaths), low intake of whole grains (3 million deaths) and low intake of fruits ( 2 million deaths) (9).

Due to the methodological shortcomings of individual food or nutrient analysis for predicting health outcomes, dietary pattern analysis is a better approach to characterize dietary consumption. It is becoming a more practical approach to determine the role of diet in the prevention and treatment of chronic diseases (2), taking into account the confounding, interaction, and synergistic effects of nutrients in food that make causation difficult (10, 11). It

helps to evaluate the cumulative and interactive effects of each dietary compound in a food and to consider the complexity of the diet (10, 12).

Hence, dietary patterns could be more predictive for IBG and central obesity. Central obesity and IBG are the major risk factors that increase the risk of NCDs, including diabetes mellitus. NCDs are a public health problem in Ethiopia, where 4.2% are victims of diabetes mellitus, causing 70% of overall deaths (13). While an estimated up to 39% are centrally obese, 5% of overall mortality is due to IBG (14). In Ethiopia, an estimated 20.7% and 6.9% of adults are overweight and obese (15), where 35 to 38% of adults have central obesity (16). This huge burden of central obesity, is one main risk factor for established NCDs and all their complications (17, 18). For instance, an estimated 25% of IBG will progress to diabetes while 50% remain in an impaired state (19, 20) and increases cardiovascular mortality by 43% (21). The rate of progression to visible diseases is rapid, necessitating a targeted intervention to slow progression.

Given the huge role of diet in the development of central obesity and IBG, there is a need to investigate the link in the absence of such evidence in Ethiopia. The study area is characterized by physical access to diversified diet from agricultural productivity, industrial processing and imports. Studies conducted in other countries showed a clear link between dietary patterns and the risk of central obesity and IBD (22-25). However, evidence from other countries may not be applicable to our case, where dietary habits greatly vary even from region-to-region. In addition, studies conducted so far mainly focus on individual food consumptions (13, 16, 26, 27), which cannot be powerful predictors. This would help to design appropriate context-specific dietary strategies to decrease the risk of central obesity and IBG in Ethiopia. This study was to evaluate the association between major dietary patterns and central obesity and IBG among adults in Ethiopia.

# 2. Materials and Methods

## 2.1. Study Area and Period

This study was conducted in Dire Dawa and Harar, located in the eastern parts of Ethiopia. Harar is located 526 kilometers from the capital city, Addis Ababa. Dire Dawa has an estimated population of more than 0.5 million, while more than 0.3 million people reside in the Harari regions, where more than half of the population resides in urban areas. According to the 2010

Harari Region population projection, there are 250, 093 with 146, 913 living in the urban areas and 122, 942 are males, with a total household of 64, 334. These two regions have a multiethnic and diverse population with a heterogenous agricultural productivity for fruits, vegetables, sorghum, maize, wheat, and other cereals. Furthermore, being an industrial center for many manufacturing industries and a trade center closest to neighboring countries makes access to imported foods easier, in addition to domestic production. Hence, rice, white flour, macaroni, and other industrially processed foods are accessible, in addition to cereals and grains marketed from other regions of the country. The study was conducted from September to November, 2021. The study period was considered the fasting and feasting period to capture habitual Cereals, including teff, wheat, and legumes, were the most staple crops in the study areas. In addition, some processed foods, potatoes, and other fruits are the major foods consumed in the study area.

## 2.2. Study design and population

A community-based survey was conducted among 501 randomly selected adults (aged >= 18 years) from the two study sites available during the study period. Hence, the findings of this study would be generalized to all adults residing in Harar and Dire Dawa towns, Eastern Ethiopia. Adults who were pregnant, had abdominal swelling (ascites), or had detectable thoracic and lumbar spinal deformity were excluded because waist circumference (WC) measurements are biased. Also, clients with serious psychiatric disorders, which make them unable to give oriented responses and unable to communicate, were excluded. More importantly, adults with clinically diagnosed diabetes and/or known diabetes patients on treatment were also excluded from the study.

## 2.3. Sample size determination and sampling procedures

The minimum sample size for this study was estimated using a sample size formula for single proportion with ”P” as the prevalence of central obesity from the previous study at 24.4% (26), at a 95% confidence level, a “Z” critical value of 1.96, and a marginal error of “d” of 5%. The sample size became 145. The sample size for the association of dietary pattern with central obesity and IBG was calculated using the StatCalc module in Epi Info software version 7.0. After checking for many risk factors, the maximum sample size calculated was taken considering a prudent dietary pattern for predicting IBG (AOR = 2.26), abnormal fasting blood glucose level (OR = 2.26), percentage of IBG among those with a healthy dietary pattern (24%) (28), power of

80%, and a ratio of exposed and unexposed as one. Thus, taking dietary pattern as a risk factor for IBG and central obesity, the sample size became 504. After adding a non-response rate of 5%, the final minimum sample for this study was 526.

Stratified random sampling combined with simple and systematic random sampling was employed to select study participants. The total sample size was proportionally allocated to the two study sites (Dire Dawa and Harar) and to the smaller study units consecutively. A random sample of four districts in Harar and four kebeles (fourth administrative division level in Ethiopia) were randomly selected, where households were selected using systematic random sampling at a skipping interval. The sample interval (K) was calculated by dividing the total number of households by the allocated sample size for each kebele. Then, a random sample of adults residing in the selected household was selected and interviewed. The first household was selected randomly, and adults residing in randomly selected households were included in the study.

## 2.4. Data collection

Data were collected through a face-to-face interview using a set of pretested and structured questionnaires, as well as standardized anthropometric measurements and FBS measurement. The tool contains socio-demographic variables, lifestyle-related questions, and a validated semi- quantitative Food Frequency Questionnaire (FFQ) to capture the dietary intake of adults over the last one month. Data was collected by trained health care workers and graduating health students through face-to-face interviews, measurements, and laboratory analysis. Data collectors got the data by interviewing the study subject and probing for possible missed food consumed during the specific period of time.

The 16-item validated General Physical Activity Questionnaire (GPAQ) tool developed by WHO for physical activity surveillance was employed to assess the level of physical activity through face-to-face interviews. The activity level of the study participants was evaluated according to the standard WHO total physical activity calculation guide, and the level of total physical activity was categorized as physically active or inactive as detailed in the previous section. After calculating the metabolic equivalent per week (MET/week), a value below 600 was used to indicate physical inactivity (29).

A modified semi-quantitative Helen Keller FFQ containing 89 food items with about nine food groups was used to collect the dietary intake of study subjects. The FFQ was asked for the food that the subject consumed over the past 1 month to capture reliable and habitual dietary consumption of the individual. We further contextualized the list of food items for the particular context by considering the local foods commonly produced and consumed (Supplementary file 1). The FFQ validated for the Ethiopian adults has been contextualized and pretested to capture the dietary intake of adults. A validation study showed that the contextualized tool was valid and reliable in measuring the dietary micro- and macronutrient intake of adults in Ethiopia (30). In pilot analysis, the FFQ tool we assed was found to be highly reliable with a Cronbach’s alpha value above 0.86, indicative of a reproducible tool (31).

For assessment of central obesity, WC and hip circumference (HC) measurements were taken by trained anthropometric measurers using a non-stretchable tape meter at the end of a gentle expiration. The clothing and jewelry of the clients will be kept minimal. The WC was measured at a point midway between the lowest rib and the iliac crest in a horizontal plane at around the umbilicus while the respondents were instructed to breath gently. The tape meter is not tightly secured to avoid pressure and bias in measurement. While the HC was measured at the maximum circumference of the pelvis over the great trochanters, Measurements were made at least twice, and the average of the two measurements was recorded.

The laboratory analysis for blood glucose measurement was done by qualified and trained health professionals. The blood glucose measurement was done using a 2 mL capillary blood sample collected from the finger tips, and analyzed using a simple yet valid glucometer machine and recorded as mg dl-1. A specific cut off point from 100 to 125 mg dl-1 was used to diagnose IBG or prediabetes.

## 2.5. Data quality assurance

A pair of trained data collectors were deployed to collect the data from study subjects, as anthropometry needs curious measurements. Training was given on appropriate interview techniques and anthropometric measurements on WC and HC. Anthropometric reliability was assessed on 10 study subjects during the pretest, and Intra- and interobserver variation were calculated. Data collectors with high inter and both intra-observer bias (technical error of measurement above 2%) were retrained and collected the data once it was in the acceptable

range (32). All standard measuring procedures and instruments were strictly followed during data collection. The standard operating procedure for blood sampling and glucose testing was followed accordingly. During data entry into EpiData, the data quality was maintained by making legal ranges, skipping patterns, appropriate coding, and careful data entry. A Piot study was conducted on the unselected study area in order to test and correct the necessary corrections. The FFQ is adapted and modified with the purpose of not missing relevant foods that are commonly consumed in the study area (33).

## 2.6. Data analysis

After checking for completeness and inconsistencies, the collected data was entered into EpiData Version 3.02 and exported to SPSS for analysis. All statistical analyses were carried out with Microsoft Excel 2016 and IBM SPSS Statistics for Windows, Version 20.0 (IBM Corp., Armonk, NY, USA). Descriptive statistics were computed for each variable, and the data was presented in statistical tables and graphs. The WHCR was calculated from WC and HC and checked for normality using the Kolmogorov-Smirnov test. Principal component analysis was conducted using household assets to derive the socioeconomic status (wealth index) of adults. All assumptions of PCA were checked using the Kaiser Mayer-Olkin (KMO) sampling adequacy, correlations, and covariance parameters. Based on the derived factor scores, wealth status was categorized into five quartiles, from the poorest to the wealthiest.

Dietary pattern analysis

Exploratory factor analysis using a principal component analysis was used to derive dietary patterns using the average frequency of 89-item food consumption in the past one month. The consumption frequency was recategorized into daily equivalents based on previous literature (34) and weighted daily consumption equivalents (Supplementary File 2). In addition, some closely related food items were summed up and recategorized into a single food item as needed. Bartlett’s test of sphericity and the KMO measure of sampling adequacy were used to verify the appropriateness of exploratory factor analysis. For the degree of intercorrelations between variables, we used a KMO value of greater than 0.5 and a p-value of less than 0.05 for the Bartlett test of sphericity. Orthogonally rotation using the varimax option was used to enhance the interpretability of dietary patterns (35). The principal components were retained based on the following criteria: factor eigenvalue, identification of a break point in the scree plot, the

proportion of variance explained, and interpretability of the dietary patterns (12, 36). A rotated factor loading matrix was used to assess the direction and strength of correlation between each food item and its components. We also checked for a complex association between one food items and multiple factors, where such factors were removed. The corresponding factor score for each dietary pattern was constructed by summing the observed daily frequency of consumption of individual food items weighted by the factor loading. Hence, a high score of a component indicates more frequent consumption of the foods constituting that particular dietary pattern. For further analysis, each factor score was ranked into three terciles as low, medium, and high frequency of consumption.

Dietary pattern with central obesity and IBG

A separate multivariable logistic regression model was fitted to evaluate the association between dietary pattern and central obesity and IBG. Both crude and adjusted odds ratios with 95% confidence intervals and/or p-values were computed. Multicollinearity was checked using the variance inflation factor (VIF) and/or the standard error estimates. The model fitness was checked using Hosmer and Lemeshow’s goodness of fit. In addition, variables with a p-value below 0.2 in bivariable logistic regression and biologically plausible risk factors for the outcomes were considered in the multivariable logistic models. Statistical significance was declared at a p-value below 0.05.

## 2.7. Variables of the study

While the dependent variables of this study were IBG and central obesity, the independent variables were sociodemographic variables (age, sex, income, occupation, and education), physical activity, family history of diabetes, dietary pattern, dietary intake of food groups, and habits of substance use. The outcome variable IBG was categorized as IBG when the FBS between 100 and 125 mg dl-1 (37). Central obesity was defined as having a waist circumference of 94 cm for men and 80 cm for women, or a WHCR of 0.90 in men and 0.85 in women (38).

## 2.8. Ethical approval

Ethical approval was obtained from Dire Dawa University, Institutional Research Review Board. The support letter was taken to the respective regional health office for cooperation. A written informed consent was obtained from each study participant after explaining the study purpose,

confidentiality, and procedures in detail. The study was conducted in accordance with the Declaration of Helsinki for Human Research (39). The data will be used for this research purpose only, and the data will be kept confidential. Respondents identified with central obesity and IBG got counseling, recommendation, and/or linkage to the health facilities. All the COVID-19 standard prevention precautions were implemented throughout the data collection process.

# 3. Results

## 3.1. Sociodemographic characteristics

A total of 501 study subjects were included in the current survey, with a response rate of 95.3%. More than half (66.7%) of the participants were females. The age of the respondents ranges from 18—64 years, with a mean age of 41 years (±12). Almost all (90%) of the respondents reside with their families. A total of 203 (40.5%) attended primary school, and 143 (28.5%) and 127

(25.3%) were housewives and government employees, respectively. About 188 (37.5%) and 44 (8.8%) were in the wealthier and wealthiest ranks regarding socioeconomic status. On the other hand, 105 (21%) and 100 (20%) of the respondents were from the poorest and poorer households, respectively (Table 1).

**Table 1.** sociodemographic characteristics of respondents for a dietary pattern study in Dire Dawa and Harar, eastern Ethiopia

| Variables | Categories | Frequency | Percent |
| --- | --- | --- | --- |
| Sex | Male | 182 | 33.3 |
|  | Female | 319 | 66.7 |
| Live with | With family | 450 | 90 |
|  | Friends | 28 | 5.6 |
|  | Lonely | 21 | 4.4 |
| Education | Illiterate | 24 | 4.6 |
|  | Primary education | 203 | 40.5 |
|  | Secondary education | 107 | 21.4 |
|  | College and above | 167 | 33.3 |
| Marital status | Married | 409 | 81.6 |
|  | Single | 71 | 14.2 |
|  | Widowed | 21 | 4.2 |
| Occupation | Daily laborer | 18 | 3.6 |
|  | Farmer | 9 | 1.8 |
|  | Government employee | 127 | 25.3 |
|  | Housewife | 143 | 28.5 |
|  | Merchant | 69 | 13.8 |
|  | Private employed | 61 | 12.2 |

|  | Others | 74 | 14.8 |
| --- | --- | --- | --- |
| Wealth index | Poorest | 105 | 21.0 |
|  | Poor | 100 | 20.0 |
|  | Medium | 64 | 12.8 |
|  | Wealthier | 188 | 37.5 |
|  | Wealthiest | 44 | 8.8 |

3.2. Physical activity and substance use related factors

Regarding the physical activity of adults, more than three-fourths (383, 76.8%) of them were reported to be physically inactive with a median metabolic equivalent of 240 MET (IQR: 0-500 MET). While only 55 (11%) had a regular physical activity schedule, 85 (27.9%) had a family history of diabetes mellitus. Approximately 304 (60.7%) reported chewing khat, 101 (20.2%) reported drinking alcohol, and 24 (4.8%) reported chewing khat and smoking cigarettes.

3.3. Dietary patterns of adults

A total of 465 (98%) of adults had a usual three-times-a-day meal, while 478 (98.4%) were reported to have a regular meal. Almost all (99.4%) reported using liquid oils for cooking. About

95 (19%) and 48 (9.6%) reported having regular sweet and soda drinks and fatty foods, respectively. In terms of fruit and vegetable consumption, only 96 (19.2%) reported regular fruit consumption and 140 (27.9%) reported vegetable consumption (at least >= 6 times per week).

The consumption frequency was recategorized into daily equivalents based on previous literature and weighted daily consumption equivalents. In addition, some closely related food items were summed up and categorized into a single food item as needed. An exploratory factor analysis using the principal component analysis method was conducted. Food items with a KMO value of below 0.30 were removed iteratively. All the assumptions of PCA were checked. The overall sample adequacy was evaluated using KMO, where the p-value was 0.91. Similarly, the presence of interitem correlation was checked using the Bartlett test of sphericity (p-value 0.0001), indicating the presence of a statistically significant correlation. Furthermore, items with a communality below 0.30 were removed step-by-step. The final food items to be included were determined by the following criteria: interpretability of the factors, improving the total variance explained, absence of complex structure evidenced by rotation component matrix, eigen value, and scree plots (**Supplementary File 3)**.

Five dietary patterns explaining 71% of the total variance were identified; “Animal source foods, fruits, and vegetables-nutrient dense foods”, “high fat and protein foods”, “processed foods”, “traditional alcoholic drinks”, and “cereal foods”. A total of sixteen, nine, seven, four and four food items constitute the first, second, third, fourth, and fifth dietary patterns as indicted in **Supplementary File 3**. Nutrient dense foods composed of animal source foods, fruits, and vegetables account for the major part of the variance (42%), followed by high fat and protein foods (11.2%). For further analysis, the standard score of each dietary pattern was categorized into three terciles, where the lower score indicates less frequent consumption of foods and is assigned the lowest tercile. Furthermore, traditional alcoholic drinks were retained as they are relevant to central obesity and IBG (**Table 2****)**.

**Table 2**. Total variance explained and summary statistics for factor scores for identified dietary patterns in Ethiopia

| S.no | Dietary  patterns | Label | Variance  explained | Median  (Q_2_) | Q_1_ | Q_3_ |
| --- | --- | --- | --- | --- | --- | --- |
| 1 | DP_1 | Animal source foods, | 42% | -0.250 | -0.358 | -0.180 |
|  |  | fruits, and vegetables- |  |  |  |  |
|  |  | Nutrient dense foods |  |  |  |  |
| 2 | DP_2 | High fat and protein | 11.2% | -0.036 | -0.241 | 0.079 |
|  |  | foods |  |  |  |  |
| 3 | DP_3 | Processed foods | 9.0% | -0.460 | -0.762 | 0.667 |
| 4 | DP_4 | Traditional alcoholic | 5.0% | -0.146 | -0.176 | -0.056 |
|  |  | drinks |  |  |  |  |
| 5 | DP_5 | cereal foods | 3.83% | -0.177 | -0.265 | 0.087 |
|  | Total |  | 71.0% | - | - | - |

3.4. Magnitude of central obesity and impaired blood glucose

As clearly shown in Figure 1 below, an estimated 20.4% (95% CI: 17.0—24.2%) of the subjects had impaired blood glucose or prediabetes. While 14.6% (95% CI: 11.8—17.9%) of adults had central obesity based on the sex-specific WC cutoff points, 94.6 (95% CI: 92.3—96.3) of them had a WHCR, which makes them at risk for any cardiovascular complications (Figure 1). When disaggregated by sex, females had a higher rate of central obesity by WHCR (98% vs. 86%) and WC (17.2% vs. 9.9%). Moreover, the prevalence of IBGG was higher among males (22.5%)

compared to females (17.5%). The mean blood glucose level was	(50). Similarly,

180.1 mg dl^-1^

the mean and standard deviation for WC and WHCR were 66.7 (13.4) and 0.96 (0.46).

**Figure 1.** The magnitude of impaired blood glucose, central obesity (based on WC cutoff points of 80 and 94 cm for females and males, a WHC of 0.85 and 0.90 for females and males, b) among adults residing in Dire Dawa and Harar, Eastern Ethiopia.

3.5. Factors associated with central obesity and impaired blood glucose

An exploratory logistic regression was carried out to identify the association between sociodemographic factors and dietary patterns with IBG and central obesity among adults, as detailed in Table 3. Increasing age is associated with an increased risk of central obesity and IBG. While males (COR = 1.24; 95% CI: 0.79—1.93) had a 24% higher risk of IBG compared to females, females (COR = 1.86; 1.05—3.30) had a significantly higher risk of central obesity. The magnitude of IBG (COR = 2.18; 95% CI: 1.34—3.53) and central obesity (COR = 9.65; 95% CI: 4.29—21.7) was 2.1 and 9.65 folds higher in the wealthiest versus poorest families, respectively. Similarly, housewives had 38% increased odds of IBG (COR = 1.38; 95% CI: 0.84—2.28) and central obesity (COR = 2.33; 95% CI: 1.34—4.04) compared to adults engaged in private work. The risk of central obesity was found to be high among those with irregular meals (COR = 1.68; 95% CI: 0.60—4.66), physically inactive (COR = 1.54; 95% CI: 0.95—2.503), and habits of

sweet food consumption (COR = 1.63; 95% CI: 0.91—2.91) (Table 3).

**Table 3.** Bivariable logistic regression output for determinants of IBG and central obesity among adults from Dire Dawa and Harar, Eastern Ethiopia.

| Factors | Categories | IBG (pre  diabetes) | | COR (95% CI) | Central obesity  (WC) | | COR (95% CI) |
| --- | --- | --- | --- | --- | --- | --- | --- |
|  |  | IBG | No  IBG |  | Yes | No |  |
| Age in years | | - | - | 1.01 (0.99-1.03) | - | - | 1.03 (1.01-1.1) |
| Sex | Female | 61 | 258 | 1 | 55 | 264 | 1.86 (1.05-3.30)  * |
|  | Male | 41 | 141 | 1.24(0.79-1.93) | 18 | 164 | 1 |
| Wealth index | Poor | 30 | 175 | 1 | 7 | 198 | 1 |
|  | Medium | 9 | 55 | 0.96 (0.43-2.13) | 7 | 57 | 3.47(1.17-10.3) * |
|  | Wealthier | 63 | 169 | 2.18(1.34-3.53) * | 59 | 173 | 9.65(4.29-21.7) * |
| Occupation | Housewife | 35 | 106 | 1.38(.84-2.28) | 34 | 107 | 2.33(1.34-4.04) * |
|  | Government | 22 | 105 | 0.88(.50-1.54) | 11 | 116 | 0.69(0.33-1.45) |

|  | Private | 45 | 188 |  | 28 | 205 | 1 |
| --- | --- | --- | --- | --- | --- | --- | --- |
| Regular  meals | No | 1 | 23 | 1 | 5 | 18 | 1.68(0.60-4.66) |
|  | Yes | 102 | 375 |  | 68 | 410 | 1 |
| Physical  activity | Physically  active | 31 | 88 | 1.54(0.95-2.50) | 1 | 118 | 1 |
|  | Physically  inactive | 71 | 311 | 1 | 72 | 310 | 1.54(0.95-2.503) |
| Sweet and  soda drinks | No | 75 | 331 | 1 | 54 | 352 | 1 |
|  | Yes | 27 | 68 | 1.75(1.05-2.92) * | 19 | 76 | 1.63(0.91-2.91) |
| Regular  physical activity | No | 94 | 352 | 1.57(0.72-3.43) | 64 | 382 | 0.86(0.40-1.83 |
|  | Yes | 8 | 47 | 1 | 9 | 46 |  |
| Nutrient  dense foods (DP-1) | Lowest | 45 | 119 | 1.76(1.05-2.95) * | 27 | 137 | 1 |
|  | Middle | 26 | 136 | 0.89(0.50-1.57) | 6 | 156 | 0.20(0.08-0.49) |
|  | Highest | 31 | 144 | 1 | 40 | 135 | 1.50(0.87-2.59) |
| High fat and  protein diet (DP-2) | Lowest | 41 | 126 | 1.70(1.0-2.92) | 33 | 134 | 1.63(0.91-2.94) |
|  | Middle | 34 | 132 | 1.35(0.77-2.35) | 18 | 148 | 0.81(0.42-1.57) |
|  | Highest | 27 | 141 | 1 | 22 | 146 | 1 |
| Processed  foods (DP-3) | Lowest | 18 | 148 | 1 | 20 | 146 | 1 |
|  | Middle | 32 | 137 | 1.92(1.03-3.58) * | 21 | 148 | 1.04(0.54-1.99) |
|  | Highest | 52 | 114 | 3.75 (2.08-6.76) * | 32 | 134 | 1.74(0.95-3.20) |
| Alcoholic  drinks (DP- 4) | Lowest | 34 | 133 | 1 | 24 | 142 | 1 |
|  | Middle | 31 | 132 | 1.37(0.82-2.28) | 13 | 150 | .51(0.25-1.05) * |
|  | Highest | 37 | 134 | 0.68(0.38-1.20) | 36 | 136 | 1.57 (0.89-2.76) |
| Cereal diets  (DP-5) | Lowest | 34 | 133 | 1 | 13 | 154 | 1 |
|  | Middle | 31 | 132 | .092(0.53-1.58) | 17 | 146 | 1.38 (0.65-2.94) |
|  | Highest | 37 | 134 | 1.08(0.64-1.82) | 43 | 128 | 3.98(2.05-7.72) * |

*Note: * statistically significant association at p-value <0.05.*

Dietary patterns were found to be associated with the risk of central obesity and IBG among adults. Adults who consumed less nutrient dense food (COR = 1.76; 95% CI: 1.05—2.95) and more fat and protein-rich foods (COR = 1.35; 95% CI: 0.77—2.35), for example, had a 1.76- and 1.35-times higher risk of IBG, respectively. More frequent consumption of processed foods (COR = 3.75; 95% CI: 2.08—6.76) and medium consumption of traditional alcoholic drinks (COR = 1.37; 95% CI: 0.82—2.28) were significant predictors of IBD. However, optimal consumption of a cereal diet was protective against IBG. Furthermore, eating nutrient—dense foods (COR = 1.50; 95% CI: 0.87—2.59) and highly processed foods (COR = 1.74; 95% CI: 0.95—3.20) increased the likelihood of central obesity by 50% and 74%, respectively. Frequent consumption of traditional alcoholic drinks (COR = 1.57; 95% CI: 0.89—2.76) and consumption of cereal diets (COR = 3.98 (2.05—7.72)) was shown to increase the risk of central obesity by

1.57 and 3.58—folds. However, in a dietary pattern characterized by fat and protein-rich foods, the risk for both central obesity (COR = 1.70; 95% CI: 1.0—2.92) and IBG (COR = 1.63; 95% CI: 0.91—2.94) was higher among those with a lower tercile (Table 3).

A separate multivariable logistic model was fitted in a step-wise backward regression approach for central obesity and IBG. Sex, wealth status, physical activity level, and dietary patterns 1, 3, and 5 were found to be associated with central obesity among adults. Being physically inactive (600 MET) (AOR = 21.1; 95% CI: 2.77—161.4), from a higher socioeconomic class (6.92 (2.91—16.5), and females (AOR = 1.67 (0.82—3.40) were positively associated with a higher risk of central obesity. More importantly, those who consumed more nutrient-dense foods (1.75; 95% CI: 0.75—4.06), processed foods (AOR = 1.41 (0.57—3.48), and cereal diets (AOR = 4.06; 95% CI: 1.87—8.82) had a higher risk of central obesity. An increasing dose—response relationship was observed where the risk is higher for more frequent consumption (Table 4).

**Table 4.** Adjusted association between dietary patterns and central obesity among adults from Dire Dawa and Harar, Eastern Ethiopia.

| Factors | Categories | Central obesity  (WC) | | AOR (95% CI) | p-value |
| --- | --- | --- | --- | --- | --- |
|  |  | Yes | No |  |  |
| Sex | Female | 55 | 264 | 1.67(0.82-3.40) | 0.157 |
|  | Male | 18 | 164 | 1 |  |
| Wealth index | Poor | 7 | 198 | 1 |  |
|  | Medium | 7 | 57 | 2.56(0.80-8.25* | 0.115 |
|  | Wealthier | 59 | 173 | 6.92(2.91-16.5) | 0.001** |
| Physical activity | Physically active | 1 | 118 | 1 |  |
|  | Physically inactive | 72 | 310 | 21.1(2.77-161.4) | 0.003** |
| Nutrient dense foods (DP-1) | Lowest | 27 | 137 | 1 |  |
|  | Middle | 6 | 156 | 0.28(0.09-0.87) | 0.028* |
|  | Highest | 40 | 135 | 1.75(0.75-4.06) | .193 |
| Processed foods (DP-3) | Lowest | 20 | 146 | 1 |  |
|  | Middle | 21 | 148 | 1.59(0.73-3.46) | 0.246 |
|  | Highest | 32 | 134 | 1.41(0.57-3.48) | 0.456 |
| Cereal diets (DP-5) | Lowest | 13 | 154 | 1 |  |
|  | Middle | 17 | 146 | 1.51(0.66-3.45) | 0.325 |
|  | Highest | 43 | 128 | 4.06(1.87-8.82) * | 0.0001** |

Note: statistically significant factors associated with central obesity at p-value <0.05* and 0.001^**^

A multivariable logistic regression output was fitted using Hosmer and Lemeshow’s goodness of fit (P-value = 0.847), indicating a fit model. Those from wealthier families (AOR = 2.36; 95% CI: 1.36—4.10), those without regular physical activity (AOR = 2.17; 95% CI: 0.91—5.18) and those with sweet food consumption habits (AOR = 2.46; 95% CI: 1.30—4.66) had a 2.36—,

2.17—, and 2.46—times increased risk of having IBG level compared to counterparts. Among the identified dietary patterns, lower consumption of nutrient-dense foods (AOR = 1.35; 0.62— 2.93), higher fat and protein diets (AOR = 1.31; 95% CI: 0.66—2.62) and higher cereal diet consumption (AOR = 3.87; 95% CI: 1.66—9.02) were important dietary determinants of IBG level. On the other hand, the risk of IBG was 60% higher among those who were physically active (AOR = 1.60; 95% CI: 0.90–2.85) (Table 5).

**Table 5**. Multivariable logistic regression showing the risk factors of IBG among adults residing in Dire Dawa and Harar, Eastern Ethiopia

| Factors | Categories | IBG (pre  diabetes) | | COR (95% CI) | P-value |
| --- | --- | --- | --- | --- | --- |
|  |  | IBG | No IBG |  |  |
| Wealth index | Poor | 30 | 175 | 1 |  |
|  | Medium | 9 | 55 | 1.29(0.54-3.04) | 0.567 |
|  | Wealthier | 63 | 169 | 2.36(1.36-4.10) | 0.002* |
| Physical activity | Physically active | 31 | 88 | 1.60(0.90-2.85) | 0.107 |
|  | Physically inactive | 71 | 311 | 1 |  |
| Sweet and soda drinks | No | 75 | 331 | 1 |  |
|  | Yes | 27 | 68 | 2.46(1.30-4.66) | 0.006* |
| Regular physical activity | No | 94 | 352 | 2.17(0.91-5.18) | 0.083 |
|  | Yes | 8 | 47 | 1 |  |
| Nutrient dense foods (DP-1) | Lowest | 45 | 119 | 1.35(0.62-2.93) | 0.490 |
|  | Middle | 26 | 136 | 1.27(0.65-2.5) |  |
|  | Highest | 31 | 144 | 1 |  |
| High fat and protein diet (DP-2) | Lowest | 41 | 126 | 1.42(0.75-2.71) | 0.285 |
|  | Middle | 34 | 132 | 1.31(0.66-2.62) | 0.443* |
|  | Highest | 27 | 141 | 1 |  |
| Processed foods (DP-3) | Lowest | 18 | 148 | 1 |  |
|  | Middle | 32 | 137 | 1.97(1.01-3.86) | 0.048* |
|  | Highest | 52 | 114 | 3.87(1.66-9.02) | 0.002** |

*Note: statistica lly significa nt factors associat ed	with central obesity at	p-*

*value*

*<0.05^*^*

*and 0.001^**^*

# 4. Discussion

The burden of NCDs is rising at an alarming rate, accounting for more than 70% of adult mortality (40). Prediabetes and central obesity associated with metabolic syndrome are the most important risk factors for the development of overt NCDs (41). These diseases are in turn dependent on the lifestyle and dietary consumption habits of individuals, which could be modified for a better health condition (42). Hence, we conducted the current study to identify the role of dietary patterns in the development of prediabetes (IBG) and central obesity among adults.

In this study, we found that 20.4% had IBG, 14.6% had central obesity, and 94% had an increased risk for cardiovascular complications based on the sex-specific WHCR cutoff point. This indicates IBG is a common public health problem, especially among females, and it may be cross linked with a comparable occurrence of central obesity (abdominal obesity) based on WC cutoff points. Evidence from Ethiopia also showed that the prevalence of IBG ranges from 8%

(43) to 12% (44). It should be emphasized that central obesity is a main risk factor for insulin resistance and the development of IBG. Since 85% of NCD deaths occur in low- and middle income countries (4), multilateral dietary and lifestyle interventions are required to halt central obesity and IBG. This will be needed to decrease the incidence of metabolic risk factors before an overt metabolic disease (cardiovascular or diabetes) happens. A higher prevalence of central obesity (39%) was reported in southern Ethiopia (45).

In the present study, being female, higher socioeconomic class (AOR = 6.92; 95% CI: 2.91— 16.5), and physical inactivity (AOR = 21.1; 95% CI: 2.77—161.4) were found to be important predictors of central obesity. A similar association was also identified with IBG, except that being physically active is associated with a higher risk of IBG. This was also supported by findings from Ethiopia, where females had a 9-times (AOR = 9.62; 95% CI: 4.84—19.12) (16) and 12-times (AOR = 12.93, 95% CI: 6.74—24.79) higher risk of obesity than males. A similar association was also reported for socioeconomic class (46), where poor lifestyles and access to and consumption of unhealthy foods are more common. In addition, physical activity and having regular physical activity had a crucial for maintaining energy balance. A study indicated that adults who were physically active were lower risk of central obesity (AOR = 0.87: p-value 0.174), emphasizing the need for regular physical activity (46).

On the other hand, the role of non-exercise energy expenditures like working, walking, and others should be encouraged as these are very crucial for maintaining optimal fat storage (47, 48). However, the negative association with IBG could be explained by the fact that those with some risk of cardiovascular complications might have poor blood sugar levels. This does not invalidate the positive role of physical activity for optimal weight and blood glucose. Rather, it reflects the characteristics of the study subjects.

Although there is evidence for the link between some dietary consumption habits and central obesity and IBG (49), more concrete and context-specific evidence using a more robust approach is needed to guide obesity and diabetes prevention strategies (50). Due to the complex

and multicollinear nature of the human diet and the limitations of individual food or nutrient analysis for predicting a particular health outcome, dietary pattern analysis using a more robust statistical approach is becoming a novel approach for better recommendations (12, 36). Hence, we derived the dietary patterns and its association with the development of central obesity and IBG.

Those who reported consuming processed foods (AOR = 1.41; 95% CI: 0.57—3.48) and cereal

diets (AOR = 4.06; 95% CI: 1.87—8.82) were a major risk factor for the development of central

obesity. Other studies indicated that the top tercile of western pattern consumption (OR = 1.82;

95% CI: 1.16–2.87) increased the risk for obesity while a prudent pattern (AOR = 0.62; 95% CI:

0.40–0.96) reduced their risk for central obesity (51). On the other hand, those with a low

consumption of nutrient dense foods (animal sources, fruits, and vegetables) (AOR = 1.35; 95%

CI: 0.62—2.93) had a 35% increased risk of IBG compared to those with a higher tercile. This could be explained by undernourished adults' metabolic limitations and an increased risk of insulin insufficiency for glucose metabolism (52).

Other studies also showed that dietary patterns were highly correlated with abnormal blood glucose status (r = 0.8) (53). A review of the evidence also found that healthy, traditional, and Mediterranean diets rich in sea foods, fruits, and vegetables were effective in preventing IBG (54). Similarly, higher adherence to the unhealthy dietary pattern (saturated fats and meat) was more likely to cause insulin resistance and IBG as a result (49). On the contrary, a vegetarian dietary pattern reduced the risk of IBG (p-value below 0.05) (28). For instance, a study showed that a higher tercile of the western dietary pattern was associated with an increased risk of obesity (AOR = 1.80; 95% CI: 1.15–2.81) compared to a lower tercile (55).

The association between lower tercile consumption of nutrient-dense foods with IBG could be related to the issue of food security and the risk of having unhealthy dietary patterns. For example, a study found that food insecurity could lead to unhealthy dietary patterns and ultimately increase the risk of central obesity (56). The current study depicted the role of dietary pattern in the development of central obesity and prediabetes. These could help to design and indicate an effective dietary strategy to prevent obesity and its associated consequences (57). However, in assessing food consumption, the potential for recall bias and respondent bias could

not be excluded, which might affect the derived dietary patterns. Moreover, the current study did

not collect quantitative data on daily intake to quantify the nutrient intake beyond food consumption.

# 5. Conclusion and

recommendations

Overall, the occurrence of central obesity, IBG, and increased risk of cardiovascular complications were prevalent among adults. Dietary patterns characterizing the overall diet of individuals are more predictive of the outcomes, which could be replicated for other relevant diet-related diseases. Higher tercile consumption of nutrient dense foods, high fat and protein diets, processed foods, and cereal diets are important risk factors for central obesity and IBG. Optimal consumption of a diversified diet from animal sources, fruits, vegetables, cereals, and protein-rich foods could play a great role in preventing the occurrence of metabolic syndromes, while consumption of processed foods and alcoholic drinks should be kept to a minimum.

### Declarations

**Ethical approval and consent to participate**

Ethical approval was obtained from the Institutional Research Ethical Review Board of Dire Dawa University. Written informed assent was obtained from all adults This research was conducted in accordance with the Helsinki Declaration for protecting human study subjects.

**Consent to publish** Not applicable **Acknowledgment**

We are grateful to Dire Dawa University and Dire Dawa Health Bureau for their collaborations and support for the successful completion of the study. Our gratitude also goes to the respective health bureaus, respondents, data collectors, and supervisors for their sincere help and collaboration for the successful completion of the research.

Author contribution

AO- participated in conceptualization, fund acquisition, design, data curation, resource, project administration, data analysis, writing a report, and reviewing and approving the manuscript. AA, AH, and BA: participated in conceptualization, validation, supervision, methodology, data

preparation, data visualization, and formal data analysis. In addition, AO substantially contributed to writing the, drafting the manuscript, critically reviewing, and editing the drafted manuscript. All authors reviewed and approved the final manuscript and decided on the journal to submit to.

### Data Availiability

All relevant data are included within the manuscript and its supporting Information files.

### Funding

This study was funded by Dire Dawa University, under annual competitive research grant. The funders had no role in study design, data collection and analysis, the decision to publish, or the preparation of the manuscript.

### Competing interests

The authors have declared that no competing interests exist.

### Abbreviations:

CI—Confidence Interval; DP— Dietary Patterns; FFQ—Food Frequency Questionnaire; GPAQ—General Physical Activity Questionnaire; IBG; HC—Hip Circumference; Impaired Blood Glucose; K—Sampling Interval; METS—Metabolic Equivalents; PCA—Principal Component Analysis; NCDs—Non communicable Diseases; SD—Standard Deviations; VIF— Variance Inflation Factor; WC—Waist Circumference; WHCR—Waist—to—Hip Circumference Ratio; WHO —World Health Organization

# 6. References

1. LNeuhouser M. The Importance of Healthy Dietary Patterns in Chronic Disease Prevention. *Nutrition Research* (2019) 70(Commentary):3-6.
2. Tapsell LC, Neale EP, Probst Y. Dietary Patterns and Cardiovascular Disease: Insights and	Challenges	for	Considering	Food	Groups	and	Nutrient	Sources.	(2019). [Https://Doi.Org/10.1007/S11883-019-0770-1.](https://doi.org/10.1007/S11883-019-0770-1) *Curr Atheroscler Rep* (2019) 21(9):1-8.
3. Ojo O. Nutrition and Chronic Conditions. *Nutrients* (2019) 11(2):459.
4. World Health Organisation (WHO). Noncommunicable Diseases Geneva, Switherland(2018) [cited 2021 Jan 27].
5. Gouda HN, Charlson F, Sorsdahl K, Ahmadzada S, Ferrari AJ, Erskine H, et al. Burden of Non-Communicable Diseases in Sub-Saharan Africa, 1990–2017: Results from the Global Burden of Disease Study 2017. *The lancet Global Health* (2019) 7(10):1375-E87.
6. World Health Organization (WHO). *Global Status Report on Noncommunicable Diseases 2010*. Geneva: World Health Organization (2011). p. 162.
7. Santaguida P, Balion C, Hunt D, Morrison K, Gerstein H, P Raina LB, et al. Diagnosis, Prognosis, and Treatment of Impaired Glucose Tolerance and Impaired Fasting Glucose: Summary. *AHRQ Evidence Report Summaries* (2005).
8. Dhawan D, Sharma S. Abdominal Obesity, Adipokines and Non-Communicable Diseases. *The Journal of steroid biochemistry and molecular biology* (2020) 203:105737.
9. GBD 2017 Diet Collaborators. Health Effects of Dietary Risks in 195 Countries, 1990– 2017: A Systematic Analysis for the Global Burden of Disease Study 2017. *The Lancet* (2019) 393(10184):1958-72.
10. Aleksandrova K, Giuseppe Rd. Diet, Healthy Aging, and Cognitive Function. *Analysis in Nutrition Research*. Springer (2019).
11. Abdi H, Williams LJ. Principal Component Analysis. *Wiley interdisciplinary reviews: computational statistics* (2010) 2(4):433-59.
12. Agnoli C, Krogh V. Dietary Pattern Analysis. *Analysis in Nutrition Research*. Springer (2019).
13. Abebe SM, Andargie G, Shimeka A, Alemu K, Kebede Y, Wubeshet M, et al. The Prevalence of Non-Communicable Diseases in Northwest Ethiopia: Survey of Dabat Health and Demographic Surveillance System *BMJ Open* (2017) 7(10):1-7.
14. Misganaw A, Mariam DH, Ali A, Araya T. Epidemiology of Major Non-Communicable Diseases in Ethiopia: A Systematic Review. *J Health Popul Nutr* (2014) 32 (1):1-13.
15. Kassie AM, Abate BB, Kassaw MW. Prevalence of Overweight/Obesity among the Adult Population in Ethiopia: A Systematic Review and Meta-Analysis. *BMJ Open* (2020) 10(8):e039200. doi: 10.1136/bmjopen-2020-039200.
16. Molla MD, Wolde HF, Atnafu A. Magnitude of Central Obesity and Its Associated Factors among Adults in Urban Areas of Northwest Ethiopia. *Diabetes, Metabolic Syndrome and Obesity: Targets and Therapy* (2020) 13:4169-78.
17. Chooi YC, Ding C, Magkos F. The Epidemiology of Obesity. *Metabolism* (2019) 92:6- 10. doi: <https://doi.org/10.1016/j.metabol.2018.09.005>.
18. Bastien M, Poirier P, Lemieux I, Després Jp. Overview of Epidemiology and Contribution of Obesity to Cardiovascular Disease. Prog Cardiovasc Dis. 2014;56:369–81.
19. Gabir, Mm, Hanson, Rl, Dabelea, D. The 1997 American Diabetes Association and 1999 World Health Organization Criteria for Hyperglycemia in the Diagnosis and Prediction of Diabetes. Diabetes Care 2000; 23: 1108–1112.
20. Shaw, Je, Zimmet, Pz, De Courten, M. Impaired Fasting Glucose or Impaired Glucose Tolerance. What Best Predicts Future Diabetes in Mauritius? Diabetes Care 1999; 22: 399–402.
21. Forouhi NG, Balkau B, Borch-Johnsen K, Dekker J, Glumer C, Qiao Q, et al. The Threshold for Diagnosing Impaired Fasting Glucose: A Position Statement by the European Diabetes Epidemiology Group. *Diabetologia* (2006) 49(5):822-7. doi: 10.1007/s00125-006- 0189-4.
22. Hosseini-Esfahani F, Koochakpoor G, Daneshpour MS, Sedaghati-khayat B, Mirmiran P, Azizi F. Mediterranean Dietary Pattern Adherence Modify the Association between Fto Genetic Variations and Obesity Phenotypes. *Nutrients* (2017) 9(10):1064.
23. L.Strate L, R.Keeley B, YinCao, KanaWu, L.Giovannucci E, T.Chan A. Western Dietary Pattern Increases, and Prudent Dietary Pattern Decreases, Risk of Incident Diverticulitis in a Prospective Cohort Study. *Gastroenterology* (2017) 152(5):1023-30 e2.
24. Li M, Shi Z. Dietary Pattern During 1991–2011 and Its Association with Cardio Metabolic Risks in Chinese Adults: The China Health and Nutrition Survey. *Nutrients* (2017) 9(11):1218.
25. Mu M, Xu L-F, Hu D, Wu J, Bai M-J. Dietary Patterns and Overweight/Obesity: A Review Article. *Iran J Public Health* (2017) 46(7):869-76.
26. Tesfaye T, Zeleke TM, Alemu W, Argaw D, Bedane TK. Dietary Diversity and Physical Activity as a Risk Factors of Abdomenal Obesity among Adults in Dilla Town, Ethiopia. *PLOs One* (2020) 15(7):e0236671.
27. Ghachem A, Brochu M, Dionne IJ. Differential Clusters of Modifiable Risk Factors for Impaired Fasting Glucose Versus Impaired Glucose Tolerance in Adults 50 Years of Age and Older. *Ther Adv Chronic Dis* (2019) 10(eCollection). doi: <https://doi.org/10.1177/2040622319854239>.
28. Amini M, Esmaillzadeh A, Shafaizadeh S, Behrooz J, Zare M. Relationship between Major Dietary Pattern and Metabolic Syndrome among Individuals with Impaired Blood Glucose. *Nutrition* (2010) 26(6):986-92.
29. World Health organization. Global Physical Activity Questionnaire (Gpaq) Analysis Guide. Geneva: WHO, (2011) 1.
30. Regassa IF, Endris BS, Habtemariam E, Hassen HY, Ghebreyesus SH. Development and Validation of Food Frequency Questionnaire for Food and Nutrient Intakes of Adults in Butajira, Southern Ethiopia. *Journal of nutritional science* (2021) 10.
31. George D, Mallery P. Reliability Analysis. *Ibm Spss Statistics 25 Step by Step*. Routledge (2018). p. 249-60.
32. Perini TA, Oliveira GL OJ, Oliveira FP.,. Technical Error of Measurement in Anthropometry. ( 2005) 11:81-5.
33. Oumer A, Abraham M, Nuri A. Predictors of Major Dietary Patterns among Pregnant Women Attending Public Health Facilities in Eastern Ethiopia: A New Epidemiological Approach. *Frontiers in Nutrition* (2022) 9. doi: 10.3389/fnut.2022.855149.
34. Oumer A, Abraham M, Nuri A. Predictors of Major Dietary Patterns among Pregnant Women Attending Public Health Faility in Eastern Ethiopia: A New Epidemiological Approach. *Frontier in Nutrition* (2022) Accepted Manuscript.
35. Plaza-Díaz J, Molina-Montes E, Soto-Méndez MJ, Madrigal C, Hernández-Ruiz Á, Valero T, et al. Clustering of Dietary Patterns and Lifestyles among Spanish Children in the Esnupi Study †. *Nutrients* (2020) 12(9):2536.
36. Hu FB. Dietary Pattern Analysis: A New Direction in Nutritional Epidemiology. *Current opinion in lipidology* (2002) 13(1):3-9.
37. Genuth S, Alberti KG, Bennett P, Buse J, Defronzo R, Hahn R, et al. Expert Committee on the Diagnosis and Classification of Diabetes Mellitus: Follow-up Report on the Diagnosis of Diabetes Mellitus. Diabetes Care 26:3160–3167. (2003).
38. Ashwell M, Gibson S. Waist to Height Ratio Is a Simple and Effective Obesity Screening Tool for Cardiovascular Risk Factors: Analysis of Data from the British National Diet and Nutrition Survey of Adults Aged 19–64 Years. *Obesity facts* (2009) 2(2):97-103.
39. Association GAotWM. World Medical Association Declaration of Helsinki: Ethical Principles for Medical Research Involving Human Subjects. *Am J Dent* (2014) 81(3):14-8.
40. Ethipian Public health institute (EPHI). *Steps Survey on Risk Factors for Non Communicable Diseases and Prevlence of Selected Ncds Ethiopia.* In: Nutrition, editor. Addis Ababa: EPHI (2016). p. 2-25.
41. Peters R, Ee N, Peters J, Beckett N, Booth A, Rockwood K, et al. Common Risk Factors for Major Noncommunicable Disease, a Systematic Overview of Reviews and Commentary: The Implied Potential for Targeted Risk Reduction. *Therapeutic advances in chronic disease* (2019) 10:2040622319880392.
42. Mudie K, Jin MM, Kendall L, Addo J, dos-Santos-Silva I, Quint J, et al. Non- Communicable Diseases in Sub-Saharan Africa: A Scoping Review of Large Cohort Studies. *Journal of global health* (2019) 9(2).
43. Tesfaye T, Shikur B, Shimels T, Firdu N. Prevalence and Factors Associated with Diabetes Mellitus and Impaired Fasting Glucose Level among Members of Federal Police Commission Residing in Addis Ababa, Ethiopia. *BMC Endocrine Disorders* (2016) 16(1):68. doi: 10.1186/s12902-016-0150-6.
44. Worede A, Alemu S, Gelaw YA, Abebe M. The Prevalence of Impaired Fasting Glucose and Undiagnosed Diabetes Mellitus and Associated Risk Factors among Adults Living in a Rural Koladiba Town, Northwest Ethiopia. *BMC Res Notes* (2017) 10(1):251.
45. Tekalegn Y, Solomon D, Sahiledengle B, Assefa T, Negash W, Tahir A, et al. Prevalence of Central Obesity and Its Associated Risk Factors among Adults in Southeast Ethiopia: A Community-Based Cross-Sectional Study. *PloS one* (2022) 17(8):e0265107.
46. Owolabi EO, Ter Goon D, Adeniyi OV. Central Obesity and Normal-Weight Central Obesity among Adults Attending Healthcare Facilities in Buffalo City Metropolitan Municipality, South Africa: A Cross-Sectional Study. *Journal of Health, Population and Nutrition* (2017) 36(1):54. doi: 10.1186/s41043-017-0133-x.
47. Herrmann SD, Willis EA, Honas JJ, Lee J, Washburn RA, Donnelly JE. Energy Intake, Nonexercise Physical Activity, and Weight Loss in Responders and Nonresponders: The Midwest Exercise Trial 2. *Obesity* (2015) 23(8):1539-49.
48. Melanson E. The Effect of Exercise on Non‐ Exercise Physical Activity and Sedentary Behavior in Adults. *Obesity Reviews* (2017) 18:40-9.
49. Mazidi M, Kengne AP, Mikhailidis DP, Toth PP, Ray KK, Banach M. Dietary Food Patterns and Glucose/Insulin Homeostasis: A Cross-Sectional Study Involving 24,182 Adult Americans. *Lipids in health and disease* (2017) 16(1):192. doi: 10.1186/s12944-017-0571-x.
50. Bekele TH, De Vries JJ, Trijsburg L, Feskens E, Covic N, Kennedy G, et al. Methodology for Developing and Evaluating Food-Based Dietary Guidelines and a Healthy Eating Index for Ethiopia: A Study Protocol. *BMJ Open* (2019) 9(7):e027846.
51. Paradis, Am., Godin, G., Pérusse, L. Et Al. Associations between Dietary Patterns and Obesity Phenotypes. Int J Obes 33, 1419–1426 (2009). [Https://Doi.Org/10.1038/Ijo.2009.179](https://doi.org/10.1038/Ijo.2009.179).
52. Barker DJ, Thornburg KL. Placental Programming of Chronic Diseases, Cancer and Lifespan: A Review. *Placenta* (2013) 34(10):841-5.
53. Hlaing HH, Liabsuetrakul T. Dietary Intake, Food Pattern, and Abnormal Blood Glucose Status of Middle-Aged Adults: A Cross-Sectional Community-Based Study in Myanmar. *Food Nutr Res* ( 2016) 60::1-8.
54. Maghsoudi Z, Azadbakht L. How Dietary Patterns Could Have a Role in Prevention, Progression, or Management of Diabetes Mellitus? Review on the Current Evidence. *J Res Med Sci* (2020) 17(7):694–709.
55. Zhang X, Li Y, Liu a, Zhang Q, Hu X, S. Et Al. (2012)Dietary Pattern and Its Association with the Prevalence of Obesity and Related Cardiometabolic Risk Factors among Chinese Childre Dietary Pattern and Its Association with the Prevalence of Obesity and Related Cardiometabolic Risk Factors among Chinese Children. Plos One 7(8): E43183. [Https://Doi.Org/10.1371/Journal.Pone.0043183](https://doi.org/10.1371/Journal.Pone.0043183).
56. Morales ME, Berkowitz SA. The Relationship between Food Insecurity, Dietary Patterns, and Obesity. *Current Nutrition Reports* (2016) 5(1):54-60. doi: 10.1007/s13668-016-0153-y.
57. Seifu CN, Fahey PP, Hailemariam TG, Frost SA, Atlantis E. Dietary Patterns Associated with Obesity Outcomes in Adults: An Umbrella Review of Systematic Reviews. *Public Health Nutrition* (2021) 24(18):6390-414.

[Click here to access/download;Figure;Figure 1.tiff](https://www.editorialmanager.com/pone/download.aspx?id=32022452&amp;guid=03530997-5b12-419c-b0b6-f2ea3786c648&amp;scheme=1)

[Click here to access/download](https://www.editorialmanager.com/pone/download.aspx?id=32022487&amp;guid=f58f2d47-9ccf-4dcc-b905-4e8bf2f57033&amp;scheme=1)

[**Supporting Information** S3 File.docx](https://www.editorialmanager.com/pone/download.aspx?id=32022487&amp;guid=f58f2d47-9ccf-4dcc-b905-4e8bf2f57033&amp;scheme=1)

[Click here to access/download](https://www.editorialmanager.com/pone/download.aspx?id=32022488&amp;guid=825846f1-900a-4fbd-b6ec-fef5309cc17c&amp;scheme=1)

[**Supporting Information** S1 File.docx](https://www.editorialmanager.com/pone/download.aspx?id=32022488&amp;guid=825846f1-900a-4fbd-b6ec-fef5309cc17c&amp;scheme=1)

[Click here to access/download](https://www.editorialmanager.com/pone/download.aspx?id=32022489&amp;guid=6769ed3b-21de-4eb5-a0e4-49db07ed460a&amp;scheme=1)

[**Supporting Information** S2 File.docx](https://www.editorialmanager.com/pone/download.aspx?id=32022489&amp;guid=6769ed3b-21de-4eb5-a0e4-49db07ed460a&amp;scheme=1)
