# Supplementary material for: Major dietary patterns of community dwelling adults and their associations with impaired blood glucose and central obesity in Eastern Ethiopia: Diet-disease epidemiological study
Source: PLoS One. 2023 Apr 20;18(4):e0283075. doi: 10.1371/journal.pone.0283075 (PMC10118135; doi:10.1371/journal.pone.0283075)
Supplement: Attachment — Submitted filename: Resonse to Reviewers.docx [file pone.0283075.s005.docx]

PONE-D-22-28499

*Major dietary patterns of community dwelling adults and their associations with impaired blood glucose and central obesity in Eastern Ethiopia: diet-disease exploration study*

**Dear editor and reviewers**

Thank you very much for having such detail and valuable comments on our work. We have checked your comments and suggestion in more detail and have included them in the revised submission. All the changes are showed in track changes. Hence, we found it that giving a detail response for each point is not necessary. Thus, we only responded to question which need clarification ad included the requested changes in the revised manuscript simply.

On the title, the comment was reasonable and we amended the title accordingly. We also amended the comments on the abstract as it refers to percent based on WHCR cutoff point. These finding is discussed in more detail in the discussion part.

We specified the study design as survey or cross-sectional study design and the study period as “September 10 to November 06”.

You declared the final sample size as 526 in the section of sample size determination. Why you did you decide on 501?

The estimated minimum sample size required for this study was 526. However, the achieved response rate was 95% where a total 25 respondents were non-responders. They were not volunteer to participate in the study. The difference is due to this.

A separate multivariable model was done for IBG and central obesity as this is shown in Table 4 and 5. This is also clearly indicated there.

The interpretation of the odds ratio had some errors and we accepted the comments from the reviewers. Hence, we thoroughly edited the suggestions in way that the Odds ratio could be interpreted accordingly. Hence, we interpreted it as increased occurrence or odds of IBG or central obesity as it is indicated in the revised version. The comments are well accepted.

Computing COR is not conclusive to determine determinants of IBG.

Here, we run both the crude and adjusted odds ratio. This could allow us to clearly indicate the statistical analysis and the reader in a very clear and reproducible way. Since we also clearly reported the adjusted association, this is very informative than the crude one. Hence, we made our conclusion and recommendations based on the adjusted associations. Presenting the crude one makes the paper more holistic and reproducible.

*This discussion about the association between dietary pattern and central obesity and IBG in this study is not adequate. In addition, it is not exhaustive. You need to compare with similar finding and identify similar and unique variables. If this study identifies unique variable you need to have possible justification.*

Here, as you mentioned the objective of the study is like that and we tried our best to present the discussion in a very clear and sequential manner. We started with IBG and central obesity, then their association with empirical dietary patterns. The major challenge was lack of such evidence in Ethiopia and Africa as well. That is why we mainly used gray literatures and fats to support and explain our results. But we have tried our best to modify the discussion as per the reviewers’ suggestions and authors’ view. These are indicated in revised manuscript.

How do conclude that 94% had increased risk for cardiovascular complication? Because, you did not do any statistics test regarding risk for cardiovascular complication.

This is based on the WHO cutoff value for WHCR, which is predictive of the increased risk for cardiovascular complications. These has been indicated in many nutritional epidemiological studies and are very predictive for CVD risks.

Thanks for having a comment on the limitation of the study. Since mentioning the relevant limitation of the study is important to interpreted the study finding and its implication, we have mentioned and explained these limitations there.

Lastly, we have modified the recommendations to be specific to the specific body with a clear message. This is indicated in the conclusion part of the manuscript.

.... higher among those who were physically active (AOR = 1.60; 95% CI: 0.90–2.85) (Table 5).

This result seems strange when we see the direct relationship between exercise and IBG. However, as studies reported and we tried to discuss in the discussion, majority of the exercisers usually have some sort of non-communicable disease and obesity. In such cases, the individual might have an already IBG despite exercise. We noted this result in more detail there.

*Thank you very much!*

*Authors*
