# Supplementary material for: Major dietary patterns of community dwelling adults and their associations with impaired blood glucose and central obesity in Eastern Ethiopia: Diet-disease epidemiological study
Source: PLoS One. 2023 Apr 20;18(4):e0283075. doi: 10.1371/journal.pone.0283075 (PMC10118135; doi:10.1371/journal.pone.0283075)
Supplement: Attachment — Submitted filename: PONE-D-22-28499_R1_reviewer recommented.docx [file pone.0283075.s006.docx]

**PLOS ONE**

# Major dietary patterns of community dwelling adults and their associations with impaired blood glucose and central obesity in Eastern Ethiopia: diet-disease epidemiological study

--Manuscript Draft--

| **Manuscript Number:** | PONE-D-22-28499R1 |
| --- | --- |
| **Article Type:** | Research Article |
| **Full Title:** | Major dietary patterns of community dwelling adults and their associations with impaired blood glucose and central obesity in Eastern Ethiopia: diet-disease exploration study |
| **Short Title:** | Major dietary patterns and their associations with impaired blood glucose and central obesity |
| **Corresponding Author:** | Abdu Oumer  Dire Dawa University Dire Dawa, ETHIOPIA |
| **Keywords:** | Central obesity; Dietary Intake; dietary pattern; impaired blood glucose; PREDIABETES |
| **Abstract:** | Backgrounds: Unhealthy dietary intake is an important preventable risk factor for obesity and impaired blood glucose (IBG), ultimately increasing the risk of non- communicable diseases. When compared to individual food intakes, dietary patterns are a stronger predictor of health outcomes and should be systematically evaluated where such evidence is lacking. This study evaluated dietary patterns and their association with the risk of central obesity and IBG among adults.  Methods: A community-based survey was conducted among 501 randomly-selected adults from Eastern Ethiopia. Data was collected using a semi-structured questionnaire during a face-to-face interview that included sociodemographic and lifestyle factors, as well as a validated 89-item food frequency questionnaire (collected over one month). Principal component analysis was used to derive the dietary pattern. While central obesity was assessed using waist and/or hip circumference measurements, fasting blood sugar was used for IBG. A multivariable logistic regression model was fitted with an odds ratio, 95% confidence intervals, and p-values reported.  Results: A total of 501 adults (95.3%) were interviewed, with a mean age of 41 years (±12). Five major dietary patterns explaining 71% of the total variance were identified: “nutrient-dense foods”, “high fat and protein”, “processed foods”, “alcohol drinks”, and “cereal diets”. While 20.4% (17.0–24.2%) had IBG, 14.6% (11.8 –17.9) were centrally obese, and 94.6% (92.3–96.3) had an increased waist-to-hip circumference ratio.  Central obesity is associated with upper wealth status (AOR = 6.92; 2.91 –16.5), physical inactivity (AOR = 21.1; 2.77–161.4), a diet high in nutrient-dense foods (AOR  = 1.75; 0.75–4.06), processed foods (AOR = 1.41; 0.57–3.48), and cereal diets (AOR = 4.06; 1.87 – 8.82). The risk of IBG was associated with upper wealth status (AOR = 2.36; 1.36—4.10), physical inactivity (AOR = 2.17; 0.91–5.18), upper tercile of nutrient- dense foods (AOR = 1.35; 0.62–2.93), fat and protein diet (AOR = 1.31; 0.66–2.62), and cereal diet consumption (AOR = 3.87; 1.66–9.02).  Conclusion: IBG and central obesity were prevalent and predicted by upper tercile consumption of nutrient-dense foods, high fat and protein diets, processed foods, and cereal diets, which could guide dietary interventions. |
| **Order of Authors:** | Berhanu Abebaw |
|  | Abdu Oumer |
|  | Ahmed Ale |
|  | Aragaw Hamza |
|  | Imam Dagne |
|  | Abdurezak Adem Umer |
|  | Dilnessa Fentie |
|  |  |

|  | Muluken Yigezu |
| --- | --- |
|  | Zerihun Tariku |
|  | Shambel Abate |
| **Response to Reviewers:** | PONE-D-22-28499  Major dietary patterns of community dwelling adults and their associations with impaired blood glucose and central obesity in Eastern Ethiopia: diet-disease exploration study  Dear editor, please kindly note that we have added authors which have significant contribution to the current work. We have attached the PLOS One authorship change request form filled for your approval.  Dear editor and reviewers  Thank you very much for having such detail and valuable comments on our work. We have checked your comments and suggestion in more detail and have included them in the revised submission. All the changes are showed in track changes. Hence, we found it that giving a detail response for each point is not necessary. Thus, we only responded to question which need clarification ad included the requested changes in the revised manuscript simply.  On the title, the comment was reasonable and we amended the title accordingly. We also amended the comments on the abstract as it refers to percent based on WHCR cutoff point. These finding is discussed in more detail in the discussion part.  We specified the study design as survey or cross-sectional study design and the study period as “September 10 to November 06”.  You declared the final sample size as 526 in the section of sample size determination. Why you did you decide on 501?  The estimated minimum sample size required for this study was 526. However, the achieved response rate was 95% where a total 25 respondents were non-responders. They were not volunteer to participate in the study. The difference is due to this.  A separate multivariable model was done for IBG and central obesity as this is shown in Table 4 and 5. This is also clearly indicated there.  The interpretation of the odds ratio had some errors and we accepted the comments from the reviewers. Hence, we thoroughly edited the suggestions in way that the Odds ratio could be interpreted accordingly. Hence, we interpreted it as increased occurrence or odds of IBG or central obesity as it is indicated in the revised version.  The comments are well accepted.  Computing COR is not conclusive to determine determinants of IBG.  Here, we run both the crude and adjusted odds ratio. This could allow us to clearly indicate the statistical analysis and the reader in a very clear and reproducible way. Since we also clearly reported the adjusted association, this is very informative than the crude one. Hence, we made our conclusion and recommendations based on the adjusted associations. Presenting the crude one makes the paper more holistic and reproducible.  This discussion about the association between dietary pattern and central obesity and IBG in this study is not adequate. In addition, it is not exhaustive. You need to compare with similar finding and identify similar and unique variables. If this study identifies unique variable you need to have possible justification.  Here, as you mentioned the objective of the study is like that and we tried our best to present the discussion in a very clear and sequential manner. We started with IBG and central obesity, then their association with empirical dietary patterns. The major challenge was lack of such evidence in Ethiopia and Africa as well. That is why we mainly used gray literatures and fats to support and explain our results. But we have tried our best to modify the discussion as per the reviewers’ suggestions and authors’ view. These are indicated in revised manuscript.  How do conclude that 94% had increased risk for cardiovascular complication? Because, you did not do any statistics test regarding risk for cardiovascular complication.  This is based on the WHO cutoff value for WHCR, which is predictive of the increased risk for cardiovascular complications. These has been indicated in many nutritional epidemiological studies and are very predictive for CVD risks.  Thanks for having a comment on the limitation of the study. Since mentioning the |

|  | relevant limitation of the study is important to interpreted the study finding and its implication, we have mentioned and explained these limitations there.  Lastly, we have modified the recommendations to be specific to the specific body with a clear message. This is indicated in the conclusion part of the manuscript.  .... higher among those who were physically active (AOR = 1.60; 95% CI: 0.90–2.85) (Table 5).  This result seems strange when we see the direct relationship between exercise and IBG. However, as studies reported and we tried to discuss in the discussion, majority of the exercisers usually have some sort of non-communicable disease and obesity. In such cases, the individual might have an already IBG despite exercise. We noted this result in more detail there.  Thank you very much! Authors |
| --- | --- |
| **Additional Information:** |  |
| **Question** | **Response** |
| **Financial Disclosure**  Enter a financial disclosure statement that describes the sources of funding for the work included in this submission. Review the [submission guidelines](http://journals.plos.org/plosone/s/submission-guidelines#loc-financial-disclosure-statement) for detailed requirements. View published research articles from [*PLOS ONE*](http://journals.plos.org/plosone/) for specific examples.  This statement is required for submission and **will appear in the published article** if the submission is accepted. Please make sure it is accurate. | This study was funded by Dire Dawa University, under annual competitive research grant. The funders had no role in study design, data collection and analysis, the decision to publish, or the preparation of the manuscript. |
| **Unfunded studies**  Enter: *The author(s) received no specific funding for this work.*  **Funded studies**  Enter a statement with the following details:   - Initials of the authors who received each award - Grant numbers awarded to each author - The full name of each funder - URL of each funder website - Did the sponsors or funders play any role in the study design, data collection and analysis, decision to publish, or preparation of the manuscript? - **NO** - Include this sentence at the end of your statement: *The funders had no role in*   *study design, data collection and analysis, decision to publish, or preparation of the manuscript.*   - **YES** - Specify the role(s) played. |  |
|  |  |

| * typeset |  |
| --- | --- |
| **Competing Interests**  Use the instructions below to enter a competing interest statement for this submission. On behalf of all authors, disclose any [competing interests](http://journals.plos.org/plosone/s/competing-interests) that could be perceived to bias this work—acknowledging all financial support and any other relevant financial or non- financial competing interests.  This statement is required for submission and **will appear in the published article** if the submission is accepted. Please make sure it is accurate and that any funding sources listed in your Funding Information later in the submission form are also declared in your Financial Disclosure statement.  View published research articles from  [*PLOS ONE*](http://journals.plos.org/plosone/) for specific examples. | The authors have declared that no competing interests exist. |
| **NO authors have competing interests**  Enter: *The authors have declared that no competing interests exist.*  **Authors with competing interests**  Enter competing interest details beginning with this statement:  *I have read the journal's policy and the authors of this manuscript have the following competing interests: [insert competing interests here]* |  |
| * typeset |  |
| **Ethics Statement**  Enter an ethics statement for this submission. This statement is required if the study involved:   - Human participants - Human specimens or tissue | Ethical approval was obtained from the Institutional Research Ethical Review Board of Dire Dawa University. Written informed assent was obtained from all adults This research was conducted in accordance with the Helsinki Declaration for protecting human study subjects. |

| - Vertebrate animals or cephalopods - Vertebrate embryos or tissues - Field research   Write "N/A" if the submission does not require an ethics statement.  General guidance is provided below. Consult the [submission guidelines](http://journals.plos.org/plosone/s/submission-guidelines#loc-guidelines-for-specific-study-types) for detailed instructions. **Make sure that all information entered here is included in the Methods section of the manuscript.** |  |
| --- | --- |

| **Format for specific study types**  **Human Subject Research (involving human participants and/or tissue)**   - Give the name of the institutional review   board or ethics committee that approved the study   - Include the approval number and/or a statement indicating approval of this research - Indicate the form of consent obtained (written/oral) or the reason that consent was not obtained (e.g. the data were analyzed anonymously)   **Animal Research (involving vertebrate animals, embryos or tissues)**   - Provide the name of the Institutional Animal Care and Use Committee (IACUC) or other relevant ethics board that reviewed the study protocol, and indicate whether they approved this research or granted a formal waiver of ethical approval - Include an approval number if one was obtained - If the study involved *non-human primates*, add *additional details* about animal welfare and steps taken to ameliorate suffering - If anesthesia, euthanasia, or any kind of animal sacrifice is part of the study, include briefly which substances and/or methods were applied   **Field Research**  Include the following details if this study involves the collection of plant, animal, or other materials from a natural setting:   - Field permit number - Name of the institution or relevant body that granted permission |  |
| --- | --- |
|  |  |
| **Data Availability**  Authors are required to make all data underlying the findings described fully available, without restriction, and from the time of publication. PLOS allows rare exceptions to address legal and ethical concerns. See the [PLOS Data Policy](http://journals.plos.org/plosone/s/data-availability) and [FAQ](http://journals.plos.org/plosone/s/data-availability#loc-faqs-for-data-policy) for detailed information. | Yes - all data are fully available without restriction |

| A Data Availability Statement describing where the data can be found is required at submission. Your answers to this question constitute the Data Availability Statement and **will be published in the article**, if accepted. |  |
| --- | --- |
| **Important:** Stating ‘data available on request from the author’ is not sufficient. If your data are only available upon request, select ‘No’ for the first question and explain your exceptional  situation in the text box. |  |
| Do the authors confirm that all data underlying the findings described in their manuscript are fully available without restriction? |  |
| **Describe where the data may be found in full sentences. If you are copying our sample text, replace any instances of XXX with the appropriate details.**   - If the data are **held or will be held in a public repository**, include URLs, accession numbers or DOIs. If this information will only be available after acceptance, indicate this by ticking the box below. For example: *All XXX files*   *are available from the XXX database (accession number(s) XXX, XXX.*).   - If the data are all contained **within the**   **manuscript and/or Supporting Information files**, enter the following: *All relevant data are within the*  *manuscript and its Supporting Information files.*   - If neither of these applies but you are   able to provide **details of access elsewhere**, with or without limitations, please do so. For example:  *Data cannot be shared publicly because of [XXX]. Data are available from the XXX Institutional Data Access / Ethics Committee (contact via XXX) for researchers who meet the criteria for access to confidential data.*  *The data underlying the results presented in the study are available from (include the name of the third party* | All relevant data are included within the manuscript and its supporting Information files. |

| *and contact information or URL).*   - This text is appropriate if the data are owned by a third party and authors do not have permission to share the data.   * typeset |  |
| --- | --- |
| Additional data availability information: |  |

| Manuscript | [Click here to access/download;Manuscript;Manuscript.docx](https://www.editorialmanager.com/pone/download.aspx?id=32603017&amp;guid=6d7e4482-3bac-4804-b99f-b176f3730c40&amp;scheme=1) |
| --- | --- |
| 1 | Major dietary patterns of community dwelling adults and their associations with |
| 2 | impaired blood glucose and central obesity in Eastern Ethiopia: diet-disease |
| 3 | epidemiological study |
| 4 | Berhanu Abebaw^1^, Abdu Oumer^2*^, Ahmed Ale^3^, Aragaw Hamza^4^, Imam Dagne^2^, Abdurezak |
| 5 | Adem Umer^2^, Dilnessa Fentie^3^, Muluken Yigezu^2^, Zerihun Tariku^2^, and Shambel Abate^2^ |
| 6 | 1 Department of Nutrition and dietetics, School of Public Health, Bahir Dar University, Ethiopia |
| 7 | ^2^Department of Public Health, College of Medicine and Health Sciences, Dire Dawa University, |
| 8 | Dire Dawa, Ethiopia |
| 9 | ^3^School of Medicine, College of Medicine and Health Sciences, Dire Dawa University, Dire |
| 10 | Dawa, Ethiopia |
| 11 | ^4^Department of Anesthesia, College of Medicine and Health Sciences, Dire Dawa University, |
| 12 | Dire Dawa, Ethiopia |
| 13 | *Corresponding author:* Abdu Oumer; department of Public Health, College of Medicine and |
| 14 | Health Sciences, Dire Dawa University, Dire Dawa, Ethiopia; Email: [omab2320@gmail.com](mailto:omab2320@gmail.com) |
| 15 | Abstract |
| 16 | Backgrounds: Unhealthy dietary intake is an important preventable risk factor for obesity and |
| 17 | impaired blood glucose (IBG), ultimately increasing the risk of non-communicable diseases. |
| 18 | When compared to individual food intakes, dietary patterns are a stronger predictor of health |
| 19 | outcomes and should be systematically evaluated where such evidence is lacking. This study |
| 20 | evaluated dietary patterns and their association with the risk of central obesity and IBG among |
| 21 | adults. |
| 22 | Methods: A community-based survey was conducted among 501 randomly-selected adults from |
| 23 | Eastern Ethiopia. Data was collected using a semi-structured questionnaire during a face-to-face |
| 24 | interview that included sociodemographic and lifestyle factors, as well as a validated 89-item food |
| 25 | frequency questionnaire (collected over one month). Principal component analysis was used to |
| 26 | derive the dietary pattern. While central obesity was assessed using waist and/or hip circumference |

27 measurements, fasting blood sugar was used for IBG. A multivariable logistic regression model
28 was fitted with an odds ratio, 95% confidence intervals, and p-values reported.
29 Results: A total of 501 adults (95.3%) were interviewed, with a mean age of 41 years (±12). Five
30 major dietary patterns explaining 71% of the total variance were identified: “nutrient-dense foods”,
31 “high fat and protein”, “processed foods”, “alcohol drinks”, and “cereal diets”. While 20.4% 32	(17.0–24.2%) had IBG, 14.6% (11.8 –17.9) were centrally obese, and 94.6% (92.3–96.3) had an
33 increased waist-to-hip circumference ratio. Central obesity is associated with upper wealth status
34 (AOR = 6.92; 2.91 –16.5), physical inactivity (AOR = 21.1; 2.77–161.4), a diet high in nutrient-
35 dense foods (AOR = 1.75; 0.75–4.06), processed foods (AOR = 1.41; 0.57–3.48), and cereal diets
36 (AOR = 4.06; 1.87 – 8.82). The risk of IBG was associated with upper wealth status (AOR = 2.36;
37 1.36—4.10), physical inactivity (AOR = 2.17; 0.91–5.18), upper tercile of nutrient-dense foods
38 (AOR = 1.35; 0.62–2.93), fat and protein diet (AOR = 1.31; 0.66–2.62), and cereal diet 39	consumption (AOR = 3.87; 1.66–9.02).
40 Conclusion: IBG and central obesity were prevalent and predicted by upper tercile consumption
41 of nutrient-dense foods, high fat and protein diets, processed foods, and cereal diets, which could
42 guide dietary interventions. 43

44	Key words: central obesity; dietary intake; dietary pattern; impaired blood glucose; prediabetes 45

46

47

48

49

50

51

52

53

54

55

## 56 Major dietary patterns of community dwelling adults and their associations with

57 impaired blood glucose and central obesity in Eastern Ethiopia: diet-disease
58 epidemiological study
59 1. Introduction
60 Now a day the lifestyle characteristics of individuals, including dietary intake, change from time
61 to time dramatically. At the same time, the occurrence of non-communicable diseases (NCDs)
62 associated with impaired blood glucose (IBG) and obesity is rising (1). Hence, these are early
63 indicators of increased risk of NCDs and their complications (2, 3). NCDs are responsible for the
64 deaths of 41 million people (71%) each year, with low- and middle-income countries accounting
65 for more than 85% of all deaths. Obesity and high blood glucose kill about 17.9 and 1.6 million
66 people, respectively (4). The burden of NCDs has significantly increased, mainly attributable to
67 dietary habit change, increasing obesity and poor lifestyles as a result of poor lifestyle practices
68 (5). These are becoming major public health problems, especially in developing countries,
69 accounting for an estimated 80% of all mortality (6).
70 Prediabetes or IBG, defined as fasting blood glucose (FBS) of 100-126 mg dl^-1^, is a precursor to
71 established diabetes mellitus, which could be aggravated by insulin resistance due to abdominal
72 obesity (7). In particular, central obesity, with a higher waist circumference (WC) and/or waist-to-
73 hip circumference ratio (WHCR), is the sole cause of many NCDs related morbidity and mortality
74 (8). Among the risk factors, dietary consumption is a long-time exposure and one of the modifiable
75 and preventable exposures for overt NCDs (3), which needs to be investigated systematically (9).
76 Dietary risk factors were responsible for an estimated 11 million (10–12) deaths and 255 million
77 (234–274) DALYs lost; including high sodium intake, (3 million deaths), low intake of whole
78 grains (3 million deaths) and low intake of fruits (2 million deaths) (9).
79 Due to the methodological shortcomings of individual food or nutrient analysis for predicting
80 health outcomes, dietary pattern analysis is a better approach to characterize dietary consumption.
81 It is becoming a more practical approach to determine the role of diet in the prevention and
82 treatment of chronic diseases (2), taking into account the confounding, interaction, and synergistic
83 effects of nutrients in food that make causation difficult (10, 11).	It helps to evaluate the
84 cumulative and interactive effects of each dietary compound in a food and to consider the
85 complexity of the diet (10, 12).
86 Hence, dietary patterns could be more predictive for IBG and central obesity. Central obesity and
87 IBG are the major risk factors that increase the risk of NCDs, including diabetes mellitus. NCDs
88 are a public health problem in Ethiopia, where 4.2% are victims of diabetes mellitus, causing 70%
89 of overall deaths (13). While an estimated up to 39% are centrally obese, 5% of overall mortality
90 is due to IBG (14). In Ethiopia, an estimated 20.7% and 6.9% of adults are overweight and obese
91 (15), where 35 to 38% of adults have central obesity (16). This huge burden of central obesity, is
92 one main risk factor for established NCDs and all their complications (17, 18). For instance, an
93 estimated 25% of IBG will progress to diabetes while 50% remain in an impaired state (19, 20)
94 and increases cardiovascular mortality by 43% (21). The rate of progression to visible diseases is
95 rapid, necessitating a targeted intervention to slow progression.
96 Given the huge role of diet in the development of central obesity and IBG, there is a need to
97 investigate the link in the absence of such evidence in Ethiopia. The study area is characterized by
98 physical access to diversified diet from agricultural productivity, industrial processing and imports.
99 Studies conducted in other countries showed a clear link between dietary patterns and the risk of
100 central obesity and IBD (22-25). However, evidence from other countries may not be applicable
101 to our case, where dietary habits greatly vary even from region-to-region. In addition, studies
102 conducted so far mainly focus on individual food consumptions (13, 16, 26, 27), which cannot be
103 powerful predictors. This would help to design appropriate context-specific dietary strategies to
104 decrease the risk of central obesity and IBG in Ethiopia. This study was to evaluate the association
105 between major dietary patterns and central obesity and IBG among adults in Ethiopia.

## 106 2. Materials and Methods

### 107 2.1. Study Area and Period

108 This study was conducted in Dire Dawa and Harar, located in the eastern parts of Ethiopia. Harar
109 is located 526 kilometers from the capital city, Addis Ababa. Dire Dawa has an estimated
110 population of more than 0.5 million, while more than 0.3 million people reside in the Harari
111 regions, where more than half of the population resides in urban areas. According to the 2010
112 Harari Region population projection, there are 250, 093 with 146, 913 living in the urban areas
113 and 122, 942 are males, with a total household of 64, 334. These two regions have a multiethnic
114 and diverse population with a heterogenous agricultural productivity for fruits, vegetables,
115 sorghum, maize, wheat, and other cereals. Furthermore, being an industrial center for many
116 manufacturing industries and a trade center closest to neighboring countries makes access to
117 imported foods easier, in addition to domestic production. Hence, rice, white flour, macaroni, and
118 other industrially processed foods are accessible, in addition to cereals and grains marketed from
119 other regions of the country. The study was conducted from September 10 to November 06, 2021.
120 The study period was considered the fasting and feasting period to capture habitual Cereals,
121 including teff, wheat, and legumes, were the most staple crops in the study areas. In addition, some
122 processed foods, potatoes, and other fruits are the major foods consumed in the study area.

### 123 2.2. Study design and population

124 A community-based cross-sectional study was conducted among 501 randomly selected adults
125 (aged >= 18 years) from the two study sites available during the study period. Hence, the findings
126 of this study would be generalized to all adults residing in Harar and Dire Dawa towns, Eastern
127 Ethiopia. Adults who were pregnant, had abdominal swelling (ascites), or had detectable thoracic
128 and lumbar spinal deformity were excluded because waist circumference (WC) measurements are
129 biased. Also, clients with serious psychiatric disorders, which make them unable to give oriented
130 responses and unable to communicate, were excluded. More importantly, adults with clinically
131 diagnosed diabetes and/or known diabetes patients on treatment were also excluded from the study.

### 132 2.3. Sample size determination and sampling procedures

133 The minimum sample size for this study was estimated using a sample size formula for single
134 proportion with ”P” as the prevalence of central obesity from the previous study at 24.4% (26), at
135 a 95% confidence level, a “Z” critical value of 1.96, and a marginal error of “d” of 5%. The sample
136 size became 145. The sample size for the association of dietary pattern with central obesity and
137 IBG was calculated using the StatCalc module in Epi Info software version 7.0. After checking for
138 many risk factors, the maximum sample size calculated was taken considering a prudent dietary
139 pattern for predicting IBG (AOR = 2.26), abnormal fasting blood glucose level (OR = 2.26),
140 percentage of IBG among those with a healthy dietary pattern (24%) (28), power of 80%, and
141 equal sample of potential exposed and unexposed groups. Thus, taking dietary pattern as a risk
142 factor for IBG and central obesity, the sample size became 504. After adding a non-response rate
143 of 5%, the final minimum sample for this study was 526.
144 Stratified random sampling combined with simple and systematic random sampling was employed
145 to select study participants. The total sample size was proportionally allocated to the two study
146 sites (Dire Dawa and Harar) and to the smaller study units consecutively. A random sample of four
147 districts in Harar and four kebeles (fourth administrative division level in Ethiopia) were randomly
148 selected, where households were selected using systematic random sampling at a skipping interval.
149 The sample interval (K) was calculated by dividing the total number of households by the allocated
150 sample size for each kebele. Then, a random sample of adults residing in the selected household
151 was selected and interviewed. The first household was selected randomly, and adults residing in
152 randomly selected households were included in the study.

### 153 2.4. Data collection

154 Data were collected through a face-to-face interview using a set of pretested and structured
155 questionnaires, as well as standardized anthropometric measurements and FBS measurement. The
156 tool contains socio-demographic variables, lifestyle-related questions, and a validated semi-
157 quantitative Food Frequency Questionnaire (FFQ) to capture the dietary intake of adults over the
158 last one month. Data was collected by trained health care workers and graduating health students
159 through face-to-face interviews, measurements, and laboratory analysis. Data collectors got the
160 data by interviewing the study subject and probing for possible missed food consumed during the
161 specific period of time.
162 The 16-item validated General Physical Activity Questionnaire (GPAQ) tool developed by WHO
163 for physical activity surveillance was employed to assess the level of physical activity through
164 face-to-face interviews. The activity level of the study participants was evaluated according to the
165 standard WHO total physical activity calculation guide, and the level of total physical activity was
166 categorized as physically active or inactive as detailed in the previous section. After calculating
167 the metabolic equivalent per week (MET/week), a value below 600 was used to indicate physical
168 inactivity (29).
169 A modified semi-quantitative Helen Keller FFQ containing 89 food items with about nine food
170 groups was used to collect the dietary intake of study subjects. The FFQ was asked for the food
171 that the subject consumed over the past 1 month to capture reliable and habitual dietary
172 consumption of the individual. We further contextualized the list of food items for the particular
173 context by considering the local foods commonly produced and consumed **(Supplementary file**
174 **1).** The FFQ validated for the Ethiopian adults has been contextualized and pretested to capture
175 the dietary intake of adults. A validation study showed that the contextualized tool was valid and
176 reliable in measuring the dietary micro- and macronutrient intake of adults in Ethiopia (30). In
177 pilot analysis, the FFQ tool we assed was found to be highly reliable with a Cronbach’s alpha value
178 above 0.86, indicative of a reproducible tool (31).
179 For assessment of central obesity, WC and hip circumference (HC) measurements were taken by
180 trained anthropometric measurers using a non-stretchable tape meter at the end of a gentle
181 expiration. The clothing and jewelry of the clients will be kept minimal. The WC was measured at
182 a point midway between the lowest rib and the iliac crest in a horizontal plane at around the
183 umbilicus while the respondents were instructed to breath gently. The tape meter is not tightly
184 secured to avoid pressure and bias in measurement. While the HC was measured at the maximum
185 circumference of the pelvis over the great trochanters, Measurements were made at least twice,
186 and the average of the two measurements was recorded.
187 The laboratory analysis for blood glucose measurement was done by qualified and trained health
188 professionals. The blood glucose measurement was done using a 2 mL capillary blood sample
189 collected from the finger tips, and analyzed using a simple yet valid glucometer machine and
190 recorded as mg dl^-1^. A specific cut off point from 100 to 125 mg dl-1 was used to diagnose IBG or
191 prediabetes.

### 192 2.5. Data quality assurance

193 A pair of trained data collectors were deployed to collect the data from study subjects, as
194 anthropometry needs curious measurements. Training was given on appropriate interview
195 techniques and anthropometric measurements on WC and HC. Anthropometric reliability was
196 assessed on 10 study subjects during the pretest, and intra- and interobserver variations were
197 calculated. Data collectors with high inter and both intra-observer bias (technical error of
198 measurement above 2%) were retrained and collected the data once it was in the acceptable range
199 (32). All standard measuring procedures and instruments were strictly followed during data
200 collection. The standard operating procedure for blood sampling and glucose testing was followed
201 accordingly. During data entry into EpiData, the data quality was maintained by making legal
202 ranges, skipping patterns, appropriate coding, and careful data entry. A Piot study was conducted
203 on the unselected study area in order to test and correct the necessary corrections. The FFQ is
204 adapted and modified with the purpose of not missing relevant foods that are commonly consumed
205 in the study area (33).

### 206 2.6. Data analysis

207 After checking for completeness and inconsistencies, the collected data was entered into EpiData
208 Version 3.02 and exported to SPSS for analysis. All statistical analyses were carried out with
209 Microsoft Excel 2016 and IBM SPSS Statistics for Windows, Version 20.0 (IBM Corp., Armonk,
210 NY, USA). Descriptive statistics were computed for each variable, and the data was presented in
211 statistical tables and graphs. The WHCR was calculated from WC and HC and checked for
212 normality using the Kolmogorov-Smirnov test. Principal component analysis was conducted using
213 household assets to derive the socioeconomic status (wealth index) of adults. All assumptions of
214 PCA were checked using the Kaiser Mayer-Olkin (KMO) sampling adequacy, correlations, and
215 covariance parameters. Based on the derived factor scores, wealth status was categorized into five
216 quartiles, from the poorest to the wealthiest.
217 Dietary pattern analysis
218 Exploratory factor analysis using a principal component analysis was used to derive dietary
219 patterns using the average frequency of 89-item food consumption in the past one month. The
220 consumption frequency was recategorized into daily equivalents based on previous literature (34)
221 and weighted daily consumption equivalents (**Supplementary File 2**). In addition, some closely
222 related food items were summed up and recategorized into a single food item as needed. Bartlett’s
223 test of sphericity and the KMO measure of sampling adequacy were used to verify the
224 appropriateness of exploratory factor analysis. For the degree of intercorrelations between
225 variables, we used a KMO value of greater than 0.5 and a p-value of less than 0.05 for the Bartlett
226 test of sphericity. Orthogonally rotation using the varimax option was used to enhance the
227 interpretability of dietary patterns (35). The principal components were retained based on the
228 following criteria: factor eigenvalue, identification of a break point in the scree plot, the proportion
229 of variance explained, and interpretability of the dietary patterns (12, 36). A rotated factor loading
230 matrix was used to assess the direction and strength of correlation between each food item and its
231 components. We also checked for a complex association between one food items and multiple
232 factors, where such factors were removed. The corresponding factor score for each dietary pattern
233 was constructed by summing the observed daily frequency of consumption of individual food
234 items weighted by the factor loading. Hence, a high score of a component indicates more frequent
235 consumption of the foods constituting that particular dietary pattern. For further analysis, each
236 factor score was ranked into three terciles as low, medium, and high frequency of consumption.
237 Dietary pattern with central obesity and IBG
238 A separate multivariable logistic regression model was fitted to evaluate the association between
239 dietary pattern and central obesity and IBG. Both crude and adjusted odds ratios with 95%
240 confidence intervals and/or p-values were computed. Multicollinearity was checked using the
241 variance inflation factor (VIF) and/or the standard error estimates. The model fitness was checked
242 using Hosmer and Lemeshow’s goodness of fit. In addition, variables with a p-value below 0.2 in
243 bivariable logistic regression and biologically plausible risk factors for the outcomes were
244 considered in the multivariable logistic models. Statistical significance was declared at a p-value
245 below 0.05.

### 246 2.7. Variables of the study

247 While the dependent variables of this study were IBG and central obesity, the independent
248 variables were sociodemographic variables (age, sex, income, occupation, and education), physical
249 activity, family history of diabetes, dietary pattern, dietary intake of food groups, and habits of
250 substance use. The outcome variable IBG was categorized as IBG when the FBS between 100 and
251 125 mg dl^-1^ (37). Central obesity was defined as having a waist circumference of 94 cm for men
252 and 80 cm for women, or a WHCR of 0.90 in men and 0.85 in women (38).

### 253 2.8. Ethical approval

254 Ethical approval was obtained from Dire Dawa University, Institutional Research Review Board.
255 The support letter was taken to the respective regional health office for cooperation. A written
256 informed consent was obtained from each study participant after explaining the study purpose,
257 confidentiality, and procedures in detail. The study was conducted in accordance with the
258 Declaration of Helsinki for Human Research (39). The data will be used for this research purpose
259 only, and the data will be kept confidential. Respondents identified with central obesity and IBG
260 got counseling, recommendation, and/or linkage to the health facilities. All the COVID-19
261 standard prevention precautions were implemented throughout the data collection process.

## 264 3. Results

### 265 3.1. Sociodemographic characteristics

266 A total of 501 study subjects were included in the current survey, with a response rate of 95.3%.
267 More than half (66.7%) of the participants were females. The age of the respondents ranges from
268 18 –64 years, with a mean age of 41 years (±12). The majority, 90% of the respondents reside with
269 their families. A total of 203 (40.5%) attended primary school, and 143 (28.5%) and 127 (25.3%)
270 were housewives and government employees, respectively. About 188 (37.5%) and 44 (8.8%)
271 were in the wealthier and wealthiest ranks regarding socioeconomic status. On the other hand, 105
272 (21%) and 100 (20%) of the respondents were from the poorest and poorer households,
273 respectively (Table 1).
274 **Table 1.** sociodemographic characteristics of respondents for a dietary pattern study in Dire Dawa
275 and Harar, eastern Ethiopia

| Variables | Categories | Frequency | Percent |
| --- | --- | --- | --- |
| Sex | Male | 182 | 33.3 |
|  | Female | 319 | 66.7 |
| Live with | With family | 450 | 90 |
|  | Friends | 28 | 5.6 |
|  | Lonely | 21 | 4.4 |
| Education | Illiterate | 24 | 4.6 |
|  | Primary education | 203 | 40.5 |
|  | Secondary education | 107 | 21.4 |
|  | College and above | 167 | 33.3 |
| Marital status | Married | 409 | 81.6 |
|  | Single | 71 | 14.2 |
|  | Widowed | 21 | 4.2 |
| Occupation | Daily laborer | 18 | 3.6 |
|  | Farmer | 9 | 1.8 |
|  | Government employee | 127 | 25.3 |
|  | Housewife | 143 | 28.5 |
|  | Merchant | 69 | 13.8 |
|  | Private employed | 61 | 12.2 |

|  | Others | 74 | 14.8 |
| --- | --- | --- | --- |
| Wealth index | Poorest | 105 | 21.0 |
|  | Poor | 100 | 20.0 |
|  | Medium | 64 | 12.8 |
|  | Wealthier | 188 | 37.5 |
|  | Wealthiest | 44 | 8.8 |

276 3.2.	Physical activity and substance use related factors
277 Regarding the physical activity of adults, more than three-fourths (383, 76.8%) of them were
278 reported to be physically inactive with a median metabolic equivalent of 240 MET (IQR: 0–500
279 MET). While only 55 (11%) had a regular physical activity schedule, 85 (27.9%) had a family
280 history of diabetes mellitus. Approximately 304 (60.7%) reported chewing khat, 101 (20.2%)
281 reported drinking alcohol, and 24 (4.8%) reported chewing khat and smoking cigarettes.
282 3.3.	Dietary patterns of adults
283 A total of 465 (98%) of adults had a usual three-times-a-day meal, while 478 (98.4%) were
284 reported to have a regular meal. Almost all (99.4%) reported using liquid oils for cooking. About
285 95 (19%) and 48 (9.6%) reported having regular sweet and soda drinks and fatty foods,
286 respectively. In terms of fruit and vegetable consumption, only 96 (19.2%) reported regular fruit
287 consumption and 140 (27.9%) reported vegetable consumption (at least >= 6 times per week).
288 The consumption frequency was recategorized into daily equivalents based on previous literature
289 and weighted daily consumption equivalents. In addition, some closely related food items were
290 summed up and categorized into a single food item as needed. An exploratory factor analysis using
291 the principal component analysis method was conducted. Food items with a KMO value of below
292 0.30 were removed iteratively. All the assumptions of PCA were checked. The overall sample
293 adequacy was evaluated using KMO, where the p-value was 0.91. Similarly, the presence of
294 interitem correlation was checked using the Bartlett test of sphericity (p-value 0.0001), indicating
295 the presence of a statistically significant correlation. Furthermore, items with a communality below
296 0.30 were removed step-by-step. The final food items to be included were determined by the
297 following criteria: interpretability of the factors, improving the total variance explained, absence
298 of complex structure evidenced by rotation component matrix, eigen value, and scree plots

#### 299 (Supplementary File 3).

301 Five dietary patterns explaining 71% of the total variance were identified; “Animal source foods,
302 fruits, and vegetables-nutrient dense foods”, “high fat and protein foods”, “processed foods”,
303 “traditional alcoholic drinks”, and “cereal foods”. A total of sixteen, nine, seven, four and four
304 food items constitute the first, second, third, fourth, and fifth dietary patterns as indicted in
305 **Supplementary File 3**. Nutrient dense foods composed of animal source foods, fruits, and
306 vegetables account for the major part of the variance (42%), followed by high fat and protein foods
307 (11.2%). For further analysis, the standard score of each dietary pattern was categorized into three
308 terciles, where the lower score indicates less frequent consumption of foods and is assigned the
309 lowest tercile. Furthermore, traditional alcoholic drinks were retained as they are relevant to central
310 obesity and IBG (**Table 2****)**.
311 **Table 2**. Total variance explained and summary statistics for factor scores for identified dietary
312 patterns in Ethiopia

| S.no | Dietary  patterns | Label | Variance  explained | Median  (Q_2_) | Q_1_ | Q_3_ |
| --- | --- | --- | --- | --- | --- | --- |
| 1 | DP_1 | Animal source foods, | 42% | -0.250 | -0.358 | -0.180 |
|  |  | fruits, and vegetables- |  |  |  |  |
|  |  | Nutrient dense foods |  |  |  |  |
| 2 | DP_2 | High fat and protein | 11.2% | -0.036 | -0.241 | 0.079 |
|  |  | foods |  |  |  |  |
| 3 | DP_3 | Processed foods | 9.0% | -0.460 | -0.762 | 0.667 |
| 4 | DP_4 | Traditional alcoholic | 5.0% | -0.146 | -0.176 | -0.056 |
|  |  | drinks |  |  |  |  |
| 5 | DP_5 | cereal foods | 3.83% | -0.177 | -0.265 | 0.087 |
|  | Total |  | 71.0% | - | - | - |

313 3.4.	Magnitude of central obesity and impaired blood glucose
314 As clearly shown in Figure 1 below, an estimated 20.4% (95% CI: 17.0–24.2%) of the subjects
315 had impaired blood glucose or prediabetes. While 14.6% (95% CI: 11.8–17.9%) of adults had
316 central obesity based on the sex-specific WC cutoff points, 94.6 (95% CI: 92.3–96.3) of them had
317 a WHCR, which makes them at risk for any cardiovascular complications (Figure 1). When
318 disaggregated by sex, females had a higher rate of central obesity by WHCR (98% vs. 86%) and
319 WC (17.2% vs. 9.9%). Moreover, the prevalence of IBG was higher among males (22.5%)
320 compared to females (17.5%). The mean blood glucose level was 180.1 mg dl-1 (±50). Similarly,
321 the mean and standard deviation for WC and WHCR were 66.7 (±13.4) and 0.96 (±0.46).
323 **Figure 1.** The magnitude of impaired blood glucose, central obesity (based on WC cutoff points
324 of 80 and 94 cm for females and males, a WHC of 0.85 and 0.90 for females and males, b) among
325 adults residing in Dire Dawa and Harar, Eastern Ethiopia.
326 3.5.	Factors associated with central obesity and impaired blood glucose
327 An exploratory logistic regression was carried out to identify the association between
328 sociodemographic factors and dietary patterns with IBG and central obesity among adults, as
329 detailed in Table 3. Increasing age is associated with an increased odds of central obesity and IBG.
330 While males (COR = 1.24; 95% CI: 0.79–1.93) had 24% higher odds of IBG compared to females,
331 females (COR = 1.86; 1.05–3.30) had a significantly higher occurrence of central obesity. The

332	odds of IBG (COR = 2.18; 95% CI: 1.34–3.53) and central obesity (COR = 9.65; 95% CI: 4.29–

333 21.7) was 2.1 and 9.65-times higher in the wealthiest versus poorest families, respectively.
334 Similarly, housewives had 38% increased odds of IBG (COR = 1.38; 95% CI: 0.84–2.28) and
335 central obesity (COR = 2.33; 95% CI: 1.34–4.04) compared to adults engaged in private work. The
336 risk of central obesity was found to be high among those with irregular meals (COR = 1.68; 95%
337 CI: 0.60–4.66), physically inactive (COR = 1.54; 95% CI: 0.95–2.503), and habits of sweet food 338	consumption (COR = 1.63; 95% CI: 0.91–2.91) (Table 3).

339

340 **Table 3.** Bivariable logistic regression output for determinants of IBG and central obesity among
341 adults from Dire Dawa and Harar, Eastern Ethiopia.

| Factors | Categories | IBG (pre  diabetes) | | COR (95% CI) | Central obesity  (WC) | | AOR (95% CI) |
| --- | --- | --- | --- | --- | --- | --- | --- |
|  |  | IBG | No  IBG |  | Yes | No |  |
| Age in years | | – | – | 1.01 (0.99-1.03) | – | – | 1.03 (1.01-1.1) |
| Sex | Female | 61 | 258 | 1 | 55 | 264 | 1.86 (1.05-3.30)  * |
|  | Male | 41 | 141 | 1.24(0.79-1.93) | 18 | 164 | 1 |
| Wealth index | Poor | 30 | 175 | 1 | 7 | 198 | 1 |
|  | Medium | 9 | 55 | 0.96 (0.43-2.13) | 7 | 57 | 3.47(1.17-10.3) * |
|  | Wealthier | 63 | 169 | 2.18(1.34-3.53) * | 59 | 173 | 9.65(4.29-21.7) * |
| Occupation | Housewife | 35 | 106 | 1.38(.84-2.28) | 34 | 107 | 2.33(1.34-4.04) * |

|  | Government | 22 | 105 | 0.88(.50-1.54) | 11 | 116 | 0.69(0.33-1.45) |
| --- | --- | --- | --- | --- | --- | --- | --- |
|  | Private | 45 | 188 |  | 28 | 205 | 1 |
| Regular  meals | No | 1 | 23 | 1 | 5 | 18 | 1.68(0.60-4.66) |
|  | Yes | 102 | 375 |  | 68 | 410 | 1 |
| Physical  activity | Physically  active | 31 | 88 | 1.54(0.95-2.50) | 1 | 118 | 1 |
|  | Physically  inactive | 71 | 311 | 1 | 72 | 310 | 1.54(0.95-2.503) |
| Sweet and soda drinks | No | 75 | 331 | 1 | 54 | 352 | 1 |
|  | Yes | 27 | 68 | 1.75(1.05-2.92) * | 19 | 76 | 1.63(0.91-2.91) |
| Regular physical  activity | No | 94 | 352 | 1.57(0.72-3.43) | 64 | 382 | 0.86(0.40-1.83 |
|  | Yes | 8 | 47 | 1 | 9 | 46 |  |
| Nutrient dense foods (DP-1) | Lowest | 45 | 119 | 1.76(1.05-2.95) * | 27 | 137 | 1 |
|  | Middle | 26 | 136 | 0.89(0.50-1.57) | 6 | 156 | 0.20(0.08-0.49) |
|  | Highest | 31 | 144 | 1 | 40 | 135 | 1.50(0.87-2.59) |
| High fat and protein diet (DP-2) | Lowest | 41 | 126 | 1.70(1.0-2.92) | 33 | 134 | 1.63(0.91-2.94) |
|  | Middle | 34 | 132 | 1.35(0.77-2.35) | 18 | 148 | 0.81(0.42-1.57) |
|  | Highest | 27 | 141 | 1 | 22 | 146 | 1 |
| Processed foods (DP-3) | Lowest | 18 | 148 | 1 | 20 | 146 | 1 |
|  | Middle | 32 | 137 | 1.92(1.03-3.58) * | 21 | 148 | 1.04(0.54-1.99) |
|  | Highest | 52 | 114 | 3.75 (2.08-6.76) * | 32 | 134 | 1.74(0.95-3.20) |
| Alcoholic  drinks (DP- 4) | Lowest | 34 | 133 | 1 | 24 | 142 | 1 |
|  | Middle | 31 | 132 | 1.37(0.82-2.28) | 13 | 150 | .51(0.25-1.05) * |
|  | Highest | 37 | 134 | 0.68(0.38-1.20) | 36 | 136 | 1.57 (0.89-2.76) |
| Cereal diets (DP-5) | Lowest | 34 | 133 | 1 | 13 | 154 | 1 |
|  | Middle | 31 | 132 | .092(0.53-1.58) | 17 | 146 | 1.38 (0.65-2.94) |
|  | Highest | 37 | 134 | 1.08(0.64-1.82) | 43 | 128 | 3.98(2.05-7.72) * |

342 *Note: * statistically significant association at p-value <0.05.*
343 Dietary patterns were found to be associated with higher odds of central obesity and IBG among
344 adults. Adults who consumed less nutrient dense food (COR = 1.76; 95% CI: 1.05–2.95) and more
345 fat and protein-rich foods (COR = 1.35; 95% CI: 0.77–2.35), for example, had a 1.76- and 1.35–
346 times higher magnitude of IBG, respectively. More frequent consumption of processed foods
347 (COR = 3.75; 95% CI: 2.08–6.76) and medium consumption of traditional alcoholic drinks (COR

348	= 1.37; 95% CI: 0.82–2.28) were significant predictors of IBD. However, optimal consumption of

349 a cereal diet was protective against IBG. Furthermore, eating nutrient-dense foods (COR = 1.50;
350 95% CI: 0.87–2.59) and highly processed foods (COR = 1.74; 95% CI: 0.95–3.20) increased the
351 Likelihood of central obesity by 50% and 74%, respectively. Frequent consumption of traditional
352 alcoholic drinks (COR = 1.57; 95% CI: 0.89–2.76) and consumption of cereal diets (COR = 3.98
353 (2.05–7.72)) was shown to increase the occurrence of central obesity by 57% and 258%. However,
354 in a dietary pattern characterized by fat and protein-rich foods, the risk for both central obesity 355	(COR = 1.70; 95% CI: 1.0–2.92) and IBG (COR = 1.63; 95% CI: 0.91–2.94) was higher among
356 those with a lower tercile (Table 3).
357 A separate multivariable logistic model was fitted in a step-wise backward regression approach
358 for central obesity and IBG. Sex, wealth status, physical activity level, and dietary patterns 1, 3,
359 and 5 were found to be associated with central obesity among adults. Being physically inactive 360	(600 MET) (AOR = 21.1; 95% CI: 2.77–161.4), from a higher socioeconomic class (6.92 (2.91–
361 16.5), and females (AOR = 1.67 (0.82–3.40) were positively associated with a higher occurrence
362 of central obesity. More importantly, those who consumed more nutrient-dense foods (1.75; 95% 363	CI: 0.75 –4.06), processed foods (AOR = 1.41 (0.57–3.48), and cereal diets (AOR = 4.06; 95%
364 CI: 1.87–8.82) had a higher odd of central obesity. An increasing dose-response relationship was
365 observed where odds of its occurrence was higher for more frequent consumption (Table 4).
366 **Table 4.** Adjusted association between dietary patterns and central obesity among adults from Dire
367 Dawa and Harar, Eastern Ethiopia.

| Factors | Categories | Central obesity (WC) | | AOR (95% CI) | p-value |
| --- | --- | --- | --- | --- | --- |
|  |  | Yes | No |  |  |
| Sex | Female | 55 | 264 | 1.67(0.823.40) | 0.157 |
|  | Male | 18 | 164 | 1 |  |
| Wealth index | Poor | 7 | 198 | 1 |  |
|  | Medium | 7 | 57 | 2.56(0.80-8.25* | 0.115 |
|  | Wealthier | 59 | 173 | 6.92(2.91-16.5) | 0.001** |
| Physical activity | Physically active | 1 | 118 | 1 |  |
|  | Physically inactive | 72 | 310 | 21.1(2.77-161.4) | 0.003** |
| Nutrient dense foods (DP-1) | Lowest | 27 | 137 | 1 |  |
|  | Middle | 6 | 156 | 0.28(0.09-0.87) | 0.028* |
|  | Highest | 40 | 135 | 1.75(0.75-4.06) | .193 |
| Processed foods (DP-3) | Lowest | 20 | 146 | 1 |  |
|  | Middle | 21 | 148 | 1.59(0.73-3.46) | 0.246 |
|  | Highest | 32 | 134 | 1.41(0.57-3.48) | 0.456 |
| Cereal diets (DP-5) | Lowest | 13 | 154 | 1 |  |
|  | Middle | 17 | 146 | 1.51(0.66-3.45) | 0.325 |
|  | Highest | 43 | 128 | 4.06(1.87-8.82) * | 0.0001** |

368 Note: statistically significant factors associated with central obesity at p-value <0.05* and 0.001^**^
369 A multivariable logistic regression output was fitted using Hosmer and Lemeshow’s goodness of
370 fit (P-value = 0.847), indicating a fit model. Those from wealthier families (AOR = 2.36; 95% CI:
371 1.36–4.10), those without regular physical activity (AOR = 2.17; 95% CI: 0.91–5.18) and those
372 with sweet food consumption habits (AOR = 2.46; 95% CI: 1.30–4.66) had a 2.36-, 2.17-, and
373 2.46-times increased odds of having IBG level compared to counterparts. Among the identified
374 dietary patterns, lower consumption of nutrient-dense foods (AOR = 1.35; 0.62–2.93), higher fat
375 and protein diets (AOR = 1.31; 95% CI: 0.66–2.62) and higher cereal diet consumption (AOR =
376 3.87; 95% CI: 1.66 –9.02) were important dietary determinants of IBG level. On the other hand,
377 the odds of IBG were 60% higher among those who were physically active (AOR = 1.60; 95% CI: 378	0.90–2.85) (Table 5).
379 **Table 5. Adjusted** multivariable logistic regression showing factors associated with IBG among adults residing
380 in Dire Dawa and Harar, Eastern Ethiopia
381

| Factors | Categories | IBG (pre diabetes) | | AOR (95% CI) | P-value  382 |
| --- | --- | --- | --- | --- | --- |
|  |  | IBG | No IBG |  |  |
| Wealth index | Poor | 30 | 175 | 1 |  |
|  | Medium | 9 | 55 | 1.29(0.54-3.04) | 0.567383 |
|  | Wealthier | 63 | 169 | 2.36(1.36-4.10) | 0.002* |
| Physical activity | Physically active | 31 | 88 | 1.60(0.90-2.85) | 0.107384 |
|  | Physically inactive | 71 | 311 | 1 |  |
| Sweet and soda drinks | No | 75 | 331 | 1 | 385 |
|  | Yes | 27 | 68 | 2.46(1.30-4.66) | 0.006* |
| Regular physical activity | No | 94 | 352 | 2.17(0.91-5.18) | 0.083 86 |
|  | Yes | 8 | 47 | 1 | 387 |
| Nutrient dense foods (DP-1) | Lowest | 45 | 119 | 1.35(0.62-2.93) | 0.490  388 |
|  | Middle | 26 | 136 | 1.27(0.65-2.5) |  |
|  | Highest | 31 | 144 | 1 | 389 |
| High fat and protein diet (DP-2) | Lowest | 41 | 126 | 1.42(0.75-2.71) | 0.285 |
|  | Middle | 34 | 132 | 1.31(0.66-2.62) | 0.443*^90^ |
|  | Highest | 27 | 141 | 1 |  |
| Processed foods (DP-3) | Lowest | 18 | 148 | 1 |  |
|  | Middle | 32 | 137 | 1.97(1.01-3.86) | 0.048_3_*_92_ |
|  | Highest | 52 | 114 | 3.87(1.66-9.02) | 0.002** |

391

393 *Note: statistically significant factors associated with central obesity at p-value <0.05^*^ and 0.001^**^*

## 394 4. Discussion

395 The burden of NCDs is rising at an alarming rate, accounting for more than 70% of adult mortality
396 (40). Prediabetes and central obesity associated with metabolic syndrome are the most important
397 risk factors for the development of overt NCDs (41). These diseases are in turn dependent on the
398 lifestyle and dietary consumption habits of individuals, which could be modified for a better health
399 condition (42). Hence, we conducted the current study to identify the role of dietary patterns in the
400 development of prediabetes (IBG) and central obesity among adults.
401 In this study, we found that 20.4% had IBG, 14.6% had central obesity, and 94% had an increased
402 risk for cardiovascular diseases based on the sex-specific WHCR cutoff point. This indicates IBG
403 is a common public health problem, especially among females, and it may be cross linked with a
404 comparable occurrence of central obesity (abdominal obesity) based on WC cutoff points.
405 Evidence from Ethiopia also showed that the prevalence of IBG ranges from 8% (43) to 12% (44).
406 It should be emphasized that central obesity is a main risk factor for insulin resistance and the
407 development of IBG. Since 85% of NCD deaths occur in low- and middle income countries (4),
408 multilateral dietary and lifestyle interventions are required to halt central obesity and IBG. This
409 will be needed to decrease the incidence of metabolic risk factors before an overt metabolic disease
410 (cardiovascular or diabetes) happens. A higher prevalence of central obesity (39%) was reported
411 in southern Ethiopia (45).
412 In the present study, being female, higher socioeconomic class (AOR = 6.92; 95% CI: 2.91–16.5),
413 and physical inactivity (AOR = 21.1; 95% CI: 2.77–161.4) were found to be important predictors
414 of central obesity. A similar association was also identified with IBG, except that being physically
415 active is associated with a higher risk of IBG. This was also supported by findings from Ethiopia, 416	where females had a 9-times likelihood (AOR = 9.62; 95% CI: 4.84–19.12) (16) and 12-times likelihood (AOR = 12.93,
417 95% CI: 6.74–24.79) higher chance of having of obesity than males. A similar association was also reported for
418 socioeconomic class (46), where poor lifestyles and access to and consumption of unhealthy foods
419 are more common. In addition, physical activity and having regular physical activity had a crucial
420 for maintaining energy balance. A study indicated that adults who were physically active were less likely to have
421 central obesity (AOR = 0.87: p-value 0.174), emphasizing the need for regular
422 physical activity (46).
423 On the other hand, the role of non-exercise energy expenditures like working, walking, and others
424 should be encouraged as these are very crucial for maintaining optimal fat storage (47, 48).
425 However, the negative association with IBG could be explained by the fact that those with some
426 risk of cardiovascular complications might have poor blood sugar levels. This does not invalidate
427 the positive role of physical activity for optimal weight and blood glucose. Rather, it reflects the
428 characteristics of the study subjects.
429 Although there is evidence for the link between some dietary consumption habits and central
430 obesity and IBG (49), more concrete and context-specific evidence using a more robust approach
431 is needed to guide obesity and diabetes prevention strategies (50). Due to the complex and
432 multicollinear nature of the human diet and the limitations of individual food or nutrient analysis
433 for predicting a particular health outcome, dietary pattern analysis using a more robust statistical
434 approach is becoming a novel approach for better recommendations (12, 36). Hence, we derived
435 the dietary patterns and its association with the development of central obesity and IBG.
436 Those who reported consuming processed foods (AOR = 1.41; 95% CI: 0.57–3.48) and cereal diets
437 (AOR = 4.06; 95% CI: 1.87–8.82) were a major risk factor for the development of central obesity.
438 Other studies indicated that the top tercile of western pattern consumption (OR = 1.82; 95% CI:
439 1.16–2.87) increased the risk for obesity while a prudent pattern (AOR = 0.62; 95% CI: 0.40–0.96)
440 reduced their risk for central obesity (51). On the other hand, those with a low consumption of
441 nutrient dense foods (animal sources, fruits, and vegetables) (AOR = 1.35; 95% CI: 0.62–2.93)
442 had a 35% increased risk of IBG compared to those with a higher tercile. This could be explained
443 by undernourished adults' metabolic limitations and an increased risk of insulin insufficiency for
444 glucose metabolism (52).
445 Other studies also showed that dietary patterns were highly correlated with abnormal blood glucose
446 status (r = 0.8) (53). A review of the evidence also found that healthy, traditional, and
447 Mediterranean diets rich in sea foods, fruits, and vegetables were effective in preventing IBG (54).
448 Similarly, higher adherence to the unhealthy dietary pattern (saturated fats and meat) was more
449 likely to cause insulin resistance and IBG as a result (49). On the contrary, a vegetarian dietary
450 pattern reduced the risk of IBG (p-value below 0.05) (28). For instance, a study showed that a
451 higher tercile of the western dietary pattern was associated with an increased risk of obesity (AOR

452	= 1.80; 95% CI: 1.15–2.81) compared to a lower tercile (55).

453 The association between lower tercile consumption of nutrient-dense foods with IBG could be
454 related to the issue of food security and the risk of having unhealthy dietary patterns. For example,
455 a study found that food insecurity could lead to unhealthy dietary patterns and ultimately increase
456 the risk of central obesity (56). The current study depicted the role of dietary pattern in the
457 development of central obesity and prediabetes. These could help to design and indicate an
458 effective dietary strategy to prevent obesity and its associated consequences (57).
459 Strength and Limitations of the study
460 The current study is novel and would fill existing knowledge gaps regarding concrete evidence on
461 the relationship between dietary pattern and central obesity and IBG. Furthermore, we employed
462 a more reliable dietary assessment approach and dietary pattern analysis using a more appropriate
463 and feasible analytic strategy. However, the findings of this study should be in the light of some
464 Methodological limitations. First, in assessing food consumption, the potential for recall bias and
465 respondent bias could not be excluded, which might affect bias the overall dietary consumptions.
466 Moreover, the current study did not collect quantitative data on daily intake to quantify the nutrient
467 intake beyond food consumption. The current study mainly focuses on the urban setting, where
468 the disease burden could be higher. However, a comparative study including urban and rural
469 setting could better show the association in a very informative way.

## 470 5. Conclusion and recommendations

471 Overall, the occurrence of central obesity, IBG, and the risk for cardiovascular diseases were
472 prevalent in the study area. Dietary patterns characterizing the overall diet of individuals are more
473 predictive of these outcomes, which could be replicated for other relevant diet- disease studies to
474 inform policy and strategies targeting the risks for non-communicable disease. Higher tercile
475 consumption of nutrient dense foods, high fat and protein diets, processed foods, and cereal diets
476 are important risk factors for central obesity and IBG. Optimal consumption of a diversified diet
477 from animal sources, fruits, vegetables, cereals, and protein-rich foods could play a great role in
478 preventing the occurrence of metabolic syndromes, while consumption of processed foods and
479 alcoholic drinks should be kept to a minimum. The findings of this study will inform the existing
480 policies and programs targeting non-communicable disease prevention in Ethiopia. We strongly
481 recommend regional health bureaus, non-governmental organizations working on nutrition, and
482 other concerned bodies to strategically implement appropriate behavioral change communication
483 and agricultural strategies healthy diet for the wider community.

#### 484 Declarations

485 **Ethical approval and consent to participate**
486 Ethical approval was obtained from the Institutional Research Ethical Review Board of Dire Dawa
487 University. Written informed assent was obtained from all adults This research was conducted in
488 accordance with the Helsinki Declaration for protecting human study subjects.

#### 489 Consent to publish

490 Not applicable

#### 491 Acknowledgment

492 We are grateful to Dire Dawa University and Dire Dawa Health Bureau for their collaborations
493 and support for the successful completion of the study. Our gratitude also goes to the respective
494 health	bureaus, respondents, data	collectors, and	supervisors	for their	sincere help and
495 collaboration for the successful completion of the research.
496 Author contribution
497 AO- participated in conceptualization, fund acquisition, designing the study, data curation,
498 resource management, methodology, validation, supervision, project administration, data analysis,
499 writing the manuscript, and reviewing and approving the manuscript. AA, AH, BA, ID, AAU, DF,
500 MY, ZT, and SA: participated in conceptualization, validation, supervision, methodology, data
501 preparation, and data visualization. In addition, AO has substantially contributed to cleaning,
502 preparing, performing the whole statistical analysis, preparation of tables and graphs, writing, and
503 drafting the manuscript, critically reviewing, revising, and editing the manuscript. All authors have
504 reviewed and approved the final manuscript and decided on the journal to submit to.

#### 505 Data Availiability

506 All relevant data are included within the manuscript and its supporting Information files.

#### 507 Funding

508 This study was funded by Dire Dawa University, under annual competitive research grant. The
509 funders had no role in study design, data collection and analysis, the decision to publish, or the
510 preparation of the manuscript.

#### 511 Competing interests

512 The authors have declared that no competing interests exist.

#### 513 Abbreviations:

514 CI–Confidence Interval; DP–Dietary Patterns; FFQ–Food Frequency Questionnaire; GPAQ–
515 General Physical Activity Questionnaire; IBG; HC–Hip Circumference; Impaired Blood Glucose;
516 K–Sampling	Interval; METS–Metabolic Equivalents; PCA–Principal	Component Analysis;
517 NCDs–Non communicable Diseases; SD–Standard Deviations; VIF–Variance Inflation Factor;
518 WC–Waist Circumference; WHCR–Waist–to–Hip Circumference Ratio; WHO–World Health
519 Organization 520

521

522

523

524

525	6. References

526	1.	LNeuhouser M. The Importance of Healthy Dietary Patterns in Chronic Disease

527	Prevention. *Nutrition Research* (2019) 70(Commentary):3-6.

528	2.	Tapsell LC, Neale EP, Probst Y. Dietary Patterns and Cardiovascular Disease: Insights and

529	Challenges	for	Considering	Food	Groups	and	Nutrient	Sources.	(2019).

| 530 | [Https://Doi.Org/10.1007/S11883-019-0770-1.](https://doi.org/10.1007/S11883-019-0770-1) *Curr Atheroscler Rep* (2019) 21(9):1-8. |
| --- | --- |
| 531 | 3. Ojo O. Nutrition and Chronic Conditions. *Nutrients* (2019) 11(2):459. |
| 532 | 4. World Health Organisation (WHO). Noncommunicable Diseases Geneva, |
| 533 | Switherland(2018) [cited 2021 Jan 27]. |
| 534 | 5. Gouda HN, Charlson F, Sorsdahl K, Ahmadzada S, Ferrari AJ, Erskine H, et al. Burden of |
| 535 | Non-Communicable Diseases in Sub-Saharan Africa, 1990–2017: Results from the Global Burden |
| 536 | of Disease Study 2017. *The lancet Global Health* (2019) 7(10):1375-E87. |
| 537 | 6. World Health Organization (WHO). *Global Status Report on Noncommunicable Diseases* |
| 538 | *2010*. Geneva: World Health Organization (2011). p. 162. |
| 539 | 7. Santaguida P, Balion C, Hunt D, Morrison K, Gerstein H, P Raina LB, et al. Diagnosis, |
| 540 | Prognosis, and Treatment of Impaired Glucose Tolerance and Impaired Fasting Glucose: |
| 541 | Summary. *AHRQ Evidence Report Summaries* (2005). |
| 542 | 8. Dhawan D, Sharma S. Abdominal Obesity, Adipokines and Non-Communicable Diseases. |
| 543 | *The Journal of steroid biochemistry and molecular biology* (2020) 203:105737. |
| 544 | 9. GBD 2017 Diet Collaborators. Health Effects of Dietary Risks in 195 Countries, 1990– |
| 545 | 2017: A Systematic Analysis for the Global Burden of Disease Study 2017. *The Lancet* (2019) |
| 546 | 393(10184):1958-72. |
| 547 | 10. Aleksandrova K, Giuseppe Rd. Diet, Healthy Aging, and Cognitive Function. *Analysis in* |
| 548 | *Nutrition Research*. Springer (2019). |
| 549 | 11. Abdi H, Williams LJ. Principal Component Analysis. *Wiley interdisciplinary reviews:* |
| 550 | *computational statistics* (2010) 2(4):433-59. |
| 551 | 12. Agnoli C, Krogh V. Dietary Pattern Analysis. *Analysis in Nutrition Research*. Springer |
| 552 | (2019). |
| 553 | 13. Abebe SM, Andargie G, Shimeka A, Alemu K, Kebede Y, Wubeshet M, et al. The |
| 554 | Prevalence of Non-Communicable Diseases in Northwest Ethiopia: Survey of Dabat Health and |
| 555 | Demographic Surveillance System *BMJ Open* (2017) 7(10):1-7. |
| 556 | 14. Misganaw A, Mariam DH, Ali A, Araya T. Epidemiology of Major Non-Communicable |
| 557 | Diseases in Ethiopia: A Systematic Review. *J Health Popul Nutr* (2014) 32 (1):1-13. |
| 558 | 15. Kassie AM, Abate BB, Kassaw MW. Prevalence of Overweight/Obesity among the Adult |
| 559 | Population in Ethiopia: A Systematic Review and Meta-Analysis. *BMJ Open* (2020) |
| 560 | 10(8):e039200. doi: 10.1136/bmjopen-2020-039200. |
| 561 | 16. Molla MD, Wolde HF, Atnafu A. Magnitude of Central Obesity and Its Associated Factors |
| 562 | among Adults in Urban Areas of Northwest Ethiopia. *Diabetes, Metabolic Syndrome and Obesity:* |
| 563 | *Targets and Therapy* (2020) 13:4169-78. |
| 564 | 17. Chooi YC, Ding C, Magkos F. The Epidemiology of Obesity. *Metabolism* (2019) 92:6-10. |
| 565 | doi: <https://doi.org/10.1016/j.metabol.2018.09.005>. |
| 566 | 18. Bastien M, Poirier P, Lemieux I, Després Jp. Overview of Epidemiology and Contribution |
| 567 | of Obesity to Cardiovascular Disease. Prog Cardiovasc Dis. 2014;56:369–81. |
| 568 | 19. Gabir, Mm, Hanson, Rl, Dabelea, D. The 1997 American Diabetes Association and 1999 |
| 569 | World Health Organization Criteria for Hyperglycemia in the Diagnosis and Prediction of |
| 570 | Diabetes. Diabetes Care 2000; 23: 1108–1112. |

| 571 | 20. Shaw, Je, Zimmet, Pz, De Courten, M. Impaired Fasting Glucose or Impaired Glucose |
| --- | --- |
| 572 | Tolerance. What Best Predicts Future Diabetes in Mauritius? Diabetes Care 1999; 22: 399–402. |
| 573 | 21. Forouhi NG, Balkau B, Borch-Johnsen K, Dekker J, Glumer C, Qiao Q, et al. The |
| 574 | Threshold for Diagnosing Impaired Fasting Glucose: A Position Statement by the European |
| 575 | Diabetes Epidemiology Group. *Diabetologia* (2006) 49(5):822-7. doi: 10.1007/s00125-006-0189- |
| 576 | 4. |
| 577 | 22. Hosseini-Esfahani F, Koochakpoor G, Daneshpour MS, Sedaghati-khayat B, Mirmiran P, |
| 578 | Azizi F. Mediterranean Dietary Pattern Adherence Modify the Association between Fto Genetic |
| 579 | Variations and Obesity Phenotypes. *Nutrients* (2017) 9(10):1064. |
| 580 | 23. L.Strate L, R.Keeley B, YinCao, KanaWu, L.Giovannucci E, T.Chan A. Western Dietary |
| 581 | Pattern Increases, and Prudent Dietary Pattern Decreases, Risk of Incident Diverticulitis in a |
| 582 | Prospective Cohort Study. *Gastroenterology* (2017) 152(5):1023-30 e2. |
| 583 | 24. Li M, Shi Z. Dietary Pattern During 1991–2011 and Its Association with Cardio Metabolic |
| 584 | Risks in Chinese Adults: The China Health and Nutrition Survey. *Nutrients* (2017) 9(11):1218. |
| 585 | 25. Mu M, Xu L-F, Hu D, Wu J, Bai M-J. Dietary Patterns and Overweight/Obesity: A Review |
| 586 | Article. *Iran J Public Health* (2017) 46(7):869-76. |
| 587 | 26. Tesfaye T, Zeleke TM, Alemu W, Argaw D, Bedane TK. Dietary Diversity and Physical |
| 588 | Activity as a Risk Factors of Abdomenal Obesity among Adults in Dilla Town, Ethiopia. *PLOs* |
| 589 | *One* (2020) 15(7):e0236671. |
| 590 | 27. Ghachem A, Brochu M, Dionne IJ. Differential Clusters of Modifiable Risk Factors for |
| 591 | Impaired Fasting Glucose Versus Impaired Glucose Tolerance in Adults 50 Years of Age and |

592	Older. *Ther	Adv	Chronic	Dis*	(2019)	10(eCollection). doi: 593 <https://doi.org/10.1177/2040622319854239>.

594 28. Amini M, Esmaillzadeh A, Shafaizadeh S, Behrooz J, Zare M. Relationship between Major

595 Dietary Pattern and Metabolic Syndrome among Individuals with Impaired Blood Glucose. 596 *Nutrition* (2010) 26(6):986-92.

597 29. World Health organization. Global Physical Activity Questionnaire (Gpaq) Analysis

598 Guide. Geneva: WHO, (2011) 1.

599 30. Regassa IF, Endris BS, Habtemariam E, Hassen HY, Ghebreyesus SH. Development and 600 Validation of Food Frequency Questionnaire for Food and Nutrient Intakes of Adults in Butajira, 601 Southern Ethiopia. *Journal of nutritional science* (2021) 10.

602 31. George D, Mallery P. Reliability Analysis. *Ibm Spss Statistics 25 Step by Step*. Routledge 603 (2018). p. 249-60.

604 32. Perini TA, Oliveira GL OJ, Oliveira FP.,. Technical Error of Measurement in 605 Anthropometry. ( 2005) 11:81-5.

606 33. Oumer A, Abraham M, Nuri A. Predictors of Major Dietary Patterns among Pregnant 607 Women Attending Public Health Facilities in Eastern Ethiopia: A New Epidemiological Approach. 608 *Frontiers in Nutrition* (2022) 9. doi: 10.3389/fnut.2022.855149.

609 34. Oumer A, Abraham M, Nuri A. Predictors of Major Dietary Patterns among Pregnant 610 Women Attending Public Health Faility in Eastern Ethiopia: A New Epidemiological Approach. 611 *Frontier in Nutrition* (2022) Accepted Manuscript.

612 35. Plaza-Díaz J, Molina-Montes E, Soto-Méndez MJ, Madrigal C, Hernández-Ruiz Á, Valero 613 T, et al. Clustering of Dietary Patterns and Lifestyles among Spanish Children in the Esnupi Study 614 †. *Nutrients* (2020) 12(9):2536.

615 36. Hu FB. Dietary Pattern Analysis: A New Direction in Nutritional Epidemiology. *Current*

616 *opinion in lipidology* (2002) 13(1):3-9.

617 37. Genuth S, Alberti KG, Bennett P, Buse J, Defronzo R, Hahn R, et al. Expert Committee on 618	the Diagnosis and Classification of Diabetes Mellitus: Follow-up Report on the Diagnosis of	619 Diabetes Mellitus. Diabetes Care 26:3160–3167. (2003).

620 38. Ashwell M, Gibson S. Waist to Height Ratio Is a Simple and Effective Obesity Screening 621 Tool for Cardiovascular Risk Factors: Analysis of Data from the British National Diet and 622 Nutrition Survey of Adults Aged 19–64 Years. *Obesity facts* (2009) 2(2):97-103.

623 39. Association GAotWM. World Medical Association Declaration of Helsinki: Ethical 624 Principles for Medical Research Involving Human Subjects. *Am J Dent* (2014) 81(3):14-8.

625 40. Ethipian Public health	institute (EPHI). *Steps	Survey on	Risk Factors	for	Non*	626 *Communicable Diseases and Prevlence of Selected Ncds Ethiopia.* In: Nutrition, editor. Addis	627 Ababa: EPHI (2016). p. 2-25.

628 41. Peters R, Ee N, Peters J, Beckett N, Booth A, Rockwood K, et al. Common Risk Factors 629 for Major Noncommunicable Disease, a Systematic Overview of Reviews and Commentary: The 630 Implied Potential for Targeted Risk Reduction. *Therapeutic advances in chronic disease* (2019) 631 10:2040622319880392.

632 42. Mudie K, Jin MM, Kendall	L, Addo	J, dos-Santos-Silva I, Quint	J, et	al. Non- 633	Communicable Diseases in Sub-Saharan Africa: A Scoping Review of Large Cohort Studies. 634 *Journal of global health* (2019) 9(2).

635 43. Tesfaye T, Shikur B, Shimels T, Firdu N. Prevalence and Factors Associated with Diabetes 636	Mellitus and Impaired Fasting Glucose Level among Members of Federal Police Commission	637 Residing in Addis	Ababa, Ethiopia. *BMC	Endocrine	Disorders*	(2016)	16(1):68. doi: 638 10.1186/s12902-016-0150-6.

639 44. Worede A, Alemu S, Gelaw YA, Abebe M. The Prevalence of Impaired Fasting Glucose 640 and Undiagnosed Diabetes Mellitus and Associated Risk Factors among Adults Living in a Rural 641 Koladiba Town, Northwest Ethiopia. *BMC Res Notes* (2017) 10(1):251.

642 45. Tekalegn Y, Solomon D, Sahiledengle B, Assefa T, Negash W, Tahir A, et al. Prevalence 643 of Central Obesity and Its Associated Risk Factors among Adults in Southeast Ethiopia: A 644 Community-Based Cross-Sectional Study. *PloS one* (2022) 17(8):e0265107.

645 46. Owolabi EO, Ter Goon D, Adeniyi OV. Central Obesity and Normal-Weight Central	646 Obesity among Adults Attending Healthcare Facilities in Buffalo City Metropolitan Municipality, 647	South Africa: A Cross-Sectional Study. *Journal of Health, Population and Nutrition* (2017)	648 36(1):54. doi: 10.1186/s41043-017-0133-x.

649 47. Herrmann SD, Willis EA, Honas JJ, Lee J, Washburn RA, Donnelly JE. Energy Intake, 650 Nonexercise Physical Activity, and Weight Loss in Responders and Nonresponders: The Midwest 651 Exercise Trial 2. *Obesity* (2015) 23(8):1539-49.

652 48. Melanson E. The Effect of Exercise on Non‐ Exercise Physical Activity and Sedentary 653 Behavior in Adults. *Obesity Reviews* (2017) 18:40-9.

654 49. Mazidi M, Kengne AP, Mikhailidis DP, Toth PP, Ray KK, Banach M. Dietary Food 655 Patterns and Glucose/Insulin Homeostasis: A Cross-Sectional Study Involving 24,182 Adult 656 Americans. *Lipids in health and disease* (2017) 16(1):192. doi: 10.1186/s12944-017-0571-x.

657 50. Bekele TH, De Vries JJ, Trijsburg L, Feskens E, Covic N, Kennedy G, et al. Methodology 658 for Developing and Evaluating Food-Based Dietary Guidelines and a Healthy Eating Index for 659 Ethiopia: A Study Protocol. *BMJ Open* (2019) 9(7):e027846.

660 51. Paradis, Am., Godin, G., Pérusse, L. Et Al. Associations between Dietary Patterns and 661 Obesity Phenotypes. Int J Obes 33, 1419–1426 (2009). [Https://Doi.Org/10.1038/Ijo.2009.179](https://doi.org/10.1038/Ijo.2009.179).

662 52. Barker DJ, Thornburg KL. Placental Programming of Chronic Diseases, Cancer and 663 Lifespan: A Review. *Placenta* (2013) 34(10):841-5.

664 53. Hlaing HH, Liabsuetrakul T. Dietary Intake, Food Pattern, and Abnormal Blood Glucose 665 Status of Middle-Aged Adults: A Cross-Sectional Community-Based Study in Myanmar. *Food* 666 *Nutr Res* ( 2016) 60::1-8.

667 54. Maghsoudi Z, Azadbakht L. How Dietary Patterns Could Have a Role in Prevention, 668 Progression, or Management of Diabetes Mellitus? Review on the Current Evidence. *J Res Med* 669 *Sci* (2020) 17(7):694–709.

670 55. Zhang X, Li Y, Liu a, Zhang Q, Hu X, S. Et Al. (2012)Dietary Pattern and Its Association 671 with the Prevalence of Obesity and Related Cardiometabolic Risk Factors among Chinese Childre 672 Dietary Pattern and Its Association with the Prevalence of Obesity and Related Cardiometabolic 673 Risk Factors among Chinese Children. Plos One 7(8): E43183.

| 674 | [Https://Doi.Org/10.1371/Journal.Pone.0043183](https://doi.org/10.1371/Journal.Pone.0043183). |
| --- | --- |
| 675 | 56. Morales ME, Berkowitz SA. The Relationship between Food Insecurity, Dietary Patterns, |
| 676 | and Obesity. *Current Nutrition Reports* (2016) 5(1):54-60. doi: 10.1007/s13668-016-0153-y. |
| 677 | 57. Seifu CN, Fahey PP, Hailemariam TG, Frost SA, Atlantis E. Dietary Patterns Associated |
| 678 | with Obesity Outcomes in Adults: An Umbrella Review of Systematic Reviews. *Public Health* |
| 679 | *Nutrition* (2021) 24(18):6390-414. |

680

[Click here to access/download;Figure;Figure 1.tiff](https://www.editorialmanager.com/pone/download.aspx?id=32603002&amp;guid=03530997-5b12-419c-b0b6-f2ea3786c648&amp;scheme=1)

[Click here to access/download](https://www.editorialmanager.com/pone/download.aspx?id=32603003&amp;guid=f58f2d47-9ccf-4dcc-b905-4e8bf2f57033&amp;scheme=1)

[**Supporting Information** S3 File.docx](https://www.editorialmanager.com/pone/download.aspx?id=32603003&amp;guid=f58f2d47-9ccf-4dcc-b905-4e8bf2f57033&amp;scheme=1)

[Click here to access/download](https://www.editorialmanager.com/pone/download.aspx?id=32603004&amp;guid=825846f1-900a-4fbd-b6ec-fef5309cc17c&amp;scheme=1)

[**Supporting Information** S1 File.docx](https://www.editorialmanager.com/pone/download.aspx?id=32603004&amp;guid=825846f1-900a-4fbd-b6ec-fef5309cc17c&amp;scheme=1)

[Click here to access/download](https://www.editorialmanager.com/pone/download.aspx?id=32603005&amp;guid=6769ed3b-21de-4eb5-a0e4-49db07ed460a&amp;scheme=1)

[**Supporting Information** S2 File.docx](https://www.editorialmanager.com/pone/download.aspx?id=32603005&amp;guid=6769ed3b-21de-4eb5-a0e4-49db07ed460a&amp;scheme=1)

Revised Manuscript with Track Changes

1 Major dietary patterns of community dwelling adults and their associations with

**Formatted:** Numbering: Continuous

2 impaired blood glucose and central obesity in Eastern Ethiopia: diet-disease
3 ~~exploration~~ epidemiological study
4 Berhanu Abebaw^1^, Abdu Oumer^2*^, Ahmed Ale^3^, ~~and~~ Aragaw Hamza^4^, Imam Dagne^2^, Abdurezak
5 Adem Umer^2^, Dilnessa Fentie^3^, Muluken Yigezu^2^, Zerihun Tariku^2^, and Shambel Abate^2^

**Formatted:** Superscript

6 1 Department of Nutrition and dietetics, School of Public Health, Bahir Dar University, Ethiopia
7 ^2^Department of Public Health, College of Medicine and Health Sciences, Dire Dawa University,
8 Dire Dawa, Ethiopia
9 ^3^School of Medicine, College of Medicine and Health Sciences, Dire Dawa University, Dire
10 Dawa, Ethiopia
11 ^4^Department of Anesthesia, College of Medicine and Health Sciences, Dire Dawa University,
12 Dire Dawa, Ethiopia
13 *Corresponding author:* Abdu Oumer; department of Public Health, College of Medicine and
14 Health Sciences, Dire Dawa University, Dire Dawa, Ethiopia; Email: [omab2320@gmail.com](mailto:omab2320@gmail.com)
15 Abstract
16 Backgrounds: Unhealthy dietary intake is an important preventable risk factor for obesity and
17 impaired blood glucose (IBG), ultimately increasing the risk of non-communicable diseases.
18 When compared to individual food intakes, dietary patterns are a stronger predictor of health
19 outcomes and should be systematically evaluated where such evidence is lacking. This study
20 evaluated dietary patterns and their association with the risk of central obesity and IBG among
21 adults.
22 Methods: A community-based survey was conducted among 501 randomly-selected adults from
23 Eastern Ethiopia. Data was collected using a semi-structured questionnaire during a face-to-face
24 interview that included sociodemographic and lifestyle factors, as well as a validated 89-item food
25 frequency questionnaire (collected over one month). Principal component analysis was used to
26 derive the dietary pattern. While central obesity was assessed using waist and/or hip circumference
27 measurements, fasting blood sugar was used for IBG. A multivariable logistic regression model
28 was fitted with an odds ratio, 95% confidence intervals, and p-values reported.
29 Results: A total of 501 adults (95.3%) were interviewed, with a mean age of 41 years (±12). Five
30 major dietary patterns explaining 71% of the total variance were identified: “nutrient-dense foods”,
31 “high fat and protein”, “processed foods”, “alcohol drinks”, and “cereal diets”. While 20.4% 32	(17.0—24.2%) had IBG, 14.6% (11.8—17.9) were centrally obese, and 94.6% (92.3—96.3) had
33 an increased ~~risk for cardiovascular complications~~waist-to-hip circumference ratio. Central obesity
34 is associated with upper wealth status (AOR = 6.92; 2.91—16.5), physical inactivity (AOR = 21.1;
35 2.77—161.4), a diet high in nutrient-dense foods (AOR = 1.75; 0.75—4.06), processed foods	36	(AOR = 1.41; 0.57—3.48), and cereal diets (AOR = 4.06; 1.87—8.82). The risk of IBG was
37 associated with upper wealth status (AOR = 2.36; 1.36—4.10), physical inactivity (AOR = 2.17;
38 0.91—5.18), upper tercile of nutrient-dense foods (AOR = 1.35; 0.62—2.93), fat and protein diet 39	(AOR = 1.31; 0.66—2.62), and cereal diet consumption (AOR = 3.87; 1.66—9.02).
40 Conclusion: IBG and central obesity were prevalent and predicted by upper tercile consumption
41 of nutrient-dense foods, high fat and protein diets, processed foods, and cereal diets, which could
42 guide dietary interventions. 43

44	Key words: central obesity; dietary intake; dietary pattern; impaired blood glucose; prediabetes 45

46

47

48

49

50

51

52

53

54

55

56 Major dietary patterns of community dwelling adults and their associations with
57 impaired blood glucose and central obesity in Eastern Ethiopia: diet-disease
58 ~~exploration~~ epidemiolgical study
59 1. Introduction
60 Now a day the lifestyle characteristics of individuals, including dietary intake, change from time
61 to time dramatically. At the same time, the occurrence of non-communicable diseases (NCDs)
62 associated with impaired blood glucose (IBG) and obesity is rising (1). Hence, these are early
63 indicators of increased risk of NCDs and their complications (2, 3). NCDs are responsible for the
64 deaths of 41 million people (71%) each year, with low- and middle-income countries accounting
65 for more than 85% of all deaths. Obesity and high blood glucose kill about 17.9 and 1.6 million
66 people, respectively (4). The burden of NCDs has significantly increased, mainly attributable to
67 dietary habit change, increasing obesity and poor lifestyles as a result of poor lifestyle practices
68 (5). These are becoming major public health problems, especially in developing countries,
69 accounting for an estimated 80% of all mortality (6).
70 Prediabetes or IBG, defined as fasting blood glucose (FBS) of 100-126 mg dl^-1^, is a precursor to
71 established diabetes mellitus, which could be aggravated by insulin resistance due to abdominal
72 obesity (7). In particular, central obesity, with a higher waist circumference (WC) and/or waist-to-
73 hip circumference ratio (WHCR), is the sole cause of many NCDs related morbidity and mortality
74 (8). Among the risk factors, dietary consumption is a long -time exposure and one of the
75 modifiable and preventable exposures for overt NCDs (3), which needs to be investigated
76 systematically (9). Dietary risk factors were responsible for an estimated 11 million (10–12) deaths
77 and 255 million (234–274) DALYs lost; including high sodium intake, (3 million deaths), low
78 intake of whole grains (3 million deaths) and low intake of fruits ( 2 million deaths) (9).
79 Due to the methodological shortcomings of individual food or nutrient analysis for predicting
80 health outcomes, dietary pattern analysis is a better approach to characterize dietary consumption.
81 It is becoming a more practical approach to determine the role of diet in the prevention and
82 treatment of chronic diseases (2), taking into account the confounding, interaction, and synergistic
83 effects of nutrients in food that make causation difficult (10, 11). It helps to evaluate the
84 cumulative and interactive effects of each dietary compound in a food and to consider the
85 complexity of the diet (10, 12).
86 Hence, dietary patterns could be more predictive for IBG and central obesity. Central obesity and
87 IBG are the major risk factors that increase the risk of NCDs, including diabetes mellitus. NCDs
88 are a public health problem in Ethiopia, where 4.2% are victims of diabetes mellitus, causing 70%
89 of overall deaths (13). While an estimated up to 39% are centrally obese, 5% of overall mortality
90 is due to IBG (14). In Ethiopia, an estimated 20.7% and 6.9% of adults are overweight and obese
91 (15), where 35 to 38% of adults have central obesity (16). This huge burden of central obesity, is
92 one main risk factor for established NCDs and all their complications (17, 18). For instance, an
93 estimated 25% of IBG will progress to diabetes while 50% remain in an impaired state (19, 20)
94 and increases cardiovascular mortality by 43% (21). The rate of progression to visible diseases is
95 rapid, necessitating a targeted intervention to slow progression.
96 Given the huge role of diet in the development of central obesity and IBG, there is a need to
97 investigate the link in the absence of such evidence in Ethiopia. The study area is characterized by
98 physical access to diversified diet from agricultural productivity, industrial processing and imports.
99 Studies conducted in other countries showed a clear link between dietary patterns and the risk of
100 central obesity and IBD (22-25). However, evidence from other countries may not be applicable
101 to our case, where dietary habits greatly vary even from region-to-region. In addition, studies
102 conducted so far mainly focus on individual food consumptions (13, 16, 26, 27), which cannot be
103 powerful predictors. This would help to design appropriate context-specific dietary strategies to
104 decrease the risk of central obesity and IBG in Ethiopia. This study was to evaluate the association
105 between major dietary patterns and central obesity and IBG among adults in Ethiopia.
106 2. Materials and Methods
107 2.1.	Study Area and Period
108 This study was conducted in Dire Dawa and Harar, located in the eastern parts of Ethiopia. Harar
109 is located 526 kilometers from the capital city, Addis Ababa. Dire Dawa has an estimated
110 population of more than 0.5 million, while more than 0.3 million people reside in the Harari
111 regions, where more than half of the population resides in urban areas. According to the 2010
112 Harari Region population projection, there are 250, 093 with 146, 913 living in the urban areas
113 and 122, 942 are males, with a total household of 64, 334. These two regions have a multiethnic
114 and diverse population with a heterogenous agricultural productivity for fruits, vegetables,
115 sorghum, maize, wheat, and other cereals. Furthermore, being an industrial center for many
116 manufacturing industries and a trade center closest to neighboring countries makes access to
117 imported foods easier, in addition to domestic production. Hence, rice, white flour, macaroni, and
118 other industrially processed foods are accessible, in addition to cereals and grains marketed from
119 other regions of the country. The study was conducted from September 10 to November 06, 2021.
120 The study period was considered the fasting and feasting period to capture habitual	Cereals,
121 including teff, wheat, and legumes, were the most staple crops in the study areas. In addition, some
122 processed foods, potatoes, and other fruits are the major foods consumed in the study area.
123 2.2.	Study design and population
124 A community-based ~~survey~~ cross-sectional study was conducted among 501 randomly selected
125 adults (aged >= 18 years) from the two study sites available during the study period. Hence, the
126 findings of this study would be generalized to all adults residing in Harar and Dire Dawa towns,
127 Eastern Ethiopia. Adults who were pregnant, had abdominal swelling (ascites), or had detectable
128 thoracic and lumbar spinal deformity were excluded because waist circumference (WC)
129 measurements are biased. Also, clients with serious psychiatric disorders, which make them unable
130 to give oriented responses and unable to communicate, were excluded. More importantly, adults
131 with clinically diagnosed diabetes and/or known diabetes patients on treatment were also excluded
132 from the study.
133 2.3.	Sample size determination and sampling procedures
134 The minimum sample size for this study was estimated using a sample size formula for single
135 proportion with ”P” as the prevalence of central obesity from the previous study at 24.4% (26), at
136 a 95% confidence level, a “Z” critical value of 1.96, and a marginal error of “d” of 5%. The sample
137 size became 145. The sample size for the association of dietary pattern with central obesity and
138 IBG was calculated using the StatCalc module in Epi Info software version 7.0. After checking for
139 many risk factors, the maximum sample size calculated was taken considering a prudent dietary
140 pattern for predicting IBG (AOR = 2.26), abnormal fasting blood glucose level (OR = 2.26),
141 percentage of IBG among those with a healthy dietary pattern (24%) (28), power of 80%, and
142 equal sample of potential exposed and unexposed groups. ~~a ratio of exposed and unexposed as~~
143 ~~one.~~ Thus, taking dietary pattern as a risk factor for IBG and central obesity, the sample size
144 became 504. After adding a non-response rate of 5%, the final minimum sample for this study was 145	526.
146 Stratified random sampling combined with simple and systematic random sampling was employed
147 to select study participants. The total sample size was proportionally allocated to the two study
148 sites (Dire Dawa and Harar) and to the smaller study units consecutively. A random sample of four
149 districts in Harar and four kebeles (fourth administrative division level in Ethiopia) were randomly
150 selected, where households were selected using systematic random sampling at a skipping interval.
151 The sample interval (K) was calculated by dividing the total number of households by the allocated
152 sample size for each kebele. Then, a random sample of adults residing in the selected household
153 was selected and interviewed. The first household was selected randomly, and adults residing in
154 randomly selected households were included in the study.
155 2.4.	Data collection
156 Data were collected through a face-to-face interview using a set of pretested and structured
157 questionnaires, as well as standardized anthropometric measurements and FBS measurement. The
158 tool contains socio-demographic variables, lifestyle-related questions, and a validated semi-
159 quantitative Food Frequency Questionnaire (FFQ) to capture the dietary intake of adults over the
160 last one month. Data was collected by trained health care workers and graduating health students
161 through face-to-face interviews, measurements, and laboratory analysis. Data collectors got the
162 data by interviewing the study subject and probing for possible missed food consumed during the
163 specific period of time.
164 The 16-item validated General Physical Activity Questionnaire (GPAQ) tool developed by WHO
165 for physical activity surveillance was employed to assess the level of physical activity through
166 face-to-face interviews. The activity level of the study participants was evaluated according to the
167 standard WHO total physical activity calculation guide, and the level of total physical activity was
168 categorized as physically active or inactive as detailed in the previous section. After calculating
169 the metabolic equivalent per week (MET/week), a value below 600 was used to indicate physical
170 inactivity (29).
171 A modified semi-quantitative Helen Keller FFQ containing 89 food items with about nine food
172 groups was used to collect the dietary intake of study subjects. The FFQ was asked for the food
173 that the subject consumed over the past 1 month to capture reliable and habitual dietary
174 consumption of the individual. We further contextualized the list of food items for the particular
175 context by considering the local foods commonly produced and consumed **(Supplementary file**
176 **1).** The FFQ validated for the Ethiopian adults has been contextualized and pretested to capture
177 the dietary intake of adults. A validation study showed that the contextualized tool was valid and
178 reliable in measuring the dietary micro- and macronutrient intake of adults in Ethiopia (30). In
179 pilot analysis, the FFQ tool we assed was found to be highly reliable with a Cronbach’s alpha value
180 above 0.86, indicative of a reproducible tool (31).
181 For assessment of central obesity, WC and hip circumference (HC) measurements were taken by
182 trained anthropometric measurers using a non-stretchable tape meter at the end of a gentle
183 expiration. The clothing and jewelry of the clients will be kept minimal. The WC was measured at
184 a point midway between the lowest rib and the iliac crest in a horizontal plane at around the
185 umbilicus while the respondents were instructed to breath gently. The tape meter is not tightly
186 secured to avoid pressure and bias in measurement. While the HC was measured at the maximum
187 circumference of the pelvis over the great trochanters, Measurements were made at least twice,
188 and the average of the two measurements was recorded.
189 The laboratory analysis for blood glucose measurement was done by qualified and trained health
190 professionals. The blood glucose measurement was done using a 2 mL capillary blood sample
191 collected from the finger tips, and analyzed using a simple yet valid glucometer machine and
192 recorded as mg dl^-1^. A specific cut off point from 100 to 125 mg dl-1 was used to diagnose IBG or
193 prediabetes.
194 2.5.	Data quality assurance
195 A pair of trained data collectors were deployed to collect the data from study subjects, as
196 anthropometry needs	curious	measurements. Training	was	given	on	appropriate interview
197 techniques and anthropometric measurements on WC and HC. Anthropometric reliability was
198 assessed on 10 study subjects during the pretest, and intra~~er~~- and interobserver variations were
199 calculated. Data collectors with high inter and both intra-observer bias (technical error of
200 measurement above 2%) were retrained and collected the data once it was in the acceptable range
201 (32). All standard measuring procedures and instruments were strictly followed during data
202 collection. The standard operating procedure for blood sampling and glucose testing was followed
203 accordingly. During data entry into EpiData, the data quality was maintained by making legal
204 ranges, skipping patterns, appropriate coding, and careful data entry. A Piot study was conducted
205 on the unselected study area in order to test and correct the necessary corrections. The FFQ is
206 adapted and modified with the purpose of not missing relevant foods that are commonly consumed
207 in the study area (33).
208 2.6.	Data analysis
209 After checking for completeness and inconsistencies, the collected data was entered into EpiData
210 Version 3.02 and exported to SPSS for analysis. All statistical analyses were carried out with
211 Microsoft Excel 2016 and IBM SPSS Statistics for Windows, Version 20.0 (IBM Corp., Armonk,
212 NY, USA). Descriptive statistics were computed for each variable, and the data was presented in
213 statistical tables and graphs. The WHCR was calculated from WC and HC and checked for
214 normality using the Kolmogorov-Smirnov test. Principal component analysis was conducted using
215 household assets to derive the socioeconomic status (wealth index) of adults. All assumptions of
216 PCA were checked using the Kaiser Mayer-Olkin (KMO) sampling adequacy, correlations, and
217 covariance parameters. Based on the derived factor scores, wealth status was categorized into five
218 quartiles, from the poorest to the wealthiest.
219 Dietary pattern analysis
220 Exploratory factor analysis using a principal component analysis was used to derive dietary
221 patterns using the average frequency of 89-item food consumption in the past one month. The
222 consumption frequency was recategorized into daily equivalents based on previous literature (34)
223 and weighted daily consumption equivalents (**Supplementary File 2**). In addition, some closely
224 related food items were summed up and recategorized into a single food item as needed. Bartlett’s
225 test of sphericity and the KMO measure of sampling adequacy were used to verify the
226 appropriateness of exploratory factor analysis. For the degree of intercorrelations between
227 variables, we used a KMO value of greater than 0.5 and a p-value of less than 0.05 for the Bartlett
228 test of sphericity. Orthogonally rotation using the varimax option was used to enhance the
229 interpretability of dietary patterns (35). The principal components were retained based on the
230 following criteria: factor eigenvalue, identification of a break point in the scree plot, the proportion
231 of variance explained, and interpretability of the dietary patterns (12, 36). A rotated factor loading
232 matrix was used to assess the direction and strength of correlation between each food item and its
233 components. We also checked for a complex association between one food items and multiple
234 factors, where such factors were removed. The corresponding factor score for each dietary pattern
235 was constructed by summing the observed daily frequency of consumption of individual food
236 items weighted by the factor loading. Hence, a high score of a component indicates more frequent
237 consumption of the foods constituting that particular dietary pattern. For further analysis, each
238 factor score was ranked into three terciles as low, medium, and high frequency of consumption.
239 Dietary pattern with central obesity and IBG
240 A separate multivariable logistic regression model was fitted to evaluate the association between
241 dietary pattern and central obesity and IBG. Both crude and adjusted odds ratios with 95%
242 confidence intervals and/or p-values were ~~reported~~computed. Multicollinearity was checked using
243 the variance inflation factor (VIF) and/or the standard error estimates. The model fitness was
244 checked using Hosmer and Lemeshow’s goodness of fit. In addition, variables with a p-value
245 below 0.2 in bivariable logistic regression and biologically plausible risk factors for the outcomes
246 were considered in the multivariable logistic models. Statistical significance was declared at a p-
247 value below 0.05.
248 2.7.	Variables of the study
249 While the dependent variables of this study were IBG and central obesity, the independent
250 variables were sociodemographic variables (age, sex, income, occupation, and education), physical
251 activity, family history of diabetes, dietary pattern, dietary intake of food groups, and habits of
252 substance use. The outcome variable IBG was categorized as IBG when the FBS between 100 and
253 125 mg dl^-1^ (37). Central obesity was defined as having a waist circumference of 94 cm for men
254 and 80 cm for women, or a WHCR of 0.90 in men and 0.85 in women (38).
255 2.8.	Ethical approval
256 Ethical approval was obtained from Dire Dawa University, Institutional Research Review Board.
257 The support letter was taken to the respective regional health office for cooperation. A written
258 informed consent was obtained from each study participant after explaining the study purpose,
259 confidentiality, and procedures in detail. The study was conducted in accordance with the
260 Declaration of Helsinki for Human Research (39). The data will be used for this research purpose
261 only, and the data will be kept confidential. Respondents identified with central obesity and IBG
262 got counseling, recommendation, and/or linkage to the health facilities. All the COVID-19
263 standard prevention precautions were implemented throughout the data collection process.
266 3. Results
267 3.1.	Sociodemographic characteristics
268 A total of 501 study subjects were included in the current survey, with a response rate of 95.3%.
269 More than half (66.7%) of the participants were females. The age of the respondents ranges from
270 18 –~~—~~64 years, with a mean age of 41 years (±12). ~~Almost all~~The majority, (90%) of the
271 respondents reside with their families. A total of 203 (40.5%) attended primary school, and 143
272 (28.5%) and 127 (25.3%) were housewives and government employees, respectively. About 188
273 (37.5%) and 44 (8.8%) were in the wealthier and wealthiest ranks regarding socioeconomic status.
274 On the other hand, 105 (21%) and 100 (20%) of the respondents were from the poorest and poorer
275 households, respectively (Table 1).
276 **Table 1.** sociodemographic characteristics of respondents for a dietary pattern study in Dire Dawa
277 and Harar, eastern Ethiopia

| Variables | Categories | Frequency | Percent |
| --- | --- | --- | --- |
| Sex | Male | 182 | 33.3 |
|  | Female | 319 | 66.7 |
| Live with | With family | 450 | 90 |
|  | Friends | 28 | 5.6 |
|  | Lonely | 21 | 4.4 |
| Education | Illiterate | 24 | 4.6 |
|  | Primary education | 203 | 40.5 |
|  | Secondary education | 107 | 21.4 |
|  | College and above | 167 | 33.3 |
| Marital status | Married | 409 | 81.6 |
|  | Single | 71 | 14.2 |
|  | Widowed | 21 | 4.2 |
| Occupation | Daily laborer | 18 | 3.6 |
|  | Farmer | 9 | 1.8 |
|  | Government employee | 127 | 25.3 |
|  | Housewife | 143 | 28.5 |
|  | Merchant | 69 | 13.8 |
|  | Private employed | 61 | 12.2 |

|  | Others | 74 | 14.8 |
| --- | --- | --- | --- |
| Wealth index | Poorest | 105 | 21.0 |
|  | Poor | 100 | 20.0 |
|  | Medium | 64 | 12.8 |
|  | Wealthier | 188 | 37.5 |
|  | Wealthiest | 44 | 8.8 |

278 3.2.	Physical activity and substance use related factors
279 Regarding the physical activity of adults, ~~almost~~ more than three-fourths (383, 76.8%) of them
280 were reported to be physically inactive with a median metabolic equivalent of 240 MET (IQR: 0-
281 500 MET). While only 55 (11%) had a regular physical activity schedule, 85 (27.9%) had a family
282 history of diabetes mellitus. Approximately 304 (60.7%) reported chewing khat, 101 (20.2%)
283 reported drinking alcohol, and 24 (4.8%) reported chewing khat and smoking cigarettes.
284 3.3.	Dietary patterns of adults
285 A total of 465 (98%) of adults had a usual three-times-a-day meal, while 478 (98.4%) were
286 reported to have a regular meal. Almost all (99.4%) reported using liquid oils for cooking. About
287 95 (19%) and 48 (9.6%) reported having regular sweet and soda drinks and fatty foods,
288 respectively. In terms of fruit and vegetable consumption, only 96 (19.2%) reported regular fruit
289 consumption and 140 (27.9%) reported vegetable consumption (at least >= 6 times per week).
290 The consumption frequency was recategorized into daily equivalents based on previous literature
291 and weighted daily consumption equivalents. In addition, some closely related food items were
292 summed up and categorized into a single food item as needed. An exploratory factor analysis using
293 the principal component analysis method was conducted. Food items with a KMO value of below
294 0.30 were removed iteratively. All the assumptions of PCA were checked. The overall sample
295 adequacy was evaluated using KMO, where the p-value was 0.91. Similarly, the presence of
296 interitem correlation was checked using the Bartlett test of sphericity (p-value 0.0001), indicating
297 the presence of a statistically significant correlation. Furthermore, items with a communality below
298 0.30 were removed step-by-step. The final food items to be included were determined by the
299 following criteria: interpretability of the factors, improving the total variance explained, absence
300 of complex structure evidenced by rotation component matrix, eigen value, and scree plots
301 (**Supplementary File 3)**.
302
303 Five dietary patterns explaining 71% of the total variance were identified; “Animal source foods,
304 fruits, and vegetables-nutrient dense foods”, “high fat and protein foods”, “processed foods”,
305 “traditional alcoholic drinks”, and “cereal foods”. A total of sixteen, nine, seven, four and four
306 food items constitute the first, second, third, fourth, and fifth dietary patterns as indicted in
307 **Supplementary File 3**. Nutrient dense foods composed of animal source foods, fruits, and
308 vegetables account for the major part of the variance (42%), followed by high fat and protein foods
309 (11.2%). For further analysis, the standard score of each dietary pattern was categorized into three
310 terciles, where the lower score indicates less frequent consumption of foods and is assigned the
311 lowest tercile. Furthermore, traditional alcoholic drinks were retained as they are relevant to central
312 obesity and IBG (**Table 2****)**.
313 **Table 2**. Total variance explained and summary statistics for factor scores for identified dietary
314 patterns in Ethiopia

| S.no | Dietary  patterns | Label | Variance  explained | Median  (Q_2_) | Q_1_ | Q_3_ |
| --- | --- | --- | --- | --- | --- | --- |
| 1 | DP_1 | Animal source foods, | 42% | -0.250 | -0.358 | -0.180 |
|  |  | fruits, and vegetables- |  |  |  |  |
|  |  | Nutrient dense foods |  |  |  |  |
| 2 | DP_2 | High fat and protein | 11.2% | -0.036 | -0.241 | 0.079 |
|  |  | foods |  |  |  |  |
| 3 | DP_3 | Processed foods | 9.0% | -0.460 | -0.762 | 0.667 |
| 4 | DP_4 | Traditional alcoholic | 5.0% | -0.146 | -0.176 | -0.056 |
|  |  | drinks |  |  |  |  |
| 5 | DP_5 | cereal foods | 3.83% | -0.177 | -0.265 | 0.087 |
|  | Total |  | 71.0% | - | - | - |

315 3.4.	Magnitude of central obesity and impaired blood glucose
316 As clearly shown in Figure 1 below, an estimated 20.4% (95% CI: 17.0 –~~—~~24.2%) of the subjects
317 had impaired blood glucose or prediabetes. While 14.6% (95% CI: 11.8—17.9%) of adults had
318 central obesity based on the sex-specific WC cutoff points, 94.6 (95% CI: 92.3 –~~—~~96.3) of them
319 had a WHCR, which makes them at risk for any cardiovascular complications (Figure 1). When
320 disaggregated by sex, females had a higher rate of central obesity by WHCR (98% vs. 86%) and
321 WC (17.2% vs. 9.9%). Moreover, the prevalence of IBG~~G~~ was higher among males (22.5%)
322 compared to females (17.5%). The mean blood glucose level was 180.1 mg dl^-1^ (±50). Similarly,
323 the mean and standard deviation for WC and WHCR were 66.7 (±13.4) and 0.96 (±0.46).
324
325 **Figure 1.** The magnitude of impaired blood glucose, central obesity (based on WC cutoff points
326 of 80 and 94 cm for females and males, a WHC of 0.85 and 0.90 for females and males, b) among
327 adults residing in Dire Dawa and Harar, Eastern Ethiopia.
328 3.5.	Factors associated with central obesity and impaired blood glucose
329 An	exploratory	logistic	regression	was	carried	out	to	identify	the	association between
330 sociodemographic factors and dietary patterns with IBG and central obesity among adults, as
331 detailed in Table 3. Increasing age is associated with an increased ~~risk~~ odds of central obesity and
332 IBG. While males (COR = 1.24; 95% CI: 0.79 –~~—~~1.93) had a 24% higher ~~risk~~ odds of IBG
333 compared to females, females (COR = 1.86; 1.05 –~~—~~3.30) had a significantly higher ~~risk~~
334 occurence of central obesity. The ~~magnitude~~ odds of IBG (COR = 2.18; 95% CI: 1.34 –~~—~~3.53)
335 and central obesity (COR = 9.65; 95% CI: 4.29 –~~—~~21.7) was 2.1 and 9.65 folds higher in the
336 wealthiest versus poorest families, respectively. Similarly, housewives had 38% increased odds of 337	IBG (COR = 1.38; 95% CI: 0.84 –~~—~~2.28) and central obesity (COR = 2.33; 95% CI: 1.34 –~~—~~
338 4.04) compared to adults engaged in private work. The risk of central obesity was found to be high
339 among those with irregular meals (COR = 1.68; 95% CI: 0.60 –~~—~~4.66), physically inactive (COR

340	= 1.54; 95% CI: 0.95 –~~—~~2.503), and habits of sweet food consumption (COR = 1.63; 95% CI: 341	0.91 –~~—~~2.91) (Table 3).

342

343 **Table 3.** Bivariable logistic regression output for determinants of IBG and central obesity among
344 adults from Dire Dawa and Harar, Eastern Ethiopia.

| Factors | Categories | IBG (pre  diabetes) | | COR (95% CI) | Central obesity  (WC) | | COR (95% CI) |
| --- | --- | --- | --- | --- | --- | --- | --- |
|  |  | IBG | No  IBG |  | Yes | No |  |
| Age in years | | –- | –- | 1.01 (0.99-1.03) | –- | –- | 1.03 (1.01-1.1) |
| Sex | Female | 61 | 258 | 1 | 55 | 264 | 1.86 (1.05-3.30)  * |
|  | Male | 41 | 141 | 1.24(0.79-1.93) | 18 | 164 | 1 |
| Wealth index | Poor | 30 | 175 | 1 | 7 | 198 | 1 |
|  | Medium | 9 | 55 | 0.96 (0.43-2.13) | 7 | 57 | 3.47(1.17-10.3) * |
|  | Wealthier | 63 | 169 | 2.18(1.34-3.53) * | 59 | 173 | 9.65(4.29-21.7) * |

| Occupation | Housewife | 35 | 106 | 1.38(.84-2.28) | 34 | 107 | 2.33(1.34-4.04) * |
| --- | --- | --- | --- | --- | --- | --- | --- |
|  | Government | 22 | 105 | 0.88(.50-1.54) | 11 | 116 | 0.69(0.33-1.45) |
|  | Private | 45 | 188 |  | 28 | 205 | 1 |
| Regular  meals | No | 1 | 23 | 1 | 5 | 18 | 1.68(0.60-4.66) |
|  | Yes | 102 | 375 |  | 68 | 410 | 1 |
| Physical activity | Physically  active | 31 | 88 | 1.54(0.95-2.50) | 1 | 118 | 1 |
|  | Physically  inactive | 71 | 311 | 1 | 72 | 310 | 1.54(0.95-2.503) |
| Sweet and soda drinks | No | 75 | 331 | 1 | 54 | 352 | 1 |
|  | Yes | 27 | 68 | 1.75(1.05-2.92) * | 19 | 76 | 1.63(0.91-2.91) |
| Regular physical  activity | No | 94 | 352 | 1.57(0.72-3.43) | 64 | 382 | 0.86(0.40-1.83 |
|  | Yes | 8 | 47 | 1 | 9 | 46 |  |
| Nutrient dense foods (DP-1) | Lowest | 45 | 119 | 1.76(1.05-2.95) * | 27 | 137 | 1 |
|  | Middle | 26 | 136 | 0.89(0.50-1.57) | 6 | 156 | 0.20(0.08-0.49) |
|  | Highest | 31 | 144 | 1 | 40 | 135 | 1.50(0.87-2.59) |
| High fat and protein diet (DP-2) | Lowest | 41 | 126 | 1.70(1.0-2.92) | 33 | 134 | 1.63(0.91-2.94) |
|  | Middle | 34 | 132 | 1.35(0.77-2.35) | 18 | 148 | 0.81(0.42-1.57) |
|  | Highest | 27 | 141 | 1 | 22 | 146 | 1 |
| Processed foods (DP-3) | Lowest | 18 | 148 | 1 | 20 | 146 | 1 |
|  | Middle | 32 | 137 | 1.92(1.03-3.58) * | 21 | 148 | 1.04(0.54-1.99) |
|  | Highest | 52 | 114 | 3.75 (2.08-6.76) * | 32 | 134 | 1.74(0.95-3.20) |
| Alcoholic drinks (DP- 4) | Lowest | 34 | 133 | 1 | 24 | 142 | 1 |
|  | Middle | 31 | 132 | 1.37(0.82-2.28) | 13 | 150 | .51(0.25-1.05) * |
|  | Highest | 37 | 134 | 0.68(0.38-1.20) | 36 | 136 | 1.57 (0.89-2.76) |
| Cereal diets (DP-5) | Lowest | 34 | 133 | 1 | 13 | 154 | 1 |
|  | Middle | 31 | 132 | .092(0.53-1.58) | 17 | 146 | 1.38 (0.65-2.94) |
|  | Highest | 37 | 134 | 1.08(0.64-1.82) | 43 | 128 | 3.98(2.05-7.72) * |

345 *Note: * statistically significant association at p-value <0.05.*
346 Dietary patterns were found to be associated with ~~the risk~~higher odds of central obesity and IBG
347 among adults. Adults who consumed less nutrient dense food (COR = 1.76; 95% CI: 1.05 –~~—~~2.95)
348 and more fat and protein-rich foods (COR = 1.35; 95% CI: 0.77 –~~—~~2.35), for example, had a 1.76-
349 and 1.35-times higher ~~risk~~ magnitude of IBG, respectively. More frequent consumption of
350 processed foods (COR = 3.75; 95% CI: 2.08 –~~—~~6.76) and medium consumption of traditional
351 alcoholic drinks (COR = 1.37; 95% CI: 0.82 –~~—~~2.28) were significant predictors of	IBD.
352 However, optimal consumption of a cereal diet was protective against IBG. Furthermore, eating
353 nutrient-~~—~~dense foods (COR = 1.50; 95% CI: 0.87 –~~—~~2.59) and highly processed foods (COR =
354 1.74; 95% CI: 0.95 –~~—~~3.20) increased the likelihood of central obesity by 50% and 74%,
355 respectively. Frequent consumption of traditional alcoholic drinks (COR = 1.57; 95% CI: 0.89 –
356 ~~—~~2.76) and consumption of cereal diets (COR = 3.98 (2.05 –~~—~~7.72)) was shown to increase the
357 occurrence~~risk~~ of central obesity by ~~1.~~57% and ~~3.~~258%.~~—folds.~~ However, in a dietary pattern
358 characterized by fat and protein-rich foods, the risk for both central obesity (COR = 1.70; 95% CI:
359 1.0 –~~—~~2.92) and IBG (COR = 1.63; 95% CI: 0.91 –~~—~~2.94) was higher among those with a lower
360 tercile (Table 3).
361 A separate multivariable logistic model was fitted in a step-wise backward regression approach
362 for central obesity and IBG. Sex, wealth status, physical activity level, and dietary patterns 1, 3,
363 and 5 were found to be associated with central obesity among adults. Being physically inactive 364	(600 MET) (AOR = 21.1; 95% CI: 2.77 –~~—~~161.4), from a higher socioeconomic class (6.92 (2.91
365 –~~—~~16.5), and females (AOR = 1.67 (0.82—3.40) were positively associated with a higher ~~risk~~
366 occurrence of central obesity. More importantly, those who consumed more nutrient-dense foods 367	(1.75; 95% CI: 0.75 –~~—~~4.06), processed foods (AOR = 1.41 (0.57 –~~—~~3.48), and cereal diets (AOR

368	= 4.06; 95% CI: 1.87 –~~—~~8.82) had a higher ~~risk~~ odds of central obesity. An increasing dose-~~—~~

369 response relationship was observed where odds of its occurrence ~~the risk~~ was~~is~~ higher for more
370 frequent consumption (Table 4).
371 **Table 4.** Adjusted association between dietary patterns and central obesity among adults from Dire
372 Dawa and Harar, Eastern Ethiopia.

| Factors | Categories | Central obesity  (WC) | | AOR (95% CI) | p-value |
| --- | --- | --- | --- | --- | --- |
|  |  | Yes | No |  |  |
| Sex | Female | 55 | 264 | 1.67(0.82-3.40) | 0.157 |
|  | Male | 18 | 164 | 1 |  |
| Wealth index | Poor | 7 | 198 | 1 |  |
|  | Medium | 7 | 57 | 2.56(0.80-8.25* | 0.115 |
|  | Wealthier | 59 | 173 | 6.92(2.91-16.5) | 0.001** |
| Physical activity | Physically active | 1 | 118 | 1 |  |
|  | Physically inactive | 72 | 310 | 21.1(2.77-161.4) | 0.003** |
| Nutrient dense foods (DP-1) | Lowest | 27 | 137 | 1 |  |
|  | Middle | 6 | 156 | 0.28(0.09-0.87) | 0.028* |
|  | Highest | 40 | 135 | 1.75(0.75-4.06) | .193 |
| Processed foods (DP-3) | Lowest | 20 | 146 | 1 |  |
|  | Middle | 21 | 148 | 1.59(0.73-3.46) | 0.246 |
|  | Highest | 32 | 134 | 1.41(0.57-3.48) | 0.456 |
| Cereal diets (DP-5) | Lowest | 13 | 154 | 1 |  |
|  | Middle | 17 | 146 | 1.51(0.66-3.45) | 0.325 |
|  | Highest | 43 | 128 | 4.06(1.87-8.82) * | 0.0001** |

373 Note: statistically significant factors associated with central obesity at p-value <0.05* and 0.001^**^
374 A multivariable logistic regression output was fitted using Hosmer and Lemeshow’s goodness of
375 fit (P-value = 0.847), indicating a fit model. Those from wealthier families (AOR = 2.36; 95% CI:
376 1.36 –~~—~~4.10), those without regular physical activity (AOR = 2.17; 95% CI: 0.91 –~~—~~5.18) and
377 those with sweet food consumption habits (AOR = 2.46; 95% CI: 1.30—4.66) had a 2.36-~~—~~, 2.17-

378 ~~—~~, and 2.46-~~—~~times increased ~~risk~~ odds of having IBG level compared to counterparts. Among
379 the identified dietary patterns, lower consumption of nutrient-dense foods (AOR = 1.35; 0.62 –~~—~~
380 2.93), higher fat and protein diets (AOR = 1.31; 95% CI: 0.66 –~~—~~2.62) and higher cereal diet
381 consumption (AOR = 3.87; 95% CI: 1.66 –~~—~~9.02) were important dietary determinants of IBG
382 level. On the other hand, the ~~risk~~ oddsof IBG was 60% higher among those who were physically 383	active (AOR = 1.60; 95% CI: 0.90–2.85) (Table 5).
384 **Table 5.** Multivariable logistic regression showing the risk factors of IBG among adults residing
385 in Dire Dawa and Harar, Eastern Ethiopia
386

| Factors | Categories | IBG (pre diabetes) | | COR (95% CI) | P-value  387 |
| --- | --- | --- | --- | --- | --- |
|  |  | IBG | No IBG |  |  |
| Wealth index | Poor | 30 | 175 | 1 |  |
|  | Medium | 9 | 55 | 1.29(0.54-3.04) | 0.567388 |
|  | Wealthier | 63 | 169 | 2.36(1.36-4.10) | 0.002* |
| Physical activity | Physically active | 31 | 88 | 1.60(0.90-2.85) | 0.107389 |
|  | Physically inactive | 71 | 311 | 1 |  |
| Sweet and soda drinks | No | 75 | 331 | 1 | 390 |
|  | Yes | 27 | 68 | 2.46(1.30-4.66) | 0.006* |
| Regular physical activity | No | 94 | 352 | 2.17(0.91-5.18) | 391  0.083 |
|  | Yes | 8 | 47 | 1 | 392 |
| Nutrient dense foods (DP-1) | Lowest | 45 | 119 | 1.35(0.62-2.93) | 0.490  393 |
|  | Middle | 26 | 136 | 1.27(0.65-2.5) |  |
|  | Highest | 31 | 144 | 1 | 394 |
| High fat and protein diet (DP-2) | Lowest | 41 | 126 | 1.42(0.75-2.71) | 0.285 |
|  | Middle | 34 | 132 | 1.31(0.66-2.62) | 0.443*^95^ |
|  | Highest | 27 | 141 | 1 |  |
| Processed foods (DP-3) | Lowest | 18 | 148 | 1 |  |
|  | Middle | 32 | 137 | 1.97(1.01-3.86) | 0.048_3_*_97_ |
|  | Highest | 52 | 114 | 3.87(1.66-9.02) | 0.002** |

| 396 | |
| --- | --- |
| 398 | *Note: statistically significant factors associated with central obesity at p-value <0.05* and 0.001^**^* |
| 399 | 4. Discussion |
| 400 | The burden of NCDs is rising at an alarming rate, accounting for more than 70% of adult mortality |
| 401 | (40). Prediabetes and central obesity associated with metabolic syndrome are the most important |

402 risk factors for the development of overt NCDs (41). These diseases are in turn dependent on the
403 lifestyle and dietary consumption habits of individuals, which could be modified for a better health
404 condition (42). Hence, we conducted the current study to identify the role of dietary patterns in the
405 development of prediabetes (IBG) and central obesity among adults.
406 In this study, we found that 20.4% had IBG, 14.6% had central obesity, and 94% had an increased
407 risk for cardiovascular ~~complications~~ diseases based on the sex-specific WHCR cutoff point. This
408 indicates IBG is a common public health problem, especially among females, and it may be cross
409 linked with a comparable occurrence of central obesity (abdominal obesity) based on WC cutoff
410 points. Evidence from Ethiopia also showed that the prevalence of IBG ranges from 8% (43) to
411 12% (44). It should be emphasized that central obesity is a main risk factor for insulin resistance
412 and the development of IBG. Since 85% of NCD deaths occur in low- and middle income countries
413 (4), multilateral dietary and lifestyle interventions are required to halt central obesity and IBG.
414 This will be needed to decrease the incidence of metabolic risk factors before an overt metabolic
415 disease (cardiovascular or diabetes) happens. A higher prevalence of central obesity (39%) was
416 reported in southern Ethiopia (45).
417 In the present study, being female, higher socioeconomic class (AOR = 6.92; 95% CI: 2.91—16.5),
418 and physical inactivity (AOR = 21.1; 95% CI: 2.77—161.4) were found to be important predictors
419 of central obesity. A similar association was also identified with IBG, except that being physically
420 active is associated with a higher risk of IBG. This was also supported by findings from Ethiopia,
421 where females had a 9-times (AOR = 9.62; 95% CI: 4.84—19.12) (16) and 12-times (AOR =
422 12.93, 95% CI: 6.74—24.79) higher risk of obesity than males. A similar association was also
423 reported for socioeconomic class (46), where poor lifestyles and access to and consumption of
424 unhealthy foods are more common. In addition, physical activity and having regular physical
425 activity had a crucial for maintaining energy balance. A study indicated that adults who were
426 physically active were lower risk of central obesity (AOR = 0.87: p-value 0.174), emphasizing
427 the need for regular physical activity (46).
428 On the other hand, the role of non-exercise energy expenditures like working, walking, and others
429 should be encouraged as these are very crucial for maintaining optimal fat storage (47, 48).
430 However, the negative association with IBG could be explained by the fact that those with some
431 risk of cardiovascular complications might have poor blood sugar levels. This does not invalidate
432 the positive role of physical activity for optimal weight and blood glucose. Rather, it reflects the
433 characteristics of the study subjects.
434 Although there is evidence for the link between some dietary consumption habits and central
435 obesity and IBG (49), more concrete and context-specific evidence using a more robust approach
436 is needed to guide obesity and diabetes prevention strategies (50). Due to the complex and
437 multicollinear nature of the human diet and the limitations of individual food or nutrient analysis
438 for predicting a particular health outcome, dietary pattern analysis using a more robust statistical
439 approach is becoming a novel approach for better recommendations (12, 36). Hence, we derived
440 the dietary patterns and its association with the development of central obesity and IBG.
441 Those who reported consuming processed foods (AOR = 1.41; 95% CI: 0.57—3.48) and cereal
442 diets (AOR = 4.06; 95% CI: 1.87—8.82) were a major risk factor for the development of central
443 obesity. Other studies indicated that the top tercile of western pattern consumption (OR = 1.82;
444 95% CI: 1.16–2.87) increased the risk for obesity while a prudent pattern (AOR = 0.62; 95% CI:
445 0.40–0.96) reduced their risk for central obesity (51). On the other hand, those with a low
446 consumption of nutrient dense foods (animal sources, fruits, and vegetables) (AOR = 1.35; 95%
447 CI: 0.62—2.93) had a 35% increased risk of IBG compared to those with a higher tercile. This
448 could be explained by undernourished adults' metabolic limitations and an increased risk of insulin
449 insufficiency for glucose metabolism (52).
450 Other studies also showed that dietary patterns were highly correlated with abnormal blood glucose
451 status (r =	0.8)	(53). A	review of	the evidence	also found	that healthy, traditional, and
452 Mediterranean diets rich in sea foods, fruits, and vegetables were effective in preventing IBG (54).
453 Similarly, higher adherence to the unhealthy dietary pattern (saturated fats and meat) was more
454 likely to cause insulin resistance and IBG as a result (49). On the contrary, a vegetarian dietary
455 pattern reduced the risk of IBG (p-value below 0.05) (28). For instance, a study showed that a
456 higher tercile of the western dietary pattern was associated with an increased risk of obesity (AOR

457	= 1.80; 95% CI: 1.15–2.81) compared to a lower tercile (55).

458 The association between lower tercile consumption of nutrient-dense foods with IBG could be
459 related to the issue of food security and the risk of having unhealthy dietary patterns. For example,
460 a study found that food insecurity could lead to unhealthy dietary patterns and ultimately increase
461 the risk of central obesity (56). The current study depicted the role of dietary pattern in the
462 development of central obesity and prediabetes. These could help to design and indicate an
463 effective dietary strategy to prevent obesity and its associated consequences (57).
464 Limitations of the study
465 The current study is novel and would fill existing knowledge gaps regarding concrete evidence on
466 the relationship between dietary pattern and central obesity and IBG. Furthermore, we employed
467 a more reliable dietary assessment approach and dietary pattern analysis using a more appropriate
468 and feasible analytic strategy. However, the findings of this study should be in the light of some
469 methodological limitations. ~~However,~~First, in assessing food consumption, the potential for recall
470 bias and respondent bias could not be excluded, which might affect ~~the derived dietary patterns~~bias
471 the overall dietary consumptions. Moreover, the current study did not collect quantitative data on
472 daily intake to quantify the nutrient intake beyond food consumption. The current study mainly
473 focuses on the urban setting, where the disease burden could be higher. However, a comparative
474 study including urban and rural setting could better show the association in a very informative
475 way.
476 5. Conclusion and recommendations
477 Overall, the occurrence of central obesity, IBG, and the risk for ~~increased risk of~~ cardiovascular
478 ~~complications~~	diseases	were	prevalent	among	adults	in	the	study area. Dietary patterns
479 characterizing the overall diet of individuals are more predictive of these outcomes, which could
480 be replicated for other relevant diet-~~related~~ disease studies to inform policy and sstrategies
481 targeting the risks for non-communicable disease. Higher tercile consumption of nutrient dense
482 foods, high fat and protein diets, processed foods, and cereal diets are important risk factors for
483 central obesity and IBG. Optimal consumption of a diversified diet from animal sources, fruits,
484 vegetables, cereals, and protein-rich foods could play a great role in preventing the occurrence of
485 metabolic syndromes, while consumption of processed foods and alcoholic drinks should be kept
486 to a minimum. The findings of this study will inform the existing policies and programs targeting
487 non-communicable disease prevention in Ethiopia. We strongly recommend regional health
488 bureaus, non-governmental organizations working on nutrition, and other concerned bodies to
489 strategically implement appropriate behavioral change communication and agricultural strategies
490 healthy diet for the wider community.
491 **Declarations**
492 **Ethical approval and consent to participate**
493 Ethical approval was obtained from the Institutional Research Ethical Review Board of Dire Dawa
494 University. Written informed assent was obtained from all adults This research was conducted in
495 accordance with the Helsinki Declaration for protecting human study subjects.
496 **Consent to publish**
497 Not applicable
498 **Acknowledgment**
499 We are grateful to Dire Dawa University and Dire Dawa Health Bureau for their collaborations
500 and support for the successful completion of the study. Our gratitude also goes to the respective
501 health	bureaus, respondents, data collectors, and supervisors	for	their sincere	help	and
502 collaboration for the successful completion of the research.
503 Author contribution
504 AO- participated in conceptualization, fund acquisition, designing the study, data curation,
505 resource management, methodology, validation, supervision, project administration, data analysis,
506 writing ~~a report~~the manuscript, and reviewing and approving the manuscript. AA, AH, ~~and~~ BA,
507 ID, AAU, DF, MY, ZT, and SA: participated in conceptualization, validation, supervision,
508 methodology, data preparation, and data visualization. ~~, and formal data analysis.~~ In addition, AO
509 hass substantially contributed to cleaning, preparing, performing the whole statistical analysis,
510 preparation of tables and graphs, writing ~~the~~ ~~, drafting~~and drafting the manuscript, critically
511 reviewing, revising, and editing the ~~drafted~~ manuscript. All authors have reviewed and approved
512 the final manuscript and decided on the journal to submit to.
513 **Data Availiability**
514 All relevant data are included within the manuscript and its supporting Information files.
515 **Funding**
516 This study was funded by Dire Dawa University, under annual competitive research grant. The
517 funders had no role in study design, data collection and analysis, the decision to publish, or the
518 preparation of the manuscript.
519 **Competing interests**
520 The authors have declared that no competing interests exist.
521 **Abbreviations:**
522 CI~~—~~–Confidence Interval; DP~~—~~– Dietary Patterns; FFQ~~—~~–Food	Frequency	Questionnaire;
523 GPAQ~~—~~–General Physical Activity Questionnaire; IBG; HC~~—~~–Hip Circumference; Impaired
524 Blood	Glucose; K~~—~~–Sampling	Interval; METS~~—~~–Metabolic Equivalents; PCA~~—~~–Principal
525 Component Analysis; NCDs~~—~~–Non communicable Diseases; SD~~—~~–Standard Deviations; VIF~~—~~
526 –Variance	Inflation	Factor; WC~~—~~–Waist	Circumference; WHCR~~—~~–Waist~~—~~–to~~—~~–Hip
527 Circumference Ratio; WHO ~~—~~–World Health Organization 528

529

530

531

532

533	6. References

534	1.	LNeuhouser M. The Importance of Healthy Dietary Patterns in Chronic Disease

| 535 | Prevention. *Nutrition Research* (2019) 70(Commentary):3-6. |
| --- | --- |
| 536 | 2. Tapsell LC, Neale EP, Probst Y. Dietary Patterns and Cardiovascular Disease: Insights and |
| 537 | Challenges for Considering Food Groups and Nutrient Sources. (2019). |
| 538 | [Https://Doi.Org/10.1007/S11883-019-0770-1.](https://doi.org/10.1007/S11883-019-0770-1) *Curr Atheroscler Rep* (2019) 21(9):1-8. |
| 539 | 3. Ojo O. Nutrition and Chronic Conditions. *Nutrients* (2019) 11(2):459. |
| 540 | 4. World Health Organisation (WHO). Noncommunicable Diseases Geneva, |
| 541 | Switherland(2018) [cited 2021 Jan 27]. |
| 542 | 5. Gouda HN, Charlson F, Sorsdahl K, Ahmadzada S, Ferrari AJ, Erskine H, et al. Burden of |
| 543 | Non-Communicable Diseases in Sub-Saharan Africa, 1990–2017: Results from the Global Burden |
| 544 | of Disease Study 2017. *The lancet Global Health* (2019) 7(10):1375-E87. |
| 545 | 6. World Health Organization (WHO). *Global Status Report on Noncommunicable Diseases* |
| 546 | *2010*. Geneva: World Health Organization (2011). p. 162. |
| 547 | 7. Santaguida P, Balion C, Hunt D, Morrison K, Gerstein H, P Raina LB, et al. Diagnosis, |
| 548 | Prognosis, and Treatment of Impaired Glucose Tolerance and Impaired Fasting Glucose: |
| 549 | Summary. *AHRQ Evidence Report Summaries* (2005). |
| 550 | 8. Dhawan D, Sharma S. Abdominal Obesity, Adipokines and Non-Communicable Diseases. |
| 551 | *The Journal of steroid biochemistry and molecular biology* (2020) 203:105737. |
| 552 | 9. GBD 2017 Diet Collaborators. Health Effects of Dietary Risks in 195 Countries, 1990– |
| 553 | 2017: A Systematic Analysis for the Global Burden of Disease Study 2017. *The Lancet* (2019) |
| 554 | 393(10184):1958-72. |
| 555 | 10. Aleksandrova K, Giuseppe Rd. Diet, Healthy Aging, and Cognitive Function. *Analysis in* |
| 556 | *Nutrition Research*. Springer (2019). |
| 557 | 11. Abdi H, Williams LJ. Principal Component Analysis. *Wiley interdisciplinary reviews:* |
| 558 | *computational statistics* (2010) 2(4):433-59. |
| 559 | 12. Agnoli C, Krogh V. Dietary Pattern Analysis. *Analysis in Nutrition Research*. Springer |
| 560 | (2019). |
| 561 | 13. Abebe SM, Andargie G, Shimeka A, Alemu K, Kebede Y, Wubeshet M, et al. The |
| 562 | Prevalence of Non-Communicable Diseases in Northwest Ethiopia: Survey of Dabat Health and |
| 563 | Demographic Surveillance System *BMJ Open* (2017) 7(10):1-7. |
| 564 | 14. Misganaw A, Mariam DH, Ali A, Araya T. Epidemiology of Major Non-Communicable |
| 565 | Diseases in Ethiopia: A Systematic Review. *J Health Popul Nutr* (2014) 32 (1):1-13. |
| 566 | 15. Kassie AM, Abate BB, Kassaw MW. Prevalence of Overweight/Obesity among the Adult |
| 567 | Population in Ethiopia: A Systematic Review and Meta-Analysis. *BMJ Open* (2020) |
| 568 | 10(8):e039200. doi: 10.1136/bmjopen-2020-039200. |
| 569 | 16. Molla MD, Wolde HF, Atnafu A. Magnitude of Central Obesity and Its Associated Factors |
| 570 | among Adults in Urban Areas of Northwest Ethiopia. *Diabetes, Metabolic Syndrome and Obesity:* |
| 571 | *Targets and Therapy* (2020) 13:4169-78. |
| 572 | 17. Chooi YC, Ding C, Magkos F. The Epidemiology of Obesity. *Metabolism* (2019) 92:6-10. |
| 573 | doi: [https://doi.org/10.1016/j.metabol.2018.09.005.](https://doi.org/10.1016/j.metabol.2018.09.005) |
| 574 | 18. Bastien M, Poirier P, Lemieux I, Després Jp. Overview of Epidemiology and Contribution |
| 575 | of Obesity to Cardiovascular Disease. Prog Cardiovasc Dis. 2014;56:369–81. |
| 576 | 19. Gabir, Mm, Hanson, Rl, Dabelea, D. The 1997 American Diabetes Association and 1999 |
| 577 | World Health Organization Criteria for Hyperglycemia in the Diagnosis and Prediction of |
| 578 | Diabetes. Diabetes Care 2000; 23: 1108–1112. |

| 579 | 20. Shaw, Je, Zimmet, Pz, De Courten, M. Impaired Fasting Glucose or Impaired Glucose |
| --- | --- |
| 580 | Tolerance. What Best Predicts Future Diabetes in Mauritius? Diabetes Care 1999; 22: 399–402. |
| 581 | 21. Forouhi NG, Balkau B, Borch-Johnsen K, Dekker J, Glumer C, Qiao Q, et al. The |
| 582 | Threshold for Diagnosing Impaired Fasting Glucose: A Position Statement by the European |
| 583 | Diabetes Epidemiology Group. *Diabetologia* (2006) 49(5):822-7. doi: 10.1007/s00125-006-0189- |
| 584 | 4. |
| 585 | 22. Hosseini-Esfahani F, Koochakpoor G, Daneshpour MS, Sedaghati-khayat B, Mirmiran P, |
| 586 | Azizi F. Mediterranean Dietary Pattern Adherence Modify the Association between Fto Genetic |
| 587 | Variations and Obesity Phenotypes. *Nutrients* (2017) 9(10):1064. |
| 588 | 23. L.Strate L, R.Keeley B, YinCao, KanaWu, L.Giovannucci E, T.Chan A. Western Dietary |
| 589 | Pattern Increases, and Prudent Dietary Pattern Decreases, Risk of Incident Diverticulitis in a |
| 590 | Prospective Cohort Study. *Gastroenterology* (2017) 152(5):1023-30 e2. |
| 591 | 24. Li M, Shi Z. Dietary Pattern During 1991–2011 and Its Association with Cardio Metabolic |
| 592 | Risks in Chinese Adults: The China Health and Nutrition Survey. *Nutrients* (2017) 9(11):1218. |
| 593 | 25. Mu M, Xu L-F, Hu D, Wu J, Bai M-J. Dietary Patterns and Overweight/Obesity: A Review |
| 594 | Article. *Iran J Public Health* (2017) 46(7):869-76. |
| 595 | 26. Tesfaye T, Zeleke TM, Alemu W, Argaw D, Bedane TK. Dietary Diversity and Physical |
| 596 | Activity as a Risk Factors of Abdomenal Obesity among Adults in Dilla Town, Ethiopia. *PLOs* |
| 597 | *One* (2020) 15(7):e0236671. |
| 598 | 27. Ghachem A, Brochu M, Dionne IJ. Differential Clusters of Modifiable Risk Factors for |
| 599 | Impaired Fasting Glucose Versus Impaired Glucose Tolerance in Adults 50 Years of Age and |

600	Older. *Ther	Adv	Chronic	Dis*	(2019)	10(eCollection). doi: 601 [https://doi.org/10.1177/2040622319854239.](https://doi.org/10.1177/2040622319854239)

602 28. Amini M, Esmaillzadeh A, Shafaizadeh S, Behrooz J, Zare M. Relationship between Major 603 Dietary Pattern and Metabolic Syndrome among Individuals with Impaired Blood Glucose. 604 *Nutrition* (2010) 26(6):986-92.

605 29. World Health organization. Global Physical Activity Questionnaire (Gpaq) Analysis 606 Guide. Geneva: WHO, (2011) 1.

607 30. Regassa IF, Endris BS, Habtemariam E, Hassen HY, Ghebreyesus SH. Development and 608 Validation of Food Frequency Questionnaire for Food and Nutrient Intakes of Adults in Butajira, 609 Southern Ethiopia. *Journal of nutritional science* (2021) 10.

610 31. George D, Mallery P. Reliability Analysis. *Ibm Spss Statistics 25 Step by Step*. Routledge 611 (2018). p. 249-60.

612 32. Perini	TA, Oliveira	GL	OJ, Oliveira	FP.,. Technical	Error	of	Measurement	in 613 Anthropometry. ( 2005) 11:81-5.

614 33. Oumer A, Abraham M, Nuri A. Predictors of Major Dietary Patterns among Pregnant 615 Women Attending Public Health Facilities in Eastern Ethiopia: A New Epidemiological Approach. 616 *Frontiers in Nutrition* (2022) 9. doi: 10.3389/fnut.2022.855149.

617 34. Oumer A, Abraham M, Nuri A. Predictors of Major Dietary Patterns among Pregnant 618 Women Attending Public Health Faility in Eastern Ethiopia: A New Epidemiological Approach. 619 *Frontier in Nutrition* (2022) Accepted Manuscript.

620 35. Plaza-Díaz J, Molina-Montes E, Soto-Méndez MJ, Madrigal C, Hernández-Ruiz Á, Valero 621 T, et al. Clustering of Dietary Patterns and Lifestyles among Spanish Children in the Esnupi Study 622 †. *Nutrients* (2020) 12(9):2536.

623 36. Hu FB. Dietary Pattern Analysis: A New Direction in Nutritional Epidemiology. *Current*

624 *opinion in lipidology* (2002) 13(1):3-9.

625 37. Genuth S, Alberti KG, Bennett P, Buse J, Defronzo R, Hahn R, et al. Expert Committee on 626 the Diagnosis and Classification of Diabetes Mellitus: Follow-up Report on the Diagnosis of 627 Diabetes Mellitus. Diabetes Care 26:3160–3167. (2003).

628 38. Ashwell M, Gibson S. Waist to Height Ratio Is a Simple and Effective Obesity Screening 629 Tool for Cardiovascular Risk Factors: Analysis of Data from the British National Diet and 630 Nutrition Survey of Adults Aged 19–64 Years. *Obesity facts* (2009) 2(2):97-103.

631 39. Association GAotWM. World Medical Association Declaration of Helsinki: Ethical 632 Principles for Medical Research Involving Human Subjects. *Am J Dent* (2014) 81(3):14-8.

633 40. Ethipian Public	health institute	(EPHI). *Steps	Survey	on	Risk	Factors	for	Non* 634	*Communicable Diseases and Prevlence of Selected Ncds Ethiopia.* In: Nutrition, editor. Addis	635 Ababa: EPHI (2016). p. 2-25.

636 41. Peters R, Ee N, Peters J, Beckett N, Booth A, Rockwood K, et al. Common Risk Factors 637 for Major Noncommunicable Disease, a Systematic Overview of Reviews and Commentary: The 638 Implied Potential for Targeted Risk Reduction. *Therapeutic advances in chronic disease* (2019) 639 10:2040622319880392.

640 42. Mudie K, Jin MM, Kendall L, Addo J, dos-Santos-Silva I, Quint J, et al. Non- 641 Communicable Diseases in Sub-Saharan Africa: A Scoping Review of Large Cohort Studies. 642 *Journal of global health* (2019) 9(2).

643 43. Tesfaye T, Shikur B, Shimels T, Firdu N. Prevalence and Factors Associated with Diabetes 644	Mellitus and Impaired Fasting Glucose Level among Members of Federal Police Commission	645 Residing in	Addis	Ababa, Ethiopia. *BMC	Endocrine	Disorders*	(2016)	16(1):68. doi: 646 10.1186/s12902-016-0150-6.

647 44. Worede A, Alemu S, Gelaw YA, Abebe M. The Prevalence of Impaired Fasting Glucose 648 and Undiagnosed Diabetes Mellitus and Associated Risk Factors among Adults Living in a Rural 649 Koladiba Town, Northwest Ethiopia. *BMC Res Notes* (2017) 10(1):251.

650 45. Tekalegn Y, Solomon D, Sahiledengle B, Assefa T, Negash W, Tahir A, et al. Prevalence 651 of Central Obesity and Its Associated Risk Factors among Adults in Southeast Ethiopia: A 652 Community-Based Cross-Sectional Study. *PloS one* (2022) 17(8):e0265107.

653 46. Owolabi EO, Ter Goon D, Adeniyi OV. Central Obesity and Normal-Weight Central 654 Obesity among Adults Attending Healthcare Facilities in Buffalo City Metropolitan Municipality, 655 South Africa: A Cross-Sectional Study. *Journal of Health, Population and Nutrition* (2017) 656 36(1):54. doi: 10.1186/s41043-017-0133-x.

657 47. Herrmann SD, Willis EA, Honas JJ, Lee J, Washburn RA, Donnelly JE. Energy Intake, 658 Nonexercise Physical Activity, and Weight Loss in Responders and Nonresponders: The Midwest 659 Exercise Trial 2. *Obesity* (2015) 23(8):1539-49.

660 48. Melanson E. The Effect of Exercise on Non‐ Exercise Physical Activity and Sedentary 661 Behavior in Adults. *Obesity Reviews* (2017) 18:40-9.

662 49. Mazidi M, Kengne AP, Mikhailidis DP, Toth PP, Ray KK, Banach M. Dietary Food 663 Patterns and Glucose/Insulin Homeostasis: A Cross-Sectional Study Involving 24,182 Adult 664 Americans. *Lipids in health and disease* (2017) 16(1):192. doi: 10.1186/s12944-017-0571-x.

665 50. Bekele TH, De Vries JJ, Trijsburg L, Feskens E, Covic N, Kennedy G, et al. Methodology 666	for Developing and Evaluating Food-Based Dietary Guidelines and a Healthy Eating Index for	667 Ethiopia: A Study Protocol. *BMJ Open* (2019) 9(7):e027846.

668 51. Paradis, Am., Godin, G., Pérusse, L. Et Al. Associations between Dietary Patterns and 669 Obesity Phenotypes. Int J Obes 33, 1419–1426 (2009). [Https://Doi.Org/10.1038/Ijo.2009.179.](https://doi.org/10.1038/Ijo.2009.179)

670 52. Barker DJ, Thornburg KL. Placental Programming of Chronic Diseases, Cancer and 671 Lifespan: A Review. *Placenta* (2013) 34(10):841-5.

672 53. Hlaing HH, Liabsuetrakul T. Dietary Intake, Food Pattern, and Abnormal Blood Glucose 673 Status of Middle-Aged Adults: A Cross-Sectional Community-Based Study in Myanmar. *Food* 674 *Nutr Res* ( 2016) 60::1-8.

675 54. Maghsoudi Z, Azadbakht L. How Dietary Patterns Could Have a Role in Prevention, 676 Progression, or Management of Diabetes Mellitus? Review on the Current Evidence. *J Res Med* 677 *Sci* (2020) 17(7):694–709.

678 55. Zhang X, Li Y, Liu a, Zhang Q, Hu X, S. Et Al. (2012)Dietary Pattern and Its Association 679 with the Prevalence of Obesity and Related Cardiometabolic Risk Factors among Chinese Childre 680 Dietary Pattern and Its Association with the Prevalence of Obesity and Related Cardiometabolic 681 Risk Factors among Chinese Children. Plos One 7(8): E43183.

| 682 | [Https://Doi.Org/10.1371/Journal.Pone.0043183.](https://doi.org/10.1371/Journal.Pone.0043183) |
| --- | --- |
| 683 | 56. Morales ME, Berkowitz SA. The Relationship between Food Insecurity, Dietary Patterns, |
| 684 | and Obesity. *Current Nutrition Reports* (2016) 5(1):54-60. doi: 10.1007/s13668-016-0153-y. |
| 685 | 57. Seifu CN, Fahey PP, Hailemariam TG, Frost SA, Atlantis E. Dietary Patterns Associated |
| 686 | with Obesity Outcomes in Adults: An Umbrella Review of Systematic Reviews. *Public Health* |
| 687 | *Nutrition* (2021) 24(18):6390-414. |

688

Response to Reviewers

## PONE-D-22-28499

*Major dietary patterns of community dwelling adults and their associations with impaired blood glucose and central obesity in Eastern Ethiopia: diet-disease exploration study*

# Dear editor and reviewers

Thank you very much for having such detail and valuable comments on our work. We have checked your comments and suggestion in more detail and have included them in the revised submission. All the changes are showed in track changes. Hence, we found it that giving a detail response for each point is not necessary. Thus, we only responded to question which need clarification ad included the requested changes in the revised manuscript simply.

On the title, the comment was reasonable and we amended the title accordingly. We also amended the comments on the abstract as it refers to percent based on WHCR cutoff point. These finding is discussed in more detail in the discussion part.

We specified the study design as survey or cross-sectional study design and the study period as “September 10 to November 06”.

You declared the final sample size as 526 in the section of sample size determination. Why you did you decide on 501?

The estimated minimum sample size required for this study was 526. However, the achieved response rate was 95% where a total 25 respondents were non-responders. They were not volunteer to participate in the study. The difference is due to this.

A separate multivariable model was done for IBG and central obesity as this is shown in Table 4 and 5. This is also clearly indicated there.

The interpretation of the odds ratio had some errors and we accepted the comments from the reviewers. Hence, we thoroughly edited the suggestions in way that the Odds ratio could be

interpreted accordingly. Hence, we interpreted it as increased occurrence or odds of IBG or central obesity as it is indicated in the revised version. The comments are well accepted.

Computing COR is not conclusive to determine determinants of IBG.

Here, we run both the crude and adjusted odds ratio. This could allow us to clearly indicate the statistical analysis and the reader in a very clear and reproducible way. Since we also clearly reported the adjusted association, this is very informative than the crude one. Hence, we made our conclusion and recommendations based on the adjusted associations. Presenting the crude one makes the paper more holistic and reproducible.

*This discussion about the association between dietary pattern and central obesity and IBG in this study is not adequate. In addition, it is not exhaustive. You need to compare with similar finding and identify similar and unique variables. If this study identifies unique variable you need to have possible justification.*

Here, as you mentioned the objective of the study is like that and we tried our best to present the discussion in a very clear and sequential manner. We started with IBG and central obesity, then their association with empirical dietary patterns. The major challenge was lack of such evidence in Ethiopia and Africa as well. That is why we mainly used gray literatures and fats to support and explain our results. But we have tried our best to modify the discussion as per the reviewers’ suggestions and authors’ view. These are indicated in revised manuscript.

How do conclude that 94% had increased risk for cardiovascular complication? Because, you did not do any statistics test regarding risk for cardiovascular complication.

This is based on the WHO cutoff value for WHCR, which is predictive of the increased risk for cardiovascular complications. These has been indicated in many nutritional epidemiological studies and are very predictive for CVD risks.

Thanks for having a comment on the limitation of the study. Since mentioning the relevant limitation of the study is important to interpreted the study finding and its implication, we have mentioned and explained these limitations there.

Lastly, we have modified the recommendations to be specific to the specific body with a clear message. This is indicated in the conclusion part of the manuscript.

.... higher among those who were physically active (AOR = 1.60; 95% CI: 0.90–2.85) (Table 5).

This result seems strange when we see the direct relationship between exercise and IBG. However, as studies reported and we tried to discuss in the discussion, majority of the exercisers usually have some sort of non-communicable disease and obesity. In such cases, the individual might have an already IBG despite exercise. We noted this result in more detail there.

*Thank you very much!*

*Authors*
