# Supplementary material for: Major dietary patterns of community dwelling adults and their associations with impaired blood glucose and central obesity in Eastern Ethiopia: Diet-disease epidemiological study
Source: PLoS One. 2023 Apr 20;18(4):e0283075. doi: 10.1371/journal.pone.0283075 (PMC10118135; doi:10.1371/journal.pone.0283075)
Supplement: Attachment — Submitted filename: Response to Reviewers.docx [file pone.0283075.s007.docx]

Dear reviewer

Thanks for having your second-round revisions. We have corrected the comments as indicated in the revised version.

COR and AOR is distinctly different.

**Response:** the COR and AOR are different and that is why we presented them for Central obesity and IBG separately in Table 3. Similarly, we clearly resented the AOR in the next table 4. The one in the table 3 is the unadjusted Bivariable logistic regression out put while the next table presented the adjusted associations.

Row 1 of the table is COR which Denotes for crude ODDS ratio. If it is Adjusted you must write AOR. Except that your justification to include crude odds ratio is acceptable.

**Response:** these was due to error and we have corrected this one in the revised version.

I need a clear-cut interpretation regarding ODDS ratio. If you did Hazard ratio you can interpret as risk without hesitation.

Response: The major comment was ack of consistent interpretation of the odds ratio. Considering this, we have thoroughly addressed these interpretations in well-structured manner as likelihood rather than relative risk or hazard ratio.

Reviewer #2: Computing COR is not conclusive to determine determinants of IBG.
Row 1 of the table is COR, which denotes for crude ODDS ratio. If it is adjusted, you must write AOR. Except that your justification to include crude odds ratio is acceptable. Line 381.
You need to scrutinize the interpretation of the ODDS result in the discussion section. There are couples of mistake there.
**Response:** The major comment was ack of consistent interpretation of the odds ratio. Considering this, we have thoroughly addressed these interpretations in well-structured manner as likelihood rather than relative risk or hazard ratio.

“Central obesity (AOR = 0.87: p-value 0.174)” Line 420 to 421, from where you brought this finding? Table 4 shows this finding “21.1(2.77-161.4)” the reference population is a physically active group.

**Response**: as indicated this finding was obtained from ref no 46 as indicated there.

ODDS ration interpretation. In the discussion and Abstract section. I need a clear-cut interpretation regarding ODDS ratio. If you did Hazard ratio, you can interpret as risk without hesitation.

**Response:** The major comment was ack of consistent interpretation of the odds ratio. Considering this, we have thoroughly addressed these interpretations in well-structured manner as likelihood rather than relative risk or hazard ratio.
